# Supplementary material for: EBAG9 silencing exerts an immune checkpoint function without aggravating adverse effects
Source: Mol Ther. 2022 Jul 12;30(11):3358–78. doi: 10.1016/j.ymthe.2022.07.009 (PMC9637585; doi:10.1016/j.ymthe.2022.07.009)
Supplement: Document S1. Figures S1–S10 and supplemental methods [file mmc1.pdf]

YMTHE, Volume *30*

Supplemental Information

EBAG9 silencing exerts an immune checkpoint function without aggravating adverse effects

Anthea Wirges, Mario Bunse, Jara J. Joedicke, Eric Blanc, Venugopal Gudipati, Michael W. Moles, Hiroshi Shiku, Dieter Beule, Johannes B. Huppa, Uta E. Höpken, and Armin Rehm

Supplemental information

Inventory of Supplemental information:

Supplementary Figures 1-10 and Figure legends

Supplementary Table S1, S2; Legends

Supplementary Methods, Reagents Table

Supplementary Figures and Figure legends

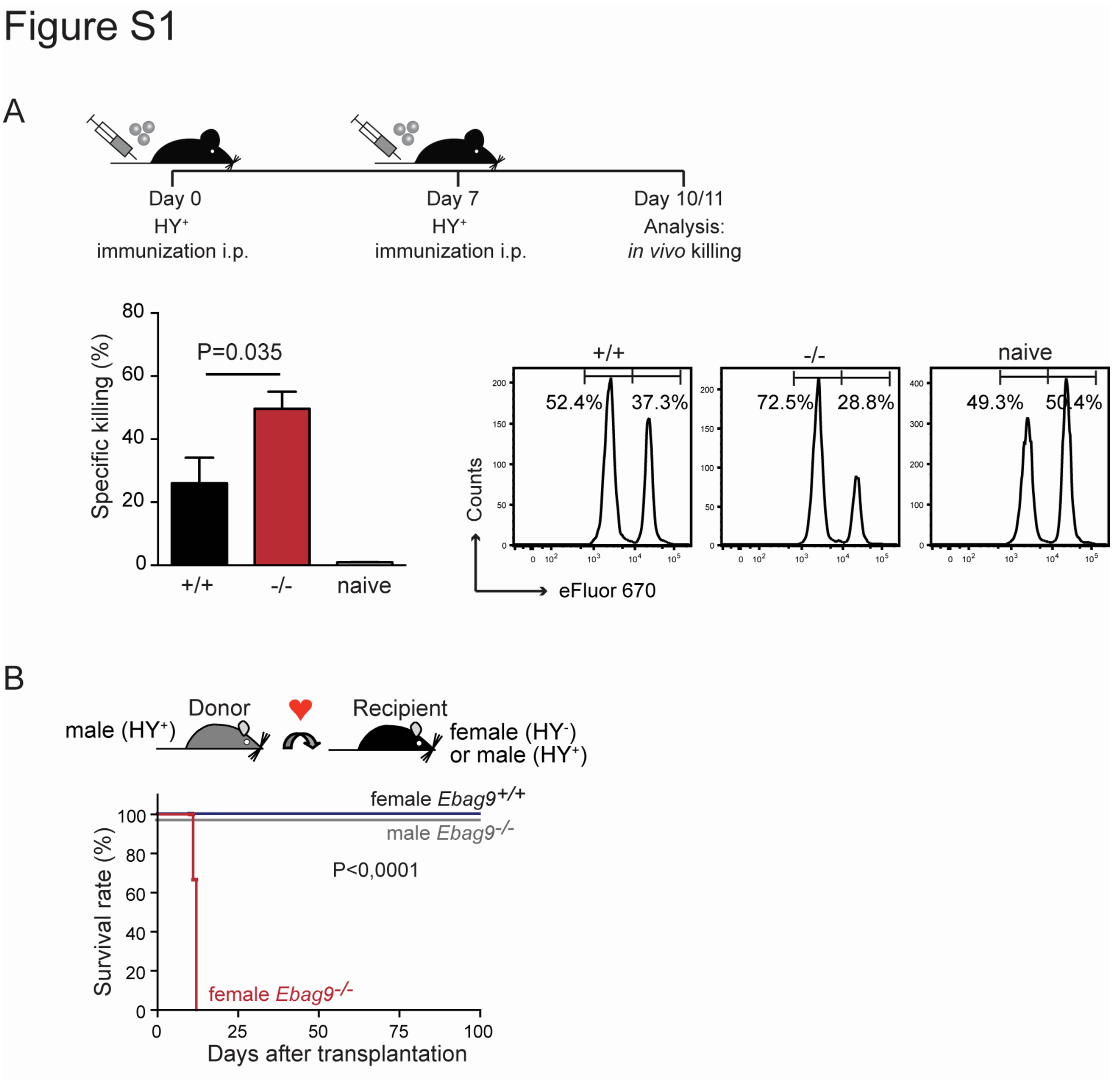

Figure S1: Enhanced anti-HY specific cytotoxic response of *Ebag9*$^{-/-}$ mice

(A) Female C57.BL/6 Wt (+/+) and *Ebag9*$^{-/-}$ (-/-) mice were immunized twice i.p. with 5x10^6 male splenocytes (B6). At day 10, animals were challenged with equal numbers of female (HY^-) and male (HY^+) splenocytes labeled with different amounts of eFluor 670 (female low eFluor 670 and male high eFluor 670). The ratio between the two target populations was determined by flow cytometry 16 hrs later and expressed as specific killing of male splenocytes in percent. Naive, non-immunized mice were used as control (control ratio). Bars show mean values ± SEM of n=2 independent assays. One representative example is shown on the right (Wt, n=8; *Ebag9*$^{-/-}$, n=7; naive, non-immunized C57.BL/6 mice, n=3). Percentages of HY^- and HY^+ splenocytes are indicated. An unpaired Student's t-test was employed. To calculate the specific killing, the following formula was applied:

% specific killing = [1 - (control ratio / experimental ratio)] x 100

Ratio = % low eFluor 670 peak / % high eFluor 670 peak

(Low = w/o peptide, high = peptide-pulsed)

(B) Female C57.BL/6 Wt (+/+) or *Ebag9*$^{-/-}$ (-/-) mice were heterotopically transplanted with male donor hearts (C57.BL/6, Wt). Male cardiac allografts were acutely rejected in *Ebag9*$^{-/-}$ recipients at mean survival times of 12 ± 2 days (n=3), Wt mice (n=4) accepted the grafts over the observation period of 100 days. Syngeneic male heart transplants showed no rejection in male recipients. n=2 independent experiments, p values were determined with log-rank test (Mantel-Cox test).

Figure S2

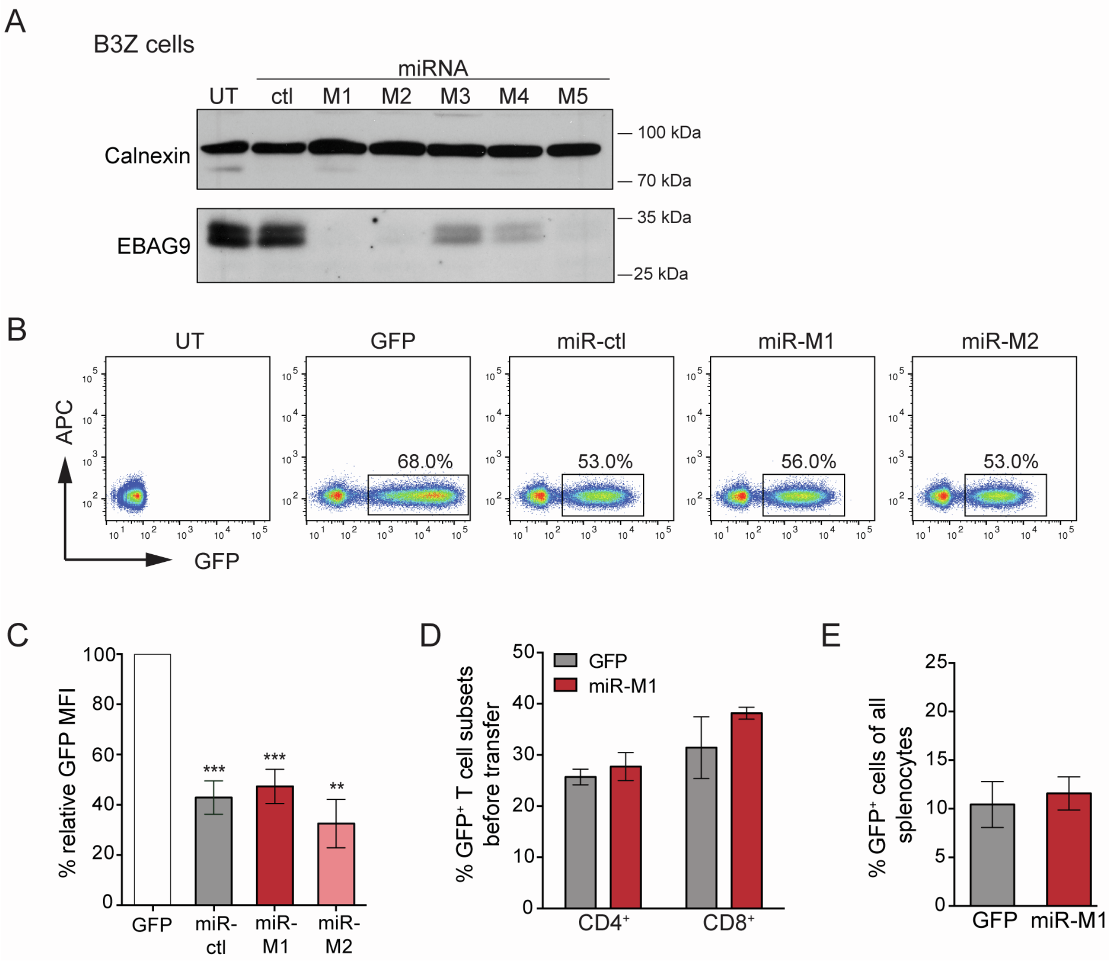

Figure S2: Efficacy of miRNA-mediated EBAG9 silencing in mouse T cells

(A) Target site validation for miRNA-mediated silencing of EBAG9. B3Z cells were retrovirally transduced with MP71 vectors encoding GFP and various miRNAs targeting different regions of the *Ebag9* transcript (miR-M1 – M5). Non-transduced (UT) cells and the miR-ctl without any endogenous target sequence were used as controls. GFP^+ cells were sorted by FACS and analyzed by Western blot using rabbit anti-EBAG9 antibody. Calnexin served as a loading control.

(B) Efficient transduction of primary mouse T cells. Flow cytometric analysis of retrovirally transduced mouse T cells at day 6 after activation. Positively transduced cells express GFP. Transduction rates are indicated as numbers in the gate.

(C) Transduction of primary mouse T cells with vectors carrying GFP and the miRNAs led to reduced GFP expression levels. MFI is depicted as compared to GFP alone. Bars represent mean values ± SEM of n=7 experiments with n=4 (miR-M2) and n=8 (GFP, miR-ctl, -M1) samples per group. **p<0.01, *p<0.001. A one sample t-test was applied.

(D) Transduced mouse T cells (GFP^{+}) were analyzed by flow cytometry. GFP^{+} cells were co-stained for CD4^{+} and CD8^{+}. Bars represent mean values ± SEM of n=3 experiments with n=3 samples per group.

(E) Twenty days after T cell transfer into *Rag2*$^{-/-}$ mice, mice were sacrificed. T cells were analyzed by flow cytometry. Bars represent mean values ± SEM of n=5 experiments with n=10 (GFP) and n=15 (miR-M1) mice per group. In D) and E), a Mann-Whitney U test was applied. P-values were not significant.

Figure S3

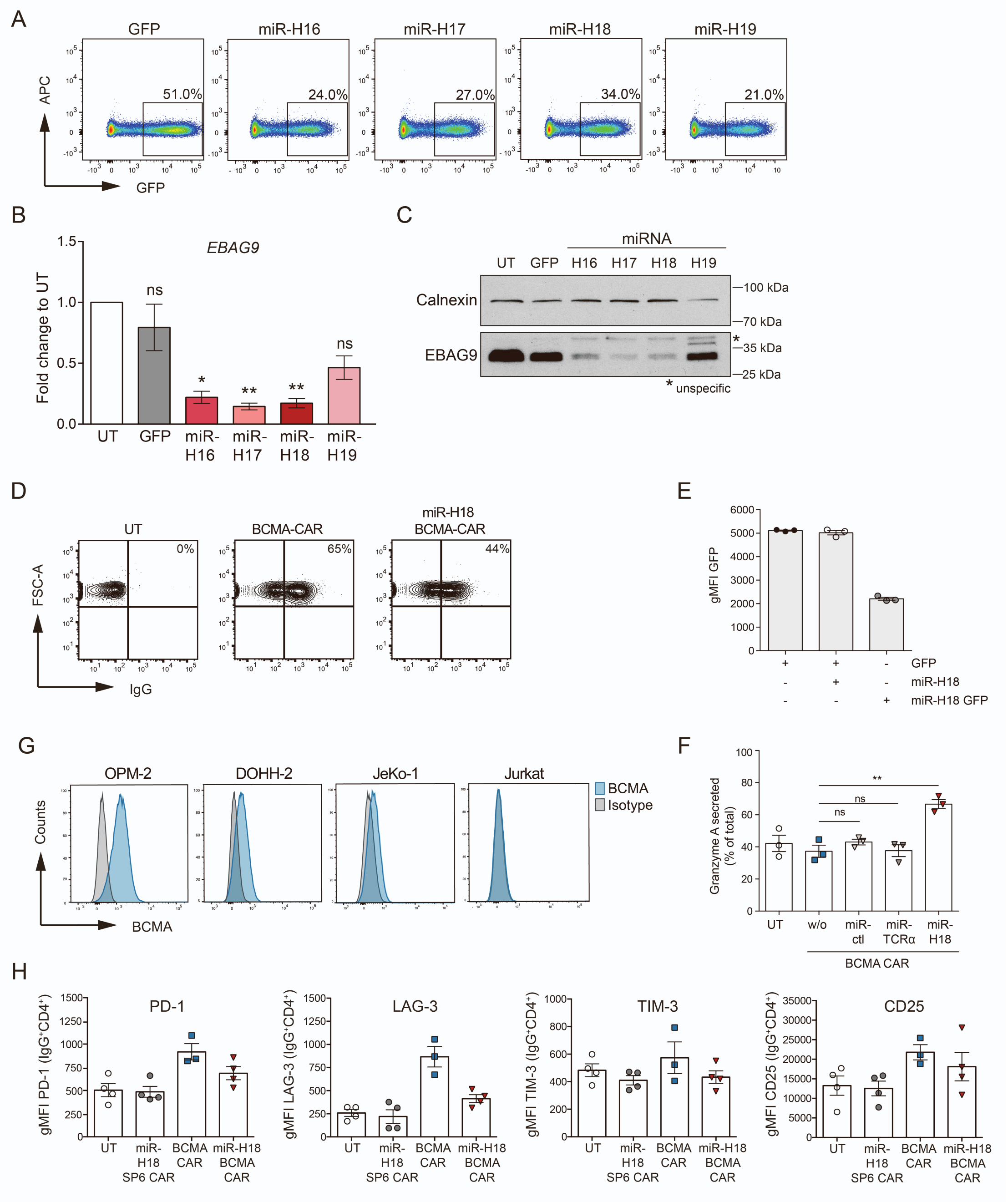

Figure S3: Efficacy of miRNA-mediated EBAG9 silencing in human T cells

(A) Retroviral transduction of human Jurkat cells with different GFP-encoding MP71 vectors expressing various miRNAs directed against human EBAG9. GFP expression was measured by flow cytometry. Transduction rates are indicated by numbers in the gates.

(B) miRNA-mediated reduction of human *EBAG9* mRNA. Jurkat T-ALL cells were retrovirally transduced with vectors encoding for GFP and various miRNAs targeting the *EBAG9* transcript (miR-H16 – H19). Transduced GFP^+ T cells were sorted by FACS and *EBAG9* mRNA expression was analyzed by RT-qPCR. Non-transduced (UT) cells were set arbitrarily at 1. Bars represent mean values ± SEM of n=2 experiments with n=3 samples per group. *$p<0.05$, **$p<0.01$; ns, not significant. A one-sample t-test was applied.

(C) miRNA-mediated reduction of EBAG9 protein level. Transduced T cells were raised as in (B). GFP^+ T cells were sorted by FACS and analyzed by Western blot using a rabbit anti-EBAG9 antibody. One representative Western blot out of three experiments is shown. Calnexin served as a loading control.

(D) Frequency and CAR surface expression levels of T cells ($CD3^+$) at day 7 after retroviral transduction. Gates were set relative to anti-IgG reactivity on untransduced (UT) T cells, gMFI values of cells in the IgG^+ gated population were determined using FloJo software. This FACS contour plot corresponds to Figure 2D).

(E) Reduced transgene expression is caused by the retroviral vector design carrying the miRNA intronically. Jurkat cells were transduced with GFP as indicated. Positively transduced cells were then sorted for GFP-expression and further transduced with a control vector or a miR-H18 containing vector. One group received miR-H18 GFP only. Bars represent mean values ± SEM of one experiment with n=3 samples per group.

(F) Control miRNAs have no impact on the release of granzyme A. Granzyme A release was induced by re-stimulation of $CD8^+$ BCMA CAR CAR T cells with anti-human CD3 and CD28 antibodies for 4 hrs. Enzymatic activities in the supernatant were measured in duplicates. Values show the release in percentages relative to the

total content. Bars represent mean ± SEM of one experiments with n=3 independent donors per group. **p<0.01; ns, not significant. A one-sample t-test was applied.

(G) Flow cytometric analysis of BCMA surface expression on MM (OPM-2) and B-NHL (DOHH-2, JeKo-1) cell lines. A representative histogram for each cell line is shown. The BCMA-negative Jurkat cell line served as a control.

(H) Quantification of flow cytometric analysis of key immune markers for T cell function. $CD4^+$ CAR T cells (IgG^+ $CD4^+$) at day 7 after activation were co-stained with anti-PD-1, anti-LAG-3, anti-TIM-3, and anti-CD25 antibodies. Bars represent mean ± SEM of one experiment with n=3 (BCMA CAR) and n=4 (UT, miR-H18 SP6 CAR, miR-H18 BCMA CAR) independent donors per group.

Figure S4

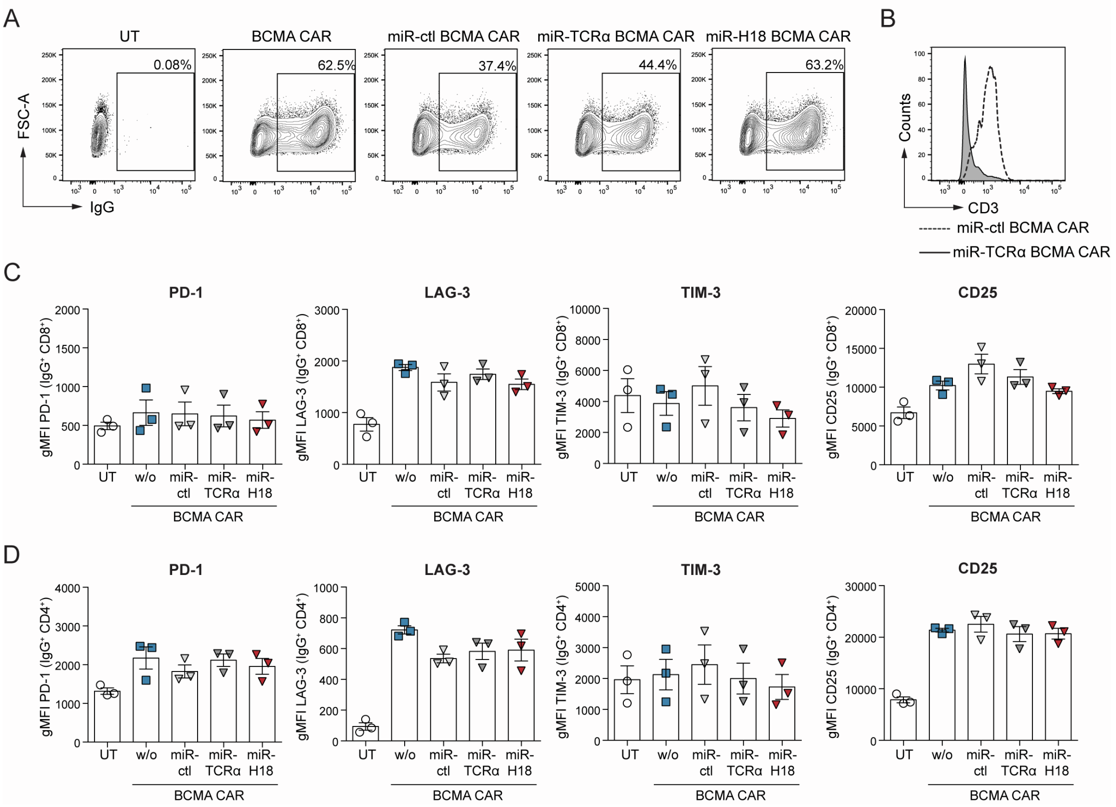

Figure S4: Phenotypic characterization of human BCMA CAR T cells expressing various miRNAs

(A) Retroviral transduction of primary human T cells with BCMA CAR-encoding MP71 vectors expressing various miRNAs directed against TCRα-chain (miR-TCRα), EBAG9 (miR-H18), or without any endogenous target (miR-ctl). Transduction rates are indicated by numbers in the gates.

(B) Efficient miR-TCRα-mediated downregulation of CD3. A representative histogram of flow cytometric analysis of CD3 surface expression on transduced T cells is shown. The miR-ctl served as a control.

(C) Quantification of flow cytometric analysis of key immune markers for T cell function. $CD8^+$ CAR T cells (IgG^+ $CD8^+$) at day 7 after activation were co-stained with anti-PD-1, anti-LAG-3, anti-TIM-3, and anti-CD25 antibodies.

(D) Quantification of flow cytometric analysis of key immune markers for $CD4^+$ CAR T cells at day 7 after activation. C) and D), bars represent mean ± SEM of one experiment with n=3 independent donors per group.

Figure S5

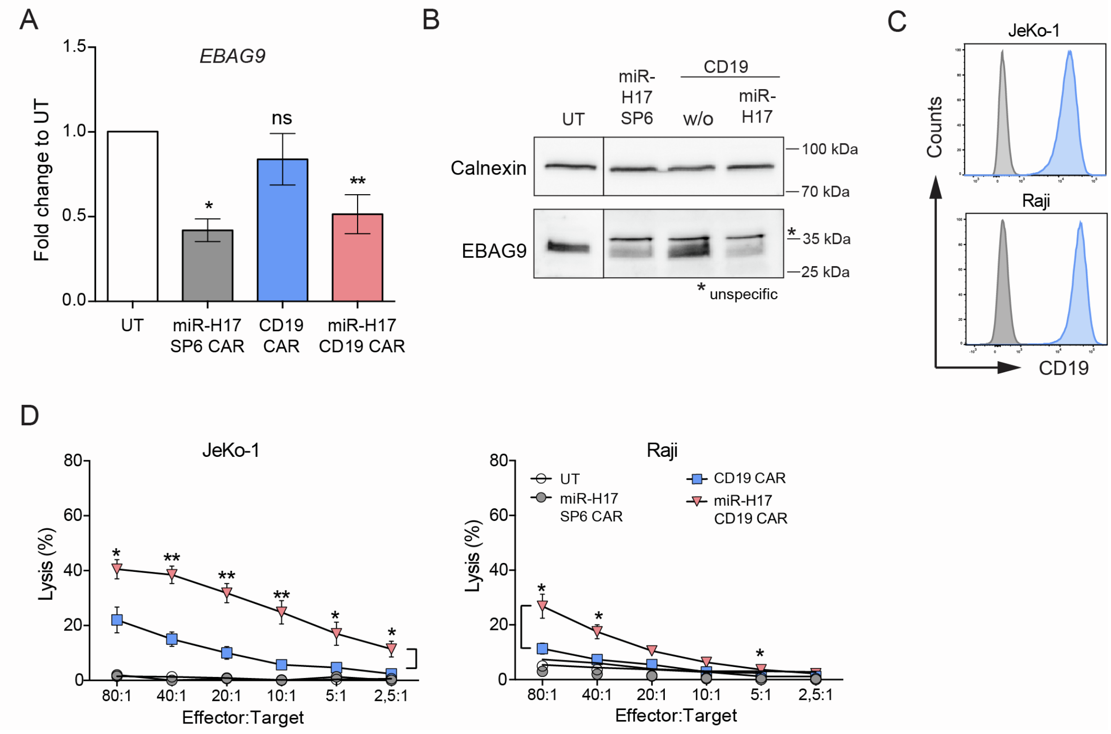

Figure S5: Increasing the cytolytic activity of CAR T cells by silencing EBAG9 is a universally applicable cell biological mechanism

(A) miRNA-mediated reduction of *EBAG9* mRNA-level in human $CD8^+$ T cells by the use of miR-H17. CAR-expressing $CD8^+$ IgG^+ cells were sorted by FACS. Gene expression was determined by qRT-PCR. UT cells were set arbitrarily at 1. Bars represent mean values ± SEM of n=2-4 experiments with n=3 (miR-H17 SP6), n=9 (UT, CD19, miR-H17 CD19) independent donors per group. *p<0.05, **p<0.01; ns, not significant. A one-sample t-test was performed.

(B) Analysis of EBAG9 protein expression in miR-H17 and CAR transduced T cells. CAR-expressing $CD8^+$ IgG^+ T cells were sorted by FACS and analyzed by Western blot. One representative Western blot out of two experiments is shown. Calnexin served as a loading control; * represents an unspecific protein band.

(C) Flow cytometric analysis of CD19 surface expression on the B-NHL cell line JeKo-1 and the B-ALL cell line Raji. A representative histogram for each cell line is shown.

(D) *In vitro* cytotoxicity assays were performed by co-culturing $CD8^+$ CAR T cells for 4 hrs with [^{51}Cr]-labeled CD19-expressing B-NHL cell lines (Jeko-1, Raji) ($5x10^3$ cells each) at the indicated effector to target ratios. Transduction rates of CAR T cells were adjusted to 14% by addition of non-transduced T cells (UT). Data represent mean ± SEM error bars n=3 experiments performed in duplicates with n=3 (miR-H17 SP6) and n=6 (UT, CD19, miR-H17 CD19) different donors per group. *p<0,05, **p<0,01; ns, not significant. A Mann-Whitney U test was employed.

Figure S6

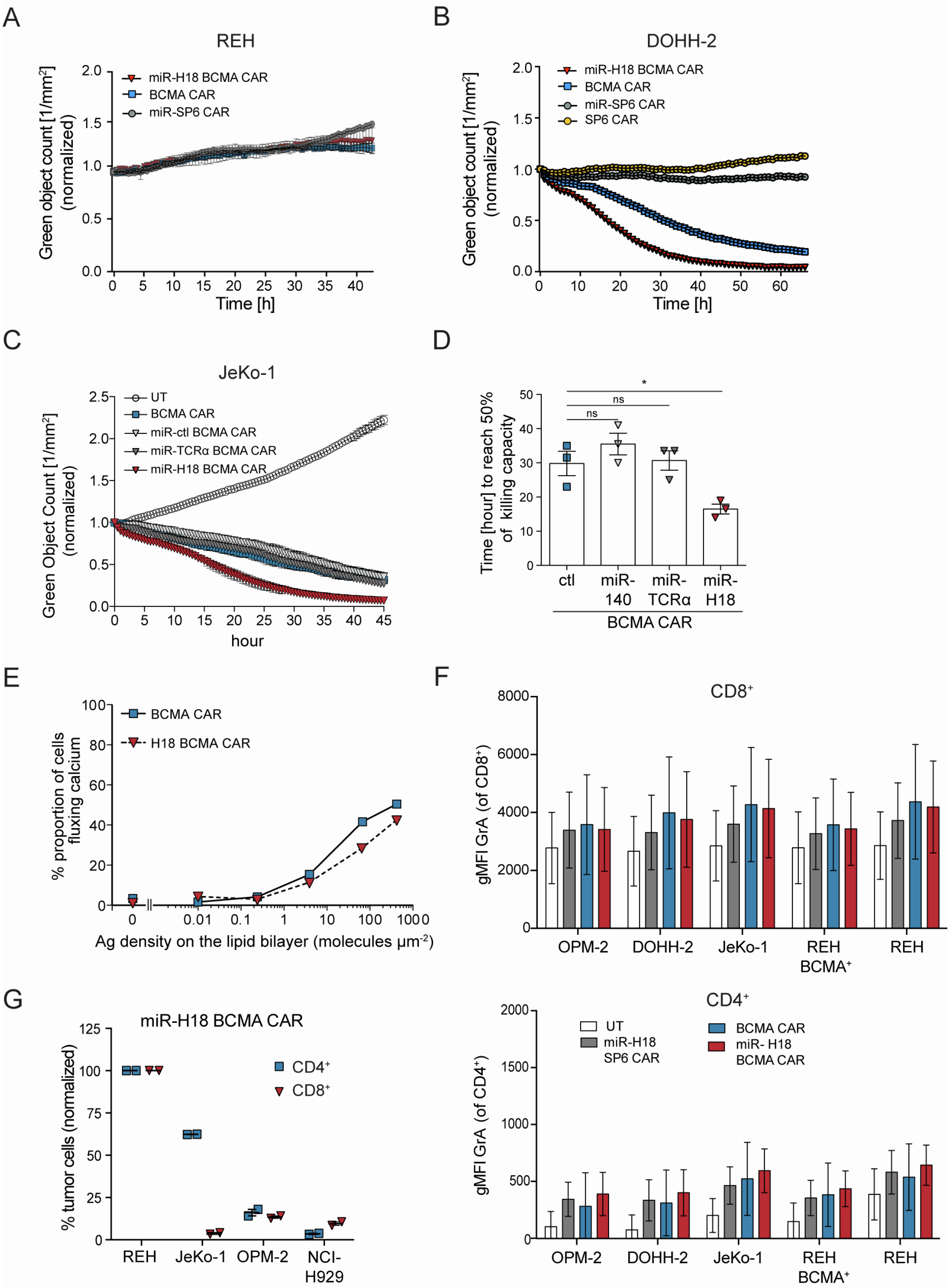

Figure S6: RNAi-mediated increase of CAR T cell cytotoxicity maintains antigen specificity and is selective for EBAG9

(A) The kinetics of CAR T cell cytotoxicity was assessed by analyzing co-cultures of CAR T cells and target cells in an IncuCyte system. As a control, CAR T cells were

co-cultured with BCMA-negative REH cells stably expressing GFP at an effector to target ratio of 5:1 for 42 hrs. GFP fluorescence intensity was normalized for time point 0. Data were measured in triplicates and represent mean ± SEM of n=3 experiments with n=6 (UT, miR-H18 SP6) and n=8 (BCMA CAR, miR-H18 BCMA CAR) independent donors per group.

(B) CAR T cell cytotoxicity was determined as in (A) by analyzing co-cultures of CAR T cells and DOHH-2 target cells. Enriched $CD8^+$ CAR T cells were co-cultured with DOHH-2 cells stably expressing GFP at an effector to target ratio of 1:1 for 66.5 h. Target cell GFP fluorescence intensity was measured every 30 min and normalized to the first time point. The fold change in tumor fluorescence intensity from t = 0 to t = 66.5 h is shown and was measured in triplicates. One representative example of 3 independent donors tested is shown. No differences between CAR T cells transduced with either an SP6 CAR or the miR-H18-modulated SP6 CAR could be observed.

(C) $CD8^+$ CAR T cell cytotoxicity was determined as in (B) by analyzing co-cultures of CAR T cells and JeKo-1 target cells. Data were measured in duplicates and represent mean ± SEM of one experiments with n=3 independent donors per group.

(D) Quantification bar plot of the time to reach 50% cytotoxicity of targeted tumor cell lines in (C). Data are shown as mean ± SEM. A unpaired t-test was applied. *$p<0.05$, ns, not significant.

(E) Fura-2-loaded BCMA and miR-H18 BCMA CAR T cells were stimulated on supported lipid bilayers (SLBs) loaded with indicated numbers of fluorescently labeled BCMA antigen molecules. Proportion of cells fluxing calcium were plotted as a function of antigen density (molecules μm^{-2}). Data for one representative donor out of two is shown (n=2 independent experiments). The number of cells assayed for each data point in the shown plot ranged from n=556 to 1434 (median n=700).

(F) Intracellular granzyme A FACS staining of BCMA CAR T cells. $CD8^+$ and $CD4^+$ CAR T cells were co-cultured with different target cell lines at a 1:1 ratio for 2 hrs. Data are shown as mean ± SEM of n=2 experiments with n=5 independent donors per group.

(G) *In vitro* cytotoxicity of $CD4^+$ and $CD8^+$ miR-H18 BCMA CAR T cells against target cells with variable BCMA surface density. T cells were magnetically separated from total miR-H18 BCMA CAR T cells and co-cultured in a 1:1 ratio with $BCMA^{high}$- (OPM-2, NCI-H929) or $BCMA^{low}$ -expressing (JeKo-1) target cells for 72 hrs. The BCMA-negative cell line REH was used as a control and the results were normalized to remaining REH cells in culture. Remaining tumor cells were quantitated by FACS, and data are represented as dot plots with mean ± SEM of n=2 independent donors.

Figure S7

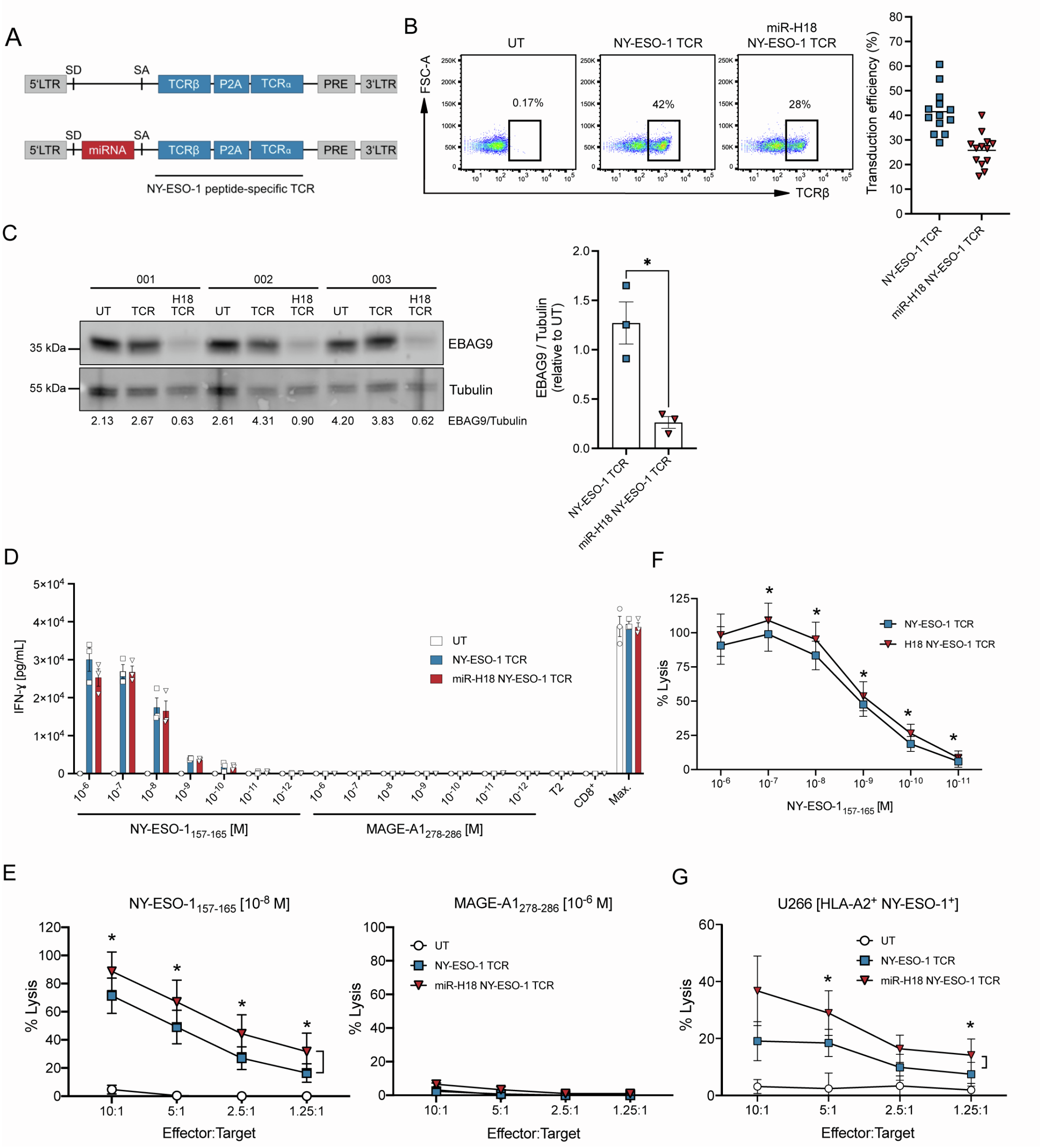

Figure S7: EBAG9 depletion arms CD8^{+} T cells transduced with an NY-ESO-1 TCR with enhanced antigen-specific cytolytic effector functions

A) Schematic depicting ɣ-retroviral constructs (MP71 backbone) encoding an HLA-A*0201-restricted NY-ESO-1 peptide-specific TCR with (miR-H18 NY-ESO-1 TCR, bottom) or without (NY-ESO-1 TCR, top) an intronic miRNA (H18) targeting EBAG9.

B) Representative flow cytometry dot plots (left) and quantitation (right) demonstrating transduction efficiency (%) of enriched $CD8^+$ T cells (>85 % $CD3^+$ $CD8^+$). NY-ESO-1 peptide-specific TCR was detected using an anti-mouse TCRβ antibody targeting the murinized constant region of the TCRβ chain. Depicted populations are $CD3^+CD8^+$ $TCR\beta^+$. Frequencies are indicated as percentages on the gate. UT = untransduced $CD8^+$ T cells. Data represents 13 donors (n=13) assessed in 8 independent experiments.

C) Western blot (left) and densitometry (right) assessing EBAG9 expression of FACS sorted (anti-mouse $TCR\beta^+$) NY-ESO-1 TCR or miR-H18 NY-ESO-1 TCR $CD8^+$ T cells. Densitometry is calculated by dividing signal intensity of EBAG9 expression by the corresponding loading control (Tubulin). Normalized EBAG9 expression in transduced $CD8^+$ T cells from each donor was divided by normalized EBAG9 expression in the corresponding UT control. Data represents 3 donors (n=3). Significance calculated using a paired t-test.

D) Quantitation of IFN-ɣ concentration in cell culture supernatants from 18 h co-cultures of TCR-transduced $CD8^+$ T cells with T2 cells. Target cells were peptide-pulsed with decreasing concentrations of cognate NY-ESO-$1_{157\text{-}165}$, or control MAGE-$A1_{278\text{-}286}$ peptides at the indicated concentrations; effector:target ratio 1:1. Equal proportions of transduced $CD8^+$ T cells from each donor and construct were used. Differences in transduction rates were adjusted using UT $CD8^+$ T cells. All samples were performed in triplicates. Bars represent mean ± SEM of n=3 independent donors per group. Max, stimulation with PMA and ionomycin.

E) Cytolytic capacity of NY-ESO-1 TCR, miR-H18 NY-ESO-1 TCR or untransduced (UT) $CD8^+$ T cells (transduction rate: average n=7, NY-ESO-1 TCR 38%; miR-H18 NY-ESO-1 TCR 26%). Cytotoxicity of NY-ESO-1 TCR-equipped $CD8^+$ T cells was

assessed in co-cultures with T2 cells peptide-pulsed with cognate NY-ESO-$1_{157\text{-}165}$ peptide [10^{-8} M] (left panel), control MAGE-$A1_{278\text{-}286}$ peptide [10^{-6} M] (right panel) at the indicated E:T ratio for 4 hr. Target cell lysis (%) was quantified by enzymatically detection of LDH released. Equal proportions of transduced $CD8^+$ T cells from each donor and construct were used. Differences in transduction rates were adjusted using UT $CD8^+$ T cells from the same donor. All samples were performed in triplicates. Data represents n=7 donors assessed in 4 independent experiments. Statistics calculated using the Wilcoxon matched-pairs signed rank test.

F) Comparison of cytolytic activity of $CD8^+$ T cells transduced with NY-ESO-1 TCR or miR-H18 NY-ESO-1 TCR constructs. Transduced $CD8^+$ T cells (transduction rate: average n=7 donors; NY-ESO-1 TCR 41.2 %; miR-H18 NY-ESO-1 TCR 26.2 %) were co-cultured with T2 cells peptide-pulsed with decreasing concentrations of cognate NY-ESO-$1_{157\text{-}165}$ peptide, E:T of 10:1. Target cell lysis was quantified as in (D). All samples were performed in triplicates. Data represents 7 donors (n=7) assessed in 3 independent experiments. Statistics calculated using the Wilcoxon matched-pairs signed rank test.

G) In vitro cytotoxicity assay of TCR-transduced $CD8^+$ T cells (transduction rate: average n=6 NY-ESO-1 TCR 39.2 %; miR-H18 NY-ESO-1 TCR 23.7 %) at the indicated E:T ratios with the MM cell line U266 (HLA-A*0201^+ and NY-ESO-1^+). Target cell lysis was quantified as in (D). All samples were performed in triplicates. Data represents 6 donors (n=6) assessed in 4 independent experiments. Statistics calculated using the Wilcoxon matched-pairs signed rank test; bars represent mean ± SEM *$p<0.05$.

Figure S8

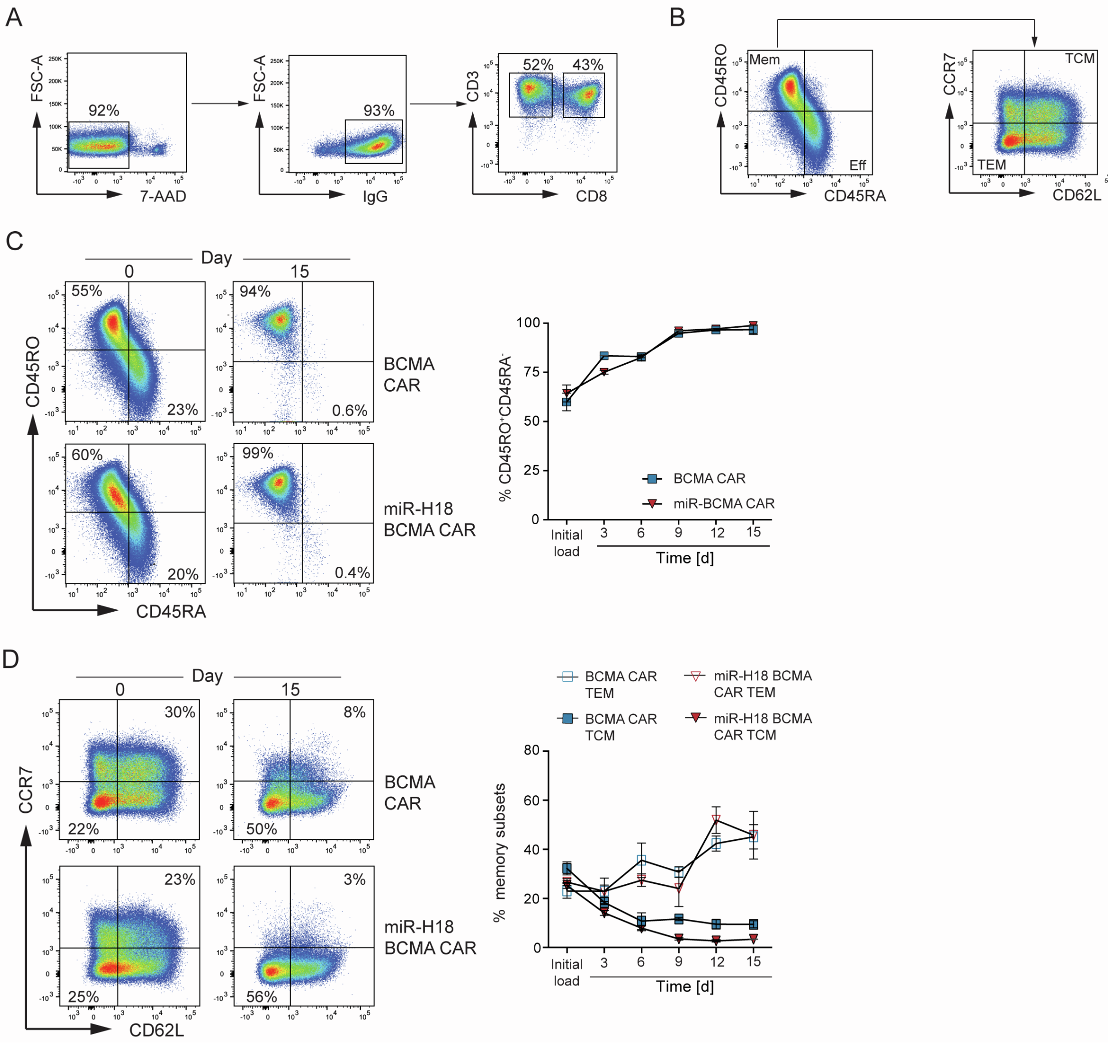

Figure S8: Differentiation of transduced human CAR T cells towards effector memory T cells is not affected by the loss of EBAG9

(A) Gating strategy for flow cytometric analysis of CAR T (IgG^+) cells after each round of co-culture with MM.1S target cells. Frequencies are indicated as numbers on the gates.

(B) In every round of co-cultivation, memory T cell subset composition was analyzed by flow cytometry. Gating strategy for the definition of memory subtypes is shown (TEM: $CD45RO^+$ $CD45RA^-$, $CD62L^-$ $CCR7^-$; TCM: $CD45RO^+$ $CD45RA^-$, $CD62L^+$ $CCR7^+$). Mem, memory T cells; Eff, T effector cells.

(C) Percentages of memory T cells were determined by analysis of CD45RO$^+$ CD45RA$^-$ cells in one experiment with n=2 independent donors per CAR specificity. Mean values ± SEM are plotted for each CAR. Representative dot plots are shown on the left. Frequencies are indicated as percentages on the gate.

(D) Co-staining of CD45RO$^+$ CD45RA$^-$ cells with CD62L and CCR7 was used to distinguish between central and effector memory T cells in one experiment with n=2 independent donors per CAR specificity. Mean values ± SEM are plotted. Representative dot plots are shown on the left. Frequencies are indicated as percentages on the gate.

Figure S9

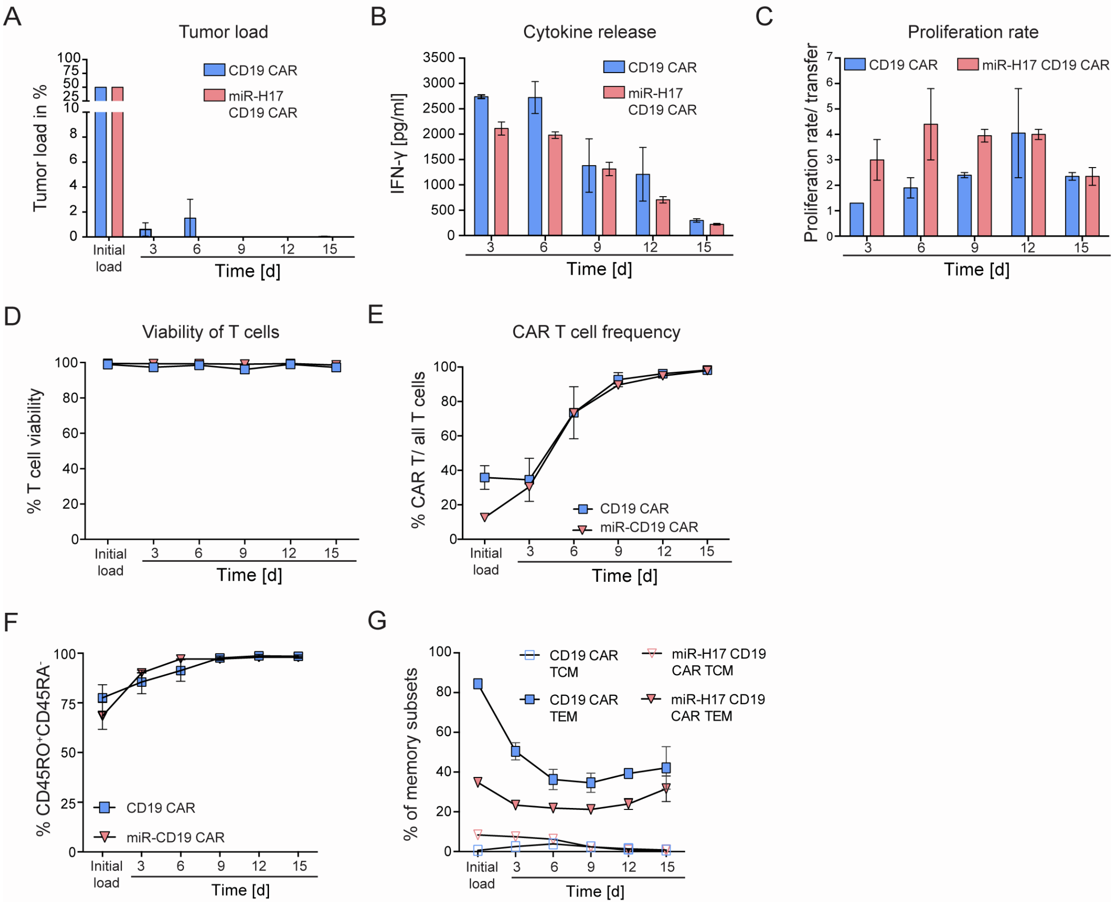

Figure S9: CD19 CAR T cells with silenced EBAG9 maintain their effector functions and proliferative capacity through cycles of repeated antigen exposure

(A)-(G) CD19 CAR T cells and miR-H17 CD19 CAR T cells were co-cultured with JeKo-1 target cells at a 1:1 ratio over a time period of 15 days and five transfer rounds. Every 72 hrs, CAR T cells were transferred to fresh target cells and tumor load (A), IFN-γ secretion (B), proliferation rate (C), CAR T cell viability (D), CAR T cell frequency (E) and memory composition (F, G) were assessed. Data are depicted as mean value ± SEM of n=1 experiment with n=2 independent donors per group.

Figure S10

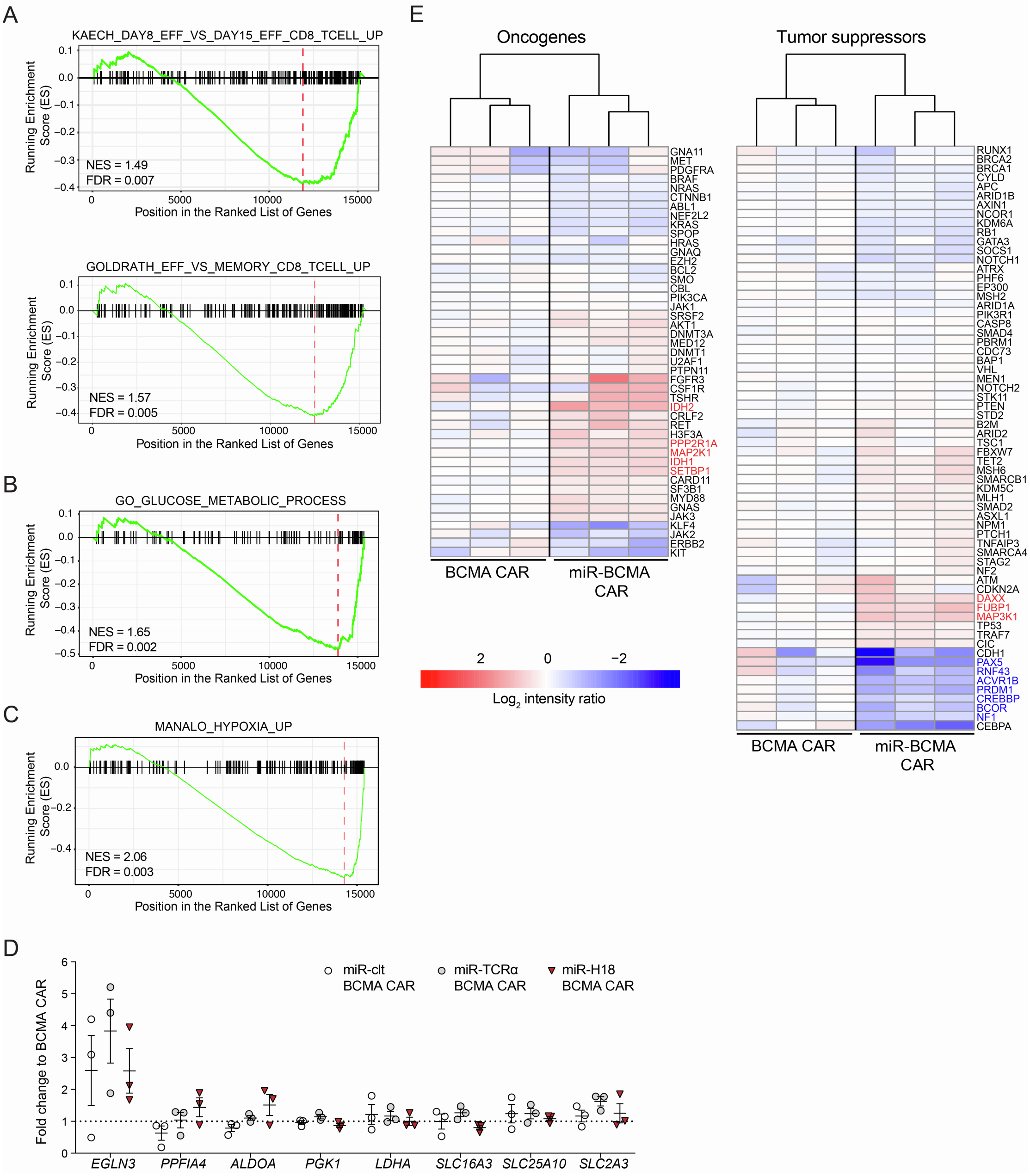

Figure S10: Expression of oncogenes and tumor suppressor genes in BCMA and miR-H18 BCMA CAR T cells

(A) – (C) Representative GSEA results from unbiased functional comparison of BCMA CAR and miR-H18 BCMA CAR transduced T cells using the complete MSigDB (version 6.5). The cutoff of the adjusted *P*-value (for gene lists enrichment significance) was set to 0.2 and 18668 gene lists were tested.

(D) Gene expression analysis of sorted CAR-expressing BCMA-, miR-ctl BCMA-, miR-TCRα BCMA-, and miR-H18 BCMA-CAR T cells at day 10 of culture with supplementation of IL-7 and IL-15. A qRT-PCR assay was applied. BCMA CAR T cells were set arbitrarily at 1, indicated by the dashed horizontal line. Data represent one experiment with n=3 independent donors per group.

(E) Heat maps of oncogenes and tumor suppressor genes that are frequently mutated in various cancer types. Oncogenes or tumor suppressor genes as defined by[1] differentially expressed ($p<0.05$) in miR-H18 BCMA CAR T cells compared to BCMA CAR T cells are marked in red (upregulated) or blue (downregulated). Expression changes are depicted according to the color scale.

Table S1: Exhaustion gene set

Listed are the exhaustion-correlated genes chosen for the transcriptome profiling of the isolated BCMA and miR-H18 BCMA CAR T cells. The genes are grouped and ordered regarding their biological function and differential expression in the miR-H18 BCMA CAR T cell group. The baseMean column shows the average normalized expression (linear scale), lfcShrunk the log2-fold change after shrinkage, wpadj the P value after multiple testing correction, DE the differential expression call (- for downregulated and + for upregulated in the miR-H18 BCMA CAR T cell group, 0 for not altered) and the rank up column the gene's rank when ordered from the signed highest to lowest log2-fold change.

Table S2: Oncogene and tumor suppressor gene set

Listed are oncogenes (top) and tumor suppressor genes (bottom) that are often mutated in various cancer types. The genes were analyzed regarding their differential expression in miRNA-modified BCMA CAR T cells compared to unmodified BCMA CAR T cells. The baseMean column shows the average normalized expression (linear scale), lfcShrunk the log2-fold change after shrinkage, wpadj the P value after multiple testing correction, DE the differential expression call (- for downregulated and + for upregulated in the miR-H18 BCMA CAR T cell group, 0 for not altered) and the rank up column the gene's rank when ordered from the signed highest to lowest log2-fold change.

Supplemental Methods
Reagents Table

REAGENT or RESOURCE	SOURCE	IDENTIFIER
**Antibodies**		
Ultra-LEAF Purified anti-mouse CD3 (clone: OKT3)	BioLegend	Cat # 317326; RRID:AB_11150592
Ultra-LEAF Purified anti-mouse CD28 (clone: CD28.2)	BioLegend	Cat # 102116; RRID:AB_11147170
Ultra-LEAF Purified anti-mouse CD16/32 (clone: 93)	BioLegend	Cat# 101330, RRID:AB_2561482
PE anti-mouse CD4 (clone: GK1.5)	BioLegend	Cat# 100408, RRID:AB_312693
BV421 anti-mouse CD8a (clone: 53-6-7)	BioLegend	Cat# 100738, RRID:AB_11204079
PB anti-mouse CD8a (clone: 53-6-7)	BioLegend	Cat# 100725, RRID:AB_493425
Ultra-LEAF Purified anti-human CD3 (clone: OKT3)	BioLegend	Cat# 300332, RRID:AB_11150396
Ultra-LEAF Purified anti-human CD28 (clone: CD28.2)	BioLegend	Cat# 302934, RRID:AB_11148949
PB anti-human CD3 (clone: HIT3a)	BioLegend	Cat# 300330, RRID:AB_10551436
BV421 anti-human CD4 (clone: OKT4)	BioLegend	Cat# 317434, RRID:AB_2562134
BV421 anti-human CD4 (clone: RPA-T4)	BioLegend	Cat# 300532, RRID:AB_10965645
APC anti-human CD8a (clone: HIT8a)	BioLegend	Cat# 300912, RRID:AB_314116
PE/Cy7 anti-human CD8 (clone: SK1)	BioLegend	Cat# 344712, RRID:AB_2044008
APC anti-human CD19 (clone: HIB19)	BioLegend	02212, RRID:AB_314242
PB anti-human CD45RA (clone: HI100)	BioLegend	Cat# 304117, RRID:AB_493656
PerCP/Cy5.5 anti-human CD45 RO (clone: UCHL1)	BioLegend	Cat# 304221, RRID:AB_1575041
FITC anti-human CD62L (DREG-56)	BioLegend	Cat# 304804, RRID:AB_314464
PE/Cy7 anti-human CD107a/LAMP1 (clone: H4A3)	BD Biosciences	Cat# 561348, RRID:AB_10644018

BV421 anti-human CD138 (clone: MI35)	BioLegend	Cat# 356516, RRID:AB_2562660
PE anti-human CD197/CCR7 (G043H7)	BioLegend	Cat# 353204, RRID:AB_10913813
AF647 anti-human CD223/LAG-3 (clone: 11C3C65)	BioLegend	Cat# 369304, RRID:AB_2566480
APC anti-human CD269/BCMA (clone: 19F2)	BioLegend	Cat# 357506, RRID:AB_2562889
PE anti-human CD279/PD-1 (clone: EH12.2H7)	BioLegend	Cat# 329906, RRID:AB_940483
BV421 anti-human CD366/TIM-3 (clone: F38-2E2)	BioLegend	Cat# 345008, RRID:AB_11218598
PE IgG (polyclonal)	Southern Biotech	Cat # 2040-09
PE anti-human granzyme A (clone: CB9)	BioLegend	Cat# 507206, RRID:AB_315472
FITC anti-mouse TCR β chain (clone: H57-597)	BioLegend	Cat#109205
Calnexin polyclonal antibody	Enzo Life Science	Cat# ADI-SPA-860-D, RRID:AB_2038898
hFAB Rhodamine Anti-Tubulin antibody	BIO-RAD	12004166
StarBright Blue 700 Goat Anti-Rabbit IgG	BIO-RAD	12004162
EBAG9 polyclonal antibody	in-house	2
**Bacterial and Virus Strains**		
E. coli XL1 Blue	Internal stock	N/A
**Biological Samples**		
Human PBMCs, Buffy Coats, Leukapheresis Material	Voluntary healthy donors	N/A
**Chemicals, Peptides, and Recombinant Proteins**		
RPMI-1640 Medium	GIBCO	Cat #11530586
DMEM Medium	GIBCO	Cat #11995073
Fetal Bovine Serum (South Africa)	PAN-Biotech	Cat #P30-1502
Fetal Bovine Serum	GIBCO	Cat #26140079
Penicillin-Streptomycin (100x)	GIBCO	Cat #11548876
Sodium pyruvate (100x)	GIBCO	Cat #12539059
L-glutamine (100x)	GIBCO	Cat #13462629
Minimum essential medium non-essential amino acids (100x)	GIBCO	Cat #11350912

2-Mercaptoethanol	Thermo Fisher Scientific	Cat #11528926
Recombinant Mouse IL-2	PeproTech	Cat #212-12
Recombinant Human IL-2	PeproTech	Cat #200-02
Recombinant Human IL-7	Miltenyi Biotec	Cat #130-095-363
Recombinant Human IL-15	Miltenyi Biotec	Cat #130-095-765
Dynabeads Mouse T-Activator CD3/CD28	Thermo Fisher Scientific	Cat #114.52D
Protamine sulfate	Sigma Aldrich	Cat #1101230005
RetroNectin	TaKaRa	Cat #T202
BioColl	Biochrom	Cat #BS.L 6115
Monensin/Golgi-Stop	BD Bioscience	Cat #554724
Brefeldin A	Sigma Aldrich	Cat #B7651
Ionomycin	Calbiochem	Cat #407952
Phorbol-12-myristate-13-acetate (PMA)	Promega	Cat #V1171
eFluor670	eBioscience	Cat #65-0840-85
Luciferin	Biosynth	Cat #FL08607
[^{51}Cr] sodium chromate	Perkin Elmer	Cat #NEZ030002MC
N^{α}Benzyloxycarbonyl-L-lysine Thiobenzyl Ester	Merck Millipore	Cat #200274
5,5`-Dithio-*bis*-(2-nitrobenzoic acid)	Sigma Aldrich	Cat #D8130
Pierce ECL Western Blotting Substrate	Thermo Fisher Scientific	Cat #32106
Bovine Serum Albumin	Sigma Aldrich	Cat #05470
FastDigest ECoRI	Thermo Fisher Scientific	Cat #FD0274
FastDigest NotI	Thermo Fisher Scientific	Cat #FD0594
Thermosensitive alkaline phosphatase (Fast-AP)	Thermo Fisher Scientific	Cat #EF0651
T4 DNA Ligase	Thermo Fisher Scientific	Cat #15224041
SuperScript III First-Strand Synthesis SuperMix for qRT-PCR	Thermo Fisher Scientific	Cat #11752050
TaqMan Fast Advanced Master Mix	Thermo Fisher Scientific	Cat #4444557
D^{b}-restricted peptide HY antigen: WMHHNMDLI	Biosyntan, Berlin	N/A

Immunodominant peptide IV, SV40 large T antigen VVYDFLKL	JPT Peptide Technologies GmbH, Berlin	N/A
HLA-A2*0201 restricted peptide NY-ESO-1 (157-165): SLLMWITQC	Discovery Peptides, Billingham, UK	N/A
HLA-A2*0201 restricted peptide MAGE-A1 (278-286): KVLEYVIKV	Discovery Peptides, Billingham, UK	N/A
**Critical Commercial Assays**		
TruSeq Stranded mRNA Library Prep Kit	Illumina	Cat #20020594
Easy Sep Human T Cell Isolation Kit	STEMCELL	Cat #17951
CD8 T cell Isolation Kit, human	Miltenyi Biotech	Cat #130-096-495
CD138 MACS Micro Beads	Miltenyi Biotec	Cat #130-051-301
LS columns	Miltenyi Biotec	Cat #130-042-401
Easy Sep PE Positive Selection Kit II	STEMCELL	Cat #17684
FIX&PERM Cell Permeabilization Kit	Molecular Probes	Cat #GAS003
QIAquick Gel Extraction Kit	QIAGEN	Cat #28706
RNeasy Mini Kit	QIAGEN	Cat #74104
CytoTox 96 Non-Radioactive Cytotoxicity Assay	Promega	Cat# G1780
**Deposited Data**		
RNAseq data are deposited at Genbank/ENA; Primary Accession code: PRJEB37843; Secondary Accession code: ERP121180		
**TaqMan Primer**		
Ebag9	Thermo Fisher Scientific	Mm01167189_mH
EBAG9	Thermo Fisher Scientific	Hs00188444_m1
Gapdh	Thermo Fisher Scientific	Mm99999915_g1
GAPDH	Thermo Fisher Scientific	Hs02786624_g1
EGLN3	Thermo Fisher Scientific	Hs00222966_m1
PPFIA4	Thermo Fisher Scientific	Hs00949811_m1
ALDOA	Thermo Fisher Scientific	Hs00605108_g1
PGK1	Thermo Fisher Scientific	Hs00943178_g1
LDHA	Thermo Fisher	Hs01378790_g1

	Scientific	
SLC16A3	Thermo Fisher Scientific	Hs00358829_m1
SLC25A10	Thermo Fisher Scientific	Hs00201730_m1
SLC2A3	Thermo Fisher Scientific	Hs00359840_m1
SDHA	Thermo Fisher Scientific	Hs07291714_mH
**Experimental Models: Cell Lines**		
NCI-H929	DSMZ, Braunschweig	Cat# ACC-163, RRID:CVCL_1600
OPM-2	DSMZ, Braunschweig	Cat# ACC-50, RRID:CVCL_1625
DOHH-2	DSMZ, Braunschweig	Cat# ACC-47, RRID:CVCL_1179
JeKo-1	DSMZ, Braunschweig	Cat# ACC-553, RRID:CVCL_1865
Jurkat J76	DSMZ, Braunschweig	Cat# ACC-282, RRID:CVCL_0065
REH	Dr. Stephan Mathas, MDC, Berlin	N/A
MM.1S luc eGFP	(Oden et al., 2015) [3]	N/A
HEK-293T	Quantum Biotechnologies	N/A
293VecGalV	BioVec Pharma	[4]
PlatE	Dr. Wolfgang Uckert, MDC	[5]
Co16.113	Dr. Gerald Willimsky, Charité, Berlin	N/A
B3Z	Dr. Wolfgang Uckert, MDC	[6]
Raji	(Oden et al., 2015) [3]	ATCC #CCL-86
U-266	DSMZ, Braunschweig	Cat# ACC-9
T2	Dr. Simone Rhein, MDC, Berlin	Cat# ACC-598
**Experimental Models: Organisms/Strains**		
NOD.Cg-Prkdcscid Il2rg tm1 Wji/Szj (NSG) mice	The Jackson Laboratories	Cat# JAX:005557, RRID:IMSR_JAX:005557
C57BL/6N mice	Charles River	N/A

Rag2$^{-/-}$ mice	Charles River	N/A
Ebag9 -/-	7	N/A
**Recombinant DNA**		
pALF-10A1GaV	Internal stock	N/A
pcDNA3.1-MLV gag/pol	Dr. Wolfgang Uckert, MDC	N/A
MP71_GFP	Dr. Wolfgang Uckert, MDC	8
MP71_miR-ctl_GFP	This manuscript	N/A
MP71_miR-M1-M5_GFP	This manuscript	N/A
MP71_miR-H16-H19_GFP	This manuscript	N/A
MP71_BCMA CAR	9	N/A
MP71_miR-ctl_BCMA CAR	This manuscript	N/A
MP71_miR-TCRα_BCMA CAR	This manuscript	N/A
MP71_miR-H18_BCMA CAR	This manuscript	N/A
MP71_CD19 CAR	10	N/A
MP71_miR-H17_CD19 CAR	This manuscript	N/A
MP71_SP6 CAR	9	N/A
MP71_miR-H17/18_SP6 CAR	This manuscript	N/A
MP71_NY-ESO-1TCR	This manuscript	N/A
MP71_miR-H18 NY-ESO-1 TCR	This manuscript	N/A
**Software and Algorithms**		
FlowJo v. 10.0.8 software	Tree Star	https://www.flowjo.com
Prism version 6.0, 9.0	Graphpad Software Inc.	https://www.graphpad.com
R version 3.6.1	R Core Team	https://www.R-project.org/.
Bioconductor 3.9	Bioconductor	https:/bioconductor.org
Salmon version 0.14.1	11	https:// github.com/COMBINE-lab/salmon
Tximeta version 1.5.6	12	https:/bioconductor.org
DESeq version 1.24.0	13	https:/bioconductor.org

clusterProfiler version 3.12.0	14	
Living Image software version 4.5	Caliper Life Sciences	https:/www.perkinelmer.com

Supplemental references

1. Vogelstein, B, Papadopoulos, N, Velculescu, VE, Zhou, S, Diaz, LA, Jr., and Kinzler, KW (2013). Cancer genome landscapes. *Science* **339**: 1546-1558.
2. Engelsberg, A, Hermosilla, R, Karsten, U, Schulein, R, Dorken, B, and Rehm, A (2003). The Golgi protein RCAS1 controls cell surface expression of tumor-associated O-linked glycan antigens. *J Biol Chem* **278**: 22998-23007.
3. Oden, F, Marino, SF, Brand, J, Scheu, S, Kriegel, C, Olal, D, *et al.* (2015). Potent anti-tumor response by targeting B cell maturation antigen (BCMA) in a mouse model of multiple myeloma. *Mol Oncol* **9**: 1348-1358.
4. Ghani, K, Wang, X, de Campos-Lima, PO, Olszewska, M, Kamen, A, Riviere, I, *et al.* (2009). Efficient human hematopoietic cell transduction using RD114- and GALV-pseudotyped retroviral vectors produced in suspension and serum-free media. *Hum Gene Ther* **20**: 966-974.
5. Morita, S, Kojima, T, and Kitamura, T (2000). Plat-E: an efficient and stable system for transient packaging of retroviruses. *Gene Ther* **7**: 1063-1066.
6. Karttunen, J, Sanderson, S, and Shastri, N (1992). Detection of rare antigen-presenting cells by the lacZ T-cell activation assay suggests an expression cloning strategy for T-cell antigens. *Proc Natl Acad Sci U S A* **89**: 6020-6024.
7. Ruder, C, Hopken, UE, Wolf, J, Mittrucker, HW, Engels, B, Erdmann, B, *et al.* (2009). The tumor-associated antigen EBAG9 negatively regulates the cytolytic capacity of mouse CD8+ T cells. *J Clin Invest* **119**: 2184-2203.
8. Engels, B, Cam, H, Schuler, T, Indraccolo, S, Gladow, M, Baum, C, *et al.* (2003). Retroviral vectors for high-level transgene expression in T lymphocytes. *Hum Gene Ther* **14**: 1155-1168.
9. Bluhm, J, Kieback, E, Marino, SF, Oden, F, Westermann, J, Chmielewski, M, *et al.* (2018). CAR T Cells with Enhanced Sensitivity to B Cell Maturation Antigen for the Targeting of B Cell Non-Hodgkin's Lymphoma and Multiple Myeloma. *Mol Ther* **26**: 1906-1920.
10. Lamers, CH, Sleijfer, S, Vulto, AG, Kruit, WH, Kliffen, M, Debets, R, *et al.* (2006). Treatment of metastatic renal cell carcinoma with autologous T-lymphocytes genetically retargeted against carbonic anhydrase IX: first clinical experience. *J Clin Oncol* **24**: e20-22.
11. Patro, R, Duggal, G, Love, MI, Irizarry, RA, and Kingsford, C (2017). Salmon provides fast and bias-aware quantification of transcript expression. *Nat Methods* **14**: 417-419.
12. Love, MI, Soneson, C, Hickey, PF, Johnson, LK, Pierce, NT, Shepherd, L, *et al.* (2020). Tximeta: Reference sequence checksums for provenance identification in RNA-seq. *PLoS Comput Biol* **16**: e1007664.
13. Ignatiadis, N, Klaus, B, Zaugg, JB, and Huber, W (2016). Data-driven hypothesis weighting increases detection power in genome-scale multiple testing. *Nat Methods* **13**: 577-580.

14. Yu, G, Wang, LG, Han, Y, and He, QY (2012). clusterProfiler: an R package for comparing biological themes among gene clusters. *OMICS* **16**: 284-287.
