# Supplementary material for: EBAG9 silencing exerts an immune checkpoint function without aggravating adverse effects
Source: Mol Ther. 2022 Jul 12;30(11):3358–78. doi: 10.1016/j.ymthe.2022.07.009 (PMC9637585; doi:10.1016/j.ymthe.2022.07.009)
Supplement: Document S2. Article plus supplemental information [file mmc4.pdf]

Molecular Therapy
Original Article

EBAG9 silencing exerts an immune checkpoint function without aggravating adverse effects

Anthea Wirges,[1] Mario Bunse,[2] Jara J. Joedicke,[1] Eric Blanc,[3] Venugopal Gudipati,[4] Michael W. Moles,[1] Hiroshi Shiku,[5] Dieter Beule,[3] Johannes B. Huppa,[4] Uta E. Höpken,[2] and Armin Rehm[1]

[1]Translational Tumorimmunology, Max-Delbrück-Center for Molecular Medicine, 13125 Berlin, Germany; [2]Microenvironmental Regulation in Autoimmunity and Cancer, Max-Delbrück-Center for Molecular Medicine, 13125 Berlin, Germany; [3]Core Unit Bioinformatics, Berlin Institute of Health, 10117 Berlin, Germany; [4]Medical University of Vienna, Center for Pathophysiology, Infectiology and Immunology, Institute for Hygiene and Applied Immunology, 1090 Vienna, Austria; [5]Department of Personalized Cancer Immunotherapy, Mie University Graduate School of Medicine, Tsu city, Mie, 514-8507, Japan

Chimeric antigen receptor (CAR) T cells have revolutionized treatment of B cell malignancies. However, enhancing the efficacy of engineered T cells without compromising their safety is warranted. The estrogen receptor-binding fragment-associated antigen 9 (EBAG9) inhibits release of cytolytic enzymes from cytotoxic T lymphocytes. Here, we examined the potency of EBAG9 silencing for the improvement of adoptive T cell therapy. MicroRNA (miRNA)-mediated EBAG9 downregulation in transplanted cytolytic CD8+ T cells (CTLs) from immunized mice improved their cytolytic competence in a tumor model. In tolerant female recipient mice that received organ transplants, a minor histocompatibility antigen was turned into a rejection antigen by *Ebag9* deletion, indicating an immune checkpoint function for EBAG9. Considerably fewer EBAG9-silenced human CAR T cells were needed for tumor growth control in a xenotransplantation model. Transcriptome profiling did not reveal additional risks regarding genotoxicity or aberrant differentiation. A single-step retrovirus transduction process links CAR or TCR expression with miRNA-mediated EBAG9 downregulation. Despite higher cytolytic efficacy, release of cytokines associated with cytokine release syndrome remains unaffected. Collectively, EBAG9 silencing enhances effector capacity of TCR- and CAR-engineered T cells, results in improved tumor eradication, facilitates efficient manufacturing, and decreases the therapeutic dose.

INTRODUCTION

Adoptive T cell therapy (ATT) with engineered chimeric antigen receptor (CAR) T cells has demonstrated efficacy for the treatment of hematologic malignancies. Pronounced clinical responses have been achieved in CD19+ large B cell lymphoma, acute lymphoblastic leukemia (ALL), and chronic lymphocytic leukemia (CLL).[1–3] Despite high complete response rates and durable remissions, relapses occur in a large fraction of patients.[4] Resistance forms in ALL include loss or downregulation of CD19.[5] Results of early clinical trials indicate activity of B cell maturation antigen (BCMA) CAR T cells in multiple myeloma (MM). Although initial response rates amounted to 85% in BCMA CAR-treated MM patients, progression-free survival in these trials was less than 1 year.[6–8] Together, treatment options for post-CAR relapse are limited and render it challenging to achieve complete responses, let alone a cure. In efforts to meet the medical need for more efficacious T cell therapies, multi-dimensional challenges have been identified.[9] Previous T cell-engineering efforts to boost therapy outcomes have been mostly aimed at improving T cell trafficking, tumor recognition, and avoidance of exhaustion.[10]

Cytolytic CD8+ T cells (CTLs) have become the central focus of cellular cancer therapeutics.[11] CTLs destroy tumors by secreting pro-inflammatory cytokines such as interferon gamma (IFN-γ) and tumor necrosis factor alpha (TNF-α), as well as cytotoxic granules containing perforin and granzymes.[12] However, tumor-specific CD8+ T cell activity often falls short due to the immunosuppressive microenvironment, low tumor immunogenicity, or T cell intrinsic inhibitors.[13–15] Extended *ex vivo* culturing times, which are routine for raising therapeutic quantities, do negatively impinge not only on the life span of T cells but also their functional response to antigenic challenge.[16] Any improvement in T cell longevity and fitness are hence likely to promote anti-tumor efficacy, which results from diverse parameters such as the number of surface-expressed antigen receptors, the density of the cognate antigen on the target cell, and the affinity of receptors for their nominal antigen.[9] Other contributing factors to T cell fitness include transcriptional maturation, cytokine signaling, and co-stimulation.[17,18] Alternatively, the secretory pathway is pivotal for CTL effector function as it ensures both availability and triggered release of cytolytic granules for efficient target cell killing. The cytolytic efficacy of CTLs depends on the synthesis and storage of effector molecules, intracellular vesicle transport, as well as the maturation and secretion competence of secretory lysosomes.[19] The estrogen receptor-binding fragment-associated antigen 9 (EBAG9) is a negative regulator of the Ca2+-dependent secretion of effector molecules.[20,21] Genetic deletion of *Ebag9* in mice enhances the release of the lytic granule content from secretory lysosomes in CD8+ T cells.

Received 8 December 2021; accepted 9 July 2022;
https://doi.org/10.1016/j.ymthe.2022.07.009.

Correspondence: Armin Rehm, Translational Tumorimmunology, Max-Delbrück-Center for Molecular Medicine, 13125 Berlin, Germany.
E-mail: arehm@mdc-berlin.de

 © 2022 The Author(s).
This is an open access article under the CC BY license (http://creativecommons.org/licenses/by/4.0/).

Mechanistically, EBAG9 interacts with the γ2-subunit of adaptor protein complex (AP)-1 and inhibits AP-1 activity-mediated clathrin-coated vesicle formation. Furthermore, EBAG9 is an interaction partner of Snapin and BLOS2, which are subunits of the lysosome-related organelles complex-1 (BLOC-1). BLOC-1 regulates protein sorting from the endosome to the secretory lysosome. Thus, EBAG9 inhibits vesicle transfer from the *trans*-Golgi network to the secretory lysosomes. Consequently, loss of EBAG9 endows CD8+ T cells with increased target cell lysis, without perturbing immune homeostasis.[20]

In the present study, we evaluated the contribution of EBAG9 downregulation to the performance of murine T cells and human CAR T cells in ATT. MicroRNA (miRNA)-mediated silencing of *Ebag9* augmented the cytolytic activity of murine CD8+ T cells and human CAR T cells against hematopoietic tumors, both *in vitro* and *in vivo*. Tumor control required substantially fewer effector CAR T cells. Transcriptome profiling ruled out adverse effects on T cell exhaustion and differentiation. Furthermore, the miRNA-approach had a similar genotoxic risk profile to conventional retroviral transductions of mature T cells. Importantly, despite amplified granzyme A secretion, inflammatory cytokines associated with cytokine release syndrome (CRS) remained unaffected by EBAG9 silencing.

RESULTS

Enhanced *in vivo* cytotoxicity of engineered mouse CTLs with silenced EBAG9

Previous work of ours identified EBAG9 as a negative regulator of the cytotoxic activity of T cells.[20] Furthermore, we could demonstrate that a loss of EBAG9 increased the pool of memory CD8+ T cells in mice.[22] Thus, deleting EBAG9 might serve as an attractive strategy to increase the cytolytic competence of engineered T cells for ATT. To further validate the relevance of EBAG9 as a potential target of interference, we challenged *Ebag9*$^{-/-}$ mice with the minor histocompatibility (miHag) HY antigen located on the Y chromosome. Female HY wild-type (WT) and *Ebag9*$^{-/-}$ mice were immunized with HY+ male splenocytes and challenged with male and female splenocytes in an *in vivo* killing assay. Elimination of male splenocytes in female *Ebag9*$^{-/-}$ mice was twice as pronounced compared with that observed in WT recipients (Figure S1A).

Furthermore, we investigated an allotransplantation setting where HY was tolerated when hearts from male donors (HY+) were transplanted into female (HY−) WT recipient mice. In contrast, in female *Ebag9*$^{-/-}$ recipient mice organ rejection occurred already at day 12 with accelerated kinetics (Figure S1B). Since miHag specific alloresponses after donor lymphocyte infusions are of substantial clinical relevance in graft-versus-leukemia effects,[23] we conclude that EBAG9 defines a crucial checkpoint for the cytolytic capacity of CD8+ T cells and may hence be suitable for therapeutic exploitation.

To take advantage of the inhibitory function of EBAG9 in immunotherapy, we opted for a retroviral platform for the generation of T cells expressing a transgene and a redirected miRNA.[24] Five miRNAs directed against different target sites in the open reading frame (ORF) of the mouse *Ebag9* gene (miR-M1 to miR-M5) were generated by exchanging the guide and passenger strand of mouse miR-155. Then the miRNAs were introduced into the 5′ intron of a GFP-encoding retroviral MP71 vector (Figure 1A). A non-targeting miRNA (miR-ctl) served as a negative control. Expression of all five miRNAs in transduced murine B3Z cells reduced EBAG9 protein levels, with the greatest effect seen for miR-M1 and miR-M2 (Figure S2A). In transduced primary mouse T cells, miR-M1 reduced *Ebag9* mRNA levels by 86% compared with 70% for miR-M2. Both miRNAs virtually depleted the cells of EBAG9 protein (Figures 1B and 1C). Introduction of miRNAs into the MP71 vector also marginally affected transduction rates (68% versus 54%) and decreased GFP levels (geometric mean fluorescence intensity, gMFI) to 35%–50% of the parental vector (Figures S2B and S2C). The miR-ctl exhibited a similar degree of GFP reduction as the miR-M1 and miR-M2 targeted at EBAG9. Collectively, the overall yields of engineered T cells with the desired phenotype were sufficient for studying the clinical effects of EBAG9 silencing in ATT.

To assess the *in vivo* cytotoxicity of miRNA-modified CTLs, bulk T cells were isolated from SV40-large T antigen (TAg)-immunized C57BL/6 mice and transduced with the miRNA-modified or parental MP71-GFP vector. After transfer of sorted GFP+ T cells into immunodeficient *Rag2*$^{-/-}$ mice, the recipients were also immunized with TAg and challenged on day 19 or 20 with equal numbers of TAg peptide IV-loaded and non-loaded splenocytes (Figure 1D). Recipients that had received *Ebag9*-specific miR-M1 T cells showed a 2-fold higher antigen-specific killing rate compared with the control group (Figure 1E). No differences in the engraftment and subtype composition of the transferred T cells between the groups were detected (Figures S2D and S2E). Collectively, EBAG9 silencing enhances the cytolytic capacity of adoptively transferred CTLs against the TAg neoantigen in the context of polyclonal TAg-specific T cells.

Increased release of granzyme A in human CAR T cells after miRNA-mediated silencing of *EBAG9*

To explore the immunotherapeutic potential of miRNA-mediated *EBAG9* silencing in human T cells, a single-step gene engineering approach was developed. As for the murine CTL model, retroviral miRNA vectors targeting human *EBAG9* were designed and tested in transduced Jurkat T cells (Figures S3A–S3C). For the use of miR-H17 and miR-H18, knockdown efficiencies for *EBAG9* mRNA and protein of at least 80% were achieved and, thus, these miRNAs were selected for subsequent applications (Figures S3B and S3C). The GFP transgene of the miR-H18 vector was exchanged for a high-affinity second-generation BCMA CAR that mediates cytotoxic activity against BCMA-expressing human MM, and B non-Hodgkin's lymphoma (B-NHL) cells[25] (Figure 2A). The negative control, SP6 CAR, which is not reactive to any naturally occurring ligand, was similarly combined with the miR-H18 vector. MiR-H18 decreased EBAG9 protein and mRNA levels up to 80% in transduced human T cells that were sorted for CAR expression (Figures 2B and 2C). We also observed that BCMA CAR surface levels were reduced by half in miR-H18-transduced T cells compared with control (median gMFI BCMA CAR, 13.600; median gMFI miR-H18 BCMA CAR, 6.626) (Figures 2D and S3D).

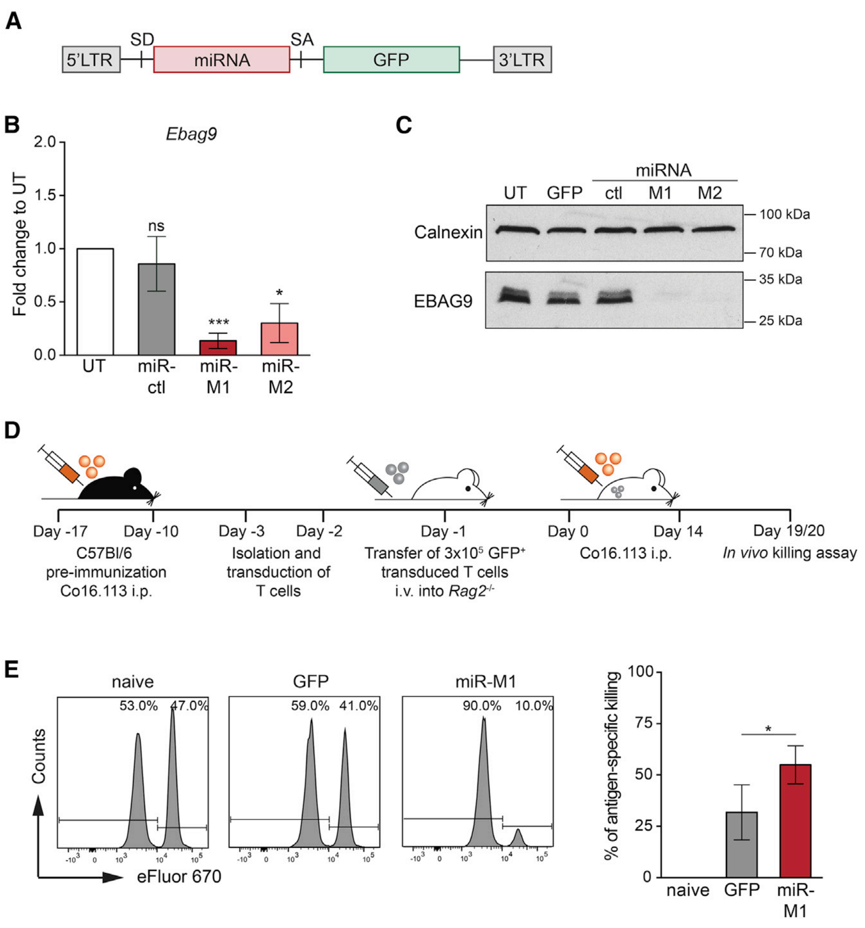

Figure 1. RNAi-mediated silencing of EBAG9 amplifies antigen-specific *in vivo* cytotoxic activity of murine T cells

(A) Design of the retroviral MP71 construct encoding for an intronic *Ebag9*-specific miRNA and a GFP transgene. LTR, long terminal repeat; SD, splice donor; SA, splice acceptor; PRE, post-transcriptional regulatory element. (B) RNAi-mediated reduction of *Ebag9* mRNA. Mouse splenocytes were transduced with MP71-GFP vectors encoding either *Ebag9*-specific miRNAs (miR-M1 and -M2) or a control miRNA (mir-ctl). mRNA expression was analyzed by RT-qPCR in GFP-sorted T cells. Untransduced (UT) cells were set arbitrarily at 1. Bars represent mean ± SEM of n = 3 experiments with n = 3 samples per group. A one-sample t test was applied. *p < 0.05, ***p < 0.001; ns, not significant. (C) RNAi-mediated reduction of EBAG9 protein level. Transduced GFP+ T cells were sorted by FACS and analyzed by western blot. One representative western blot out of two experiments is shown. Calnexin served as a loading control. (D) Schematics of the *in vivo* killing assay. (E) Bulk T cells of TAg-antigen immunized mice were transduced with GFP or miR-M1 retroviruses. Transduced cells were transferred into *Rag2*$^{-/-}$ mice, followed by re-immunization. At day 19, animals were challenged with a 1:1 mixture of non-loaded and peptide-loaded splenocytes labeled with different concentrations of eFluor-670 (low for non-loaded, high for peptide-loaded splenocytes). The ratio of both populations was determined by flow cytometry 16 h later and is expressed as specific killing as a percentage. Histograms show representative examples per group. Percentages of non-loaded and peptide-loaded fractions are indicated. Quantification bar plot of flow cytometry is shown. Bars represent mean ± SEM of n = 5 experiments with n = 5 (naive), n = 10 (GFP), and n = 15 (miR-141) mice per group. A Mann-Whitney U test was applied. *p < 0.05. See also Figures S1 and S2.

To investigate whether the reduced transgene expression is a consequence of the vector composition or the transgenic miRNA expression itself, we performed a double-transduction experiment with Jurkat cells. GFP transgene expression was only affected if the miRNA was encoded by the same vector as the transgene, but not if the miRNA was expressed from an independent vector (Figure S3E). This indicated a feature of polycistronic transcripts combining intronic miRNA and protein-encoding genes. When the splicing efficiency of the intron is not high enough to rescue all transcripts for translation that were cleaved by the Drosha-containing microprocessor complex, then the level of transgenic protein expression drops.[24]

Next, CD8+ T cells from healthy donors were transduced with miR-H18 BCMA CAR or control vectors. Given the differences in observed transduction efficiencies, samples were adjusted to 20%–30% by adding non-transduced (UT) T cells. The amount of granzyme A released from BCMA CAR-transduced T cells was similar to those of UT controls (mean 34.7%). In contrast, T cells transduced with the miR-H18 BCMA CAR and miR-H18 SP6 CAR construct released about 2-fold higher amounts of granzyme A (mean 58.3%) (Figure 2E). BCMA CAR T cells that either contained a non-targeting miRNA (miR-ctl) or a T cell receptor (TCR)α chain-targeting miRNA (miR-TCRα) released granzyme A to the same extent as UT and BCMA-only CAR T cells, confirming the specificity of the miRNA-mediated *EBAG9*-knockdown strategy (Figure S3F).

Furthermore, CD8+ CAR T cells were co-cultured with BCMA^{high}-expressing MM (OPM-2), BCMA^{low}-expressing B-NHL (DOHH-2, JeKo-1), and with BCMA-negative control Jurkat cells (Figure S3G). All BCMA-expressing cell lines activated BCMA CAR T cells, resulting in secretion of the effector cytokines IFN-γ, TNF-α, and interleukin (IL)-2. No significant differences in the release of pro-inflammatory cytokines were observed after EBAG9 downregulation. Control miR-H18 SP6 CAR T cells released no cytokines, and negative control Jurkat cells did not activate CAR T cells at all (Figure 2F).

Adoptive transfer of T cells modified with high-affinity single-chain variable fragment (scFv) CARs often results in severe toxicities in clinical trials.[26] Moreover, strong T cell activation is a driver for exhaustion.[27] Here, we asked the question whether EBAG9 knockdown alters

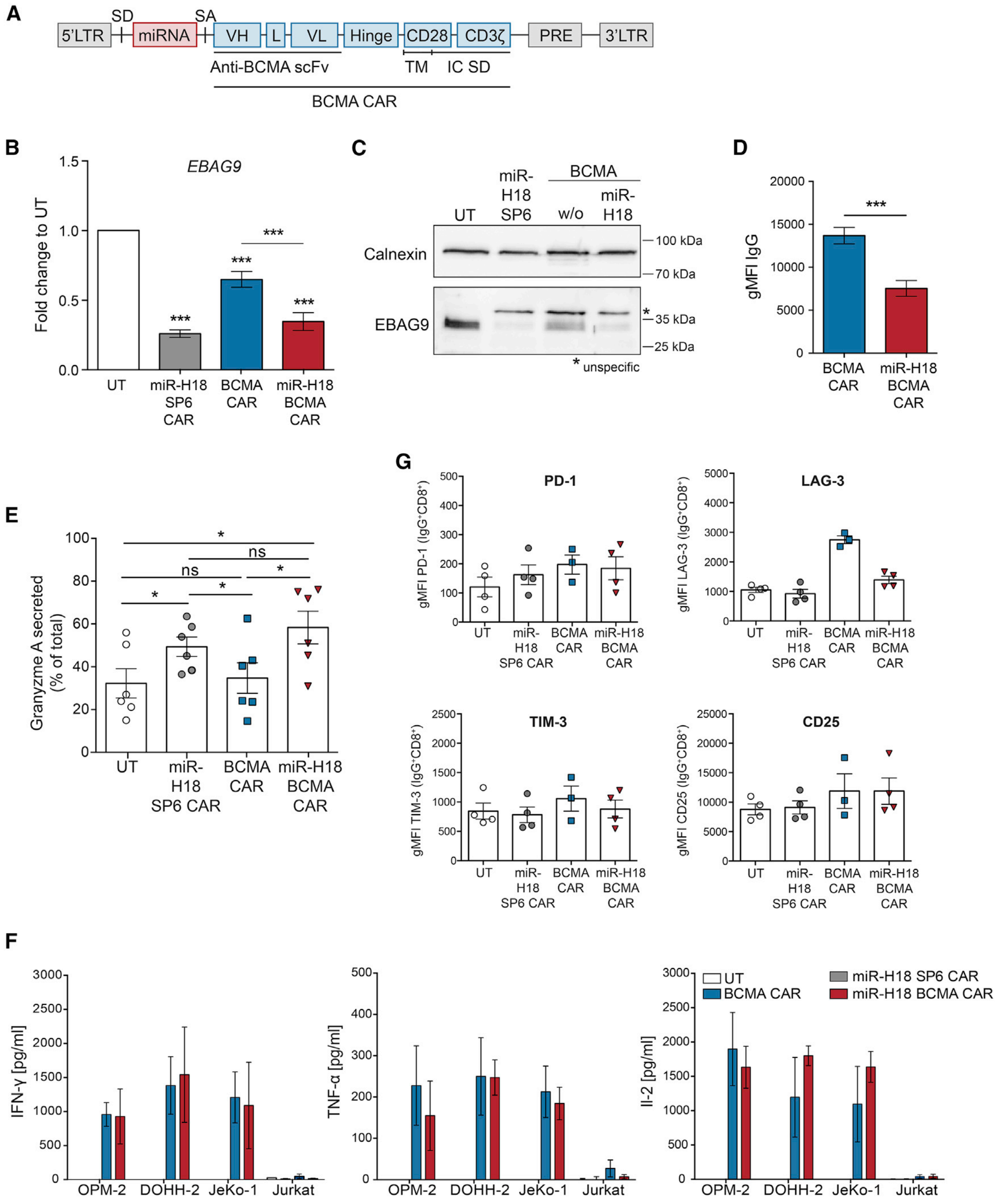

Figure 2. Immunological characterization of human CAR T cells with silenced EBAG9 expression

(A) Design of the retroviral MP71 construct encoding for an intronic *EBAG9*-specific miRNA and the BCMA CAR. LTR, long terminal repeat; SD, splice donor; SA, splice acceptor; VH, heavy chain; L, linker; VL, light chain; scFv, single-chain variable fragment; TM, transmembrane domain; IC SD, intracellular signaling domain; PRE, post-transcriptional regulatory element. (B) RNAi-mediated reduction of *EBAG9* mRNA-level in sorted CD8+ IgG+ cells. Gene expression was determined by qRT-PCR. UT cells were set arbitrarily at 1. Bars represent mean ± SEM of n = 2–5 experiments with n = 4 (UT, miR-H18 SP6 CAR) and n = 10 (BCMA CAR, miR-H18 BCMA CAR) independent donors per group. A one-sample t test was applied. ***p < 0.001. (C) Western blot of EBAG9 protein expression in sorted CD8+ IgG+ cells. One representative immunoblot out of two experiments is shown. Calnexin served as a loading control. (D) Quantification of BCMA CAR surface density (gMFI) by flow cytometry analysis is shown. Bars represent mean ± SEM of n = 5 experiments with n = 14 independent donors per group. A Mann-Whitney U test was applied; ***p < 0.001. (E) Granzyme A release induced by re-stimulation of CD8+ CAR T cells with anti-human CD3 and CD28 antibodies for 4 h. Enzymatic activities in the supernatant were measured in triplicates. Values show the release in percentages relative to the total content. Bars represent mean ± SEM of n = 4 experiments with n = 6 independent donors per group. A Wilcoxon matched-pairs

(legend continued on next page)

the T cell phenotype in culture. At day 7, CD8+ and CD4+ CAR T cells that were cultured with IL-2 showed similar surface expression of the immune checkpoint or intrinsic activation markers PD-1, TIM-3, and CD25 for all groups, independently of experimental miRNA expression. For LAG-3, higher surface levels were obtained in CD8+ and CD4+ BCMA CAR T cells without miR-H18 (Figures 2G and S3H). To examine if altered LAG-3 expression resulted from a general activation of the RNAi pathway or from a miR-H18 sequence-specific effect, we analyzed miR-ctl and miRTCRα-containing BCMA CAR T cells (Figures S4A and S4B). CD8+ and CD4+ BCMA CAR T cells of all groups exhibited a very similar expression of PD-1, TIM-3, and CD25. LAG-3 surface expression was modestly reduced compared with that of BCMA CAR control cells, but this decrease was also seen in the control miRNA groups, ruling out a sequence-specific *EBAG9* miRNA off-target effect (Figures S4A–S4D). Since no other disparities were observed, the possibility remains that, in miRNA-expressing CAR T cells, lower BCMA CAR and LAG-3 surface levels are connected to each other. Notably, LAG-3 associates with the TCR-CD3 complex and serves as a negative signaling regulator.[28]

Taken together, silencing of EBAG9 elevates the release of granzyme A by human CD8+ BCMA CAR T cells without affecting the secretion of inflammatory cytokines.

Knockdown of EBAG9 in CAR T cells confers enhanced cytotoxic effector functions *in vitro*

Conducting chromium release [51Cr] assays with isolated CD8+ T cells, we found that miRNA-mediated silencing of *EBAG9* in BCMA CAR T cells translated into more potent killing of BCMA+ MM (OPM-2) and B-NHL (DOHH-2, JeKo-1) target cell lines. Specifically, CD8+ BCMA CAR T cells killed the BCMA^{high} cell line OPM-2 at a rate that was approximately 1.5-fold higher with EBAG9 silencing than without it (Figure 3A). Effective dose levels to achieve the maximal killing rate of control BCMA CAR-transduced CD8+ T cells (at E:T ratio of 80:1) were about one-fourth to one-eighth lower for EBAG9-silenced BCMA CAR T cells (at E:T ratio of 20:1 to 10:1).

Using a second *EBAG9*-specific miRNA (miR-H17) with an antisense sequence that was shifted by two nucleotides compared with the miR-H18, we could prove that CD19 CAR T cells gained a downregulation of EBAG9 (Figures S5A and S5B) similar to miR-H18. To be able to assess the therapeutic benefit of EBAG9 silencing more adequately, we lowered effective T cell numbers to levels where CAR T cells with unaffected EBAG9 expression were no longer effective in controlling the growth of their targets. To this end, retroviral transduction rates were adjusted to around 15% by adding non-transduced T cells. miR-H17 doubled the cytotoxic activity of CD8+ CD19 CAR T cells and, as a consequence, substantially lowered the number of T cells needed to achieve maximal target cell lysis of unmodified CD19 CAR T cells (Figures S5C and S5D). Taken together, CD8+ CAR T cells with silenced EBAG9 expression featured improved target cytolysis.

We next assessed the kinetics of miR-H18 BCMA CAR T cell killing via microscopy image-based real-time target cell analysis (Figure 3B). Knockdown of EBAG9 led to an accelerated elimination of the BCMA-expressing tumor cell lines DOHH-2 and JeKo-1 (BCMA^{low}). For EBAG9-silenced CAR T cells, the time required to achieve 50% of tumor cell killing was about 10 h shorter (median) compared with that of control BCMA CAR T cells (Figure 3C). For miRNA-modified SP6 and miR-H18 SP6 CAR T cells, cytolytic activity was absent (Figures 3B, S6A, and S6B). No off-target activity was observed for the BCMA-negative REH cell line (Figure S6A). Furthermore, miR-ctl and miR-TCRα BCMA CAR T cells exhibited the same cytotoxic activity as unmodified BCMA CAR T cells (Figures S6C and S6D).

Single-molecule fluorescence microscopy was applied to investigate whether miR-H18 BCMA CAR T cells exhibited an altered sensitivity toward the BCMA antigen. BCMA or miR-H18 BCMA CAR T cells were stimulated with planar glass-supported bilayers functionalized with fluorescently labeled BCMA antigen in increasing amounts. The ensuing antigen-triggered changes in intracellular calcium levels were measured (Figures 3D and S6E). Both BCMA and miR-H18 BCMA CAR T cells exhibited similar antigen sensitivities with a minimum of 1–10 molecules μm^{-2} required for CAR T cell activation. This indicates that CAR signaling in the initial response to limiting antigen is not affected after EBAG9 silencing.

To assess the kinetics of the degranulation process at the immunological synapse (IS), antigen-dependent degranulation of BCMA CAR T cells was measured by CD107a mobilization and intracellular granzyme A staining (Figures 3E and 3F). While EBAG9 downregulation had no influence on the total amount of intracellular granzyme A (Figure S6F), the proportion of CD8+ miR-H18 BCMA CAR T cells actively recruiting lytic granules into the IS increased by about one-third compared with controls (Figure 3F, left). On the contrary, CD107a staining of CD4+ CAR T cells was not affected after this short period of stimulation (Figure 3F, right). Total miR-H18 BCMA CAR T cells were magnetically separated in CD4+ and CD8+ subsets. As shown in a 1:1 E:T co-culture assay, CD8+ miR-H18 BCMA CAR T cells lysed BCMA^{high}- (OPM-2, NCI-H929) as well as BCMA^{low}-expressing target cells (JeKo-1) efficiently. This was not the case for CD4+ CAR T cells targeted at BCMA^{low} tumor cells (Figure S6G). No difference was seen for BCMA^{high} MM cells. Taken together, targeting EBAG9 to increase the cytotoxic function of CD8+ CAR T cells is not restricted to a specific CAR but is rather a universally applicable strategy to target the cytolytic capacity of CD8+ CTLs.

signed rank test was performed. *$p < 0.05$, ns, not significant. (F) CAR T cells were co-cultured with the indicated target cell lines (1:1 ratio) for 24 h. The BCMA-negative Jurkat cell line served as a control. Supernatants were analyzed for cytokine secretion by ELISA. Bars represent mean ± SEM of n = 2 (TNF-α, IL-2), n = 3 (IFN-γ) experiments with n = 3 (IL-2), n = 4 (TNF-α), and n = 6 (IFN-γ) independent donors per group. (G) Quantification of flow cytometric analysis of key immune markers for CD8+ CAR T cells at day 7 after activation. Bars represent mean ± SEM of one experiment with n = 3 (BCMA CAR) and n = 4 (UT, miR-H18 SP6 CAR, miR-H18 BCMA CAR) independent donors per group. See also Figures S3 and S4.

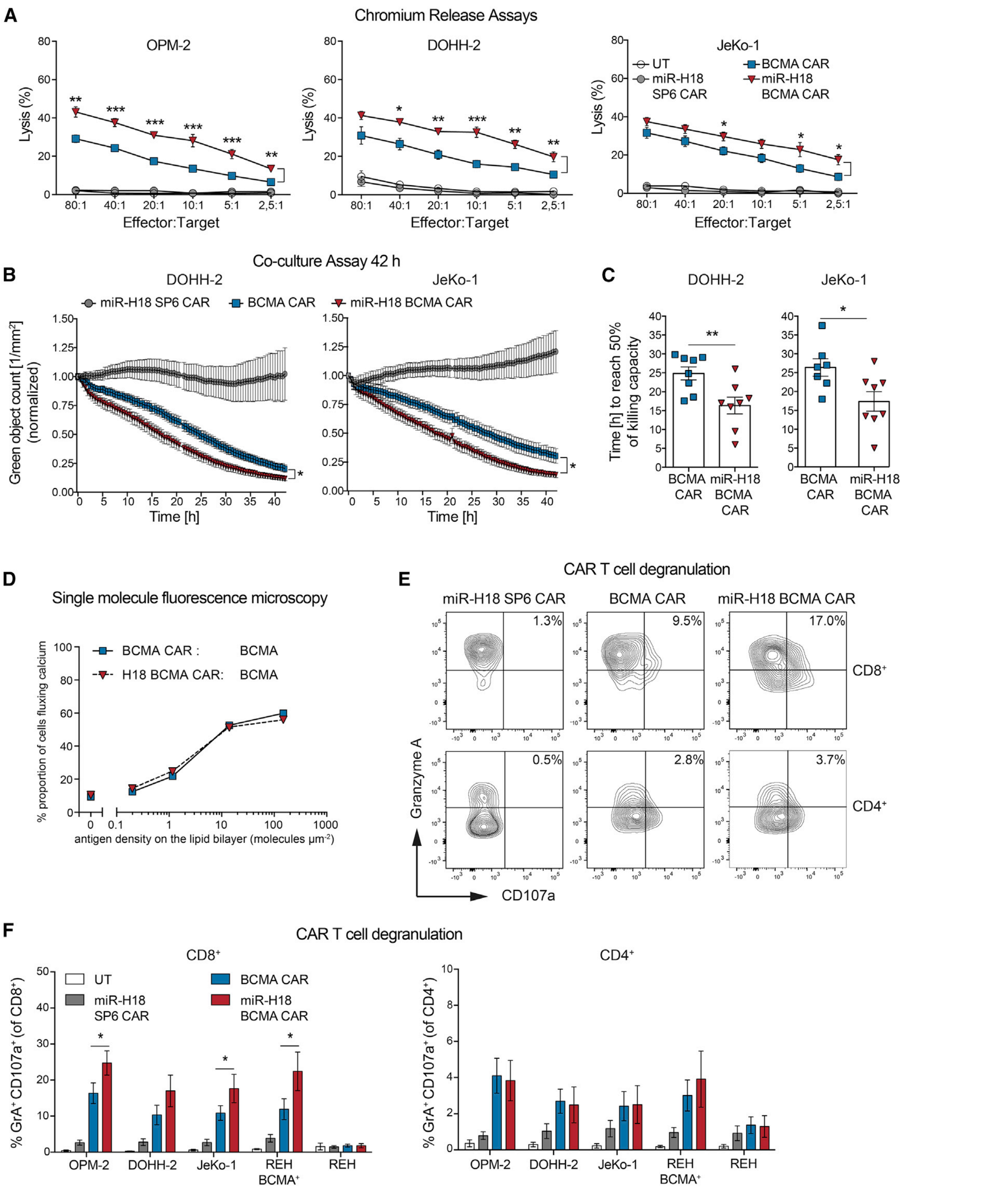

Figure 3. RNAi-mediated downregulation of EBAG9 endows human CAR T cells with enhanced antigen-specific cytotoxic effector functions

(A) *In vitro* cytotoxicity assays were performed by co-culturing CD8+ CAR T cells for 4 h with [51Cr]-labeled target cell lines. [51Cr] release was measured in duplicates. Data represent mean ± SEM of n = 5 experiments for each cell line with n = 4 (miR-H18 SP6 CAR), n = 7 (miR-H18 BCMA CAR), and n = 8 (UT, BCMA CAR) independent donors per group. A Mann-Whitney U test was applied. $^{*}p < 0.05$, $^{**}p < 0.01$, $^{***}p < 0.001$. (B) The kinetics of CAR T cell cytotoxicity was assessed by analyzing co-cultures of CAR T cells and GFP-expressing target cells (5:1 ratio) in an IncuCyte for 42 h. Fold change in tumor fluorescence intensity was measured in triplicates. Data represent

(legend continued on next page)

The versatility of miRNA-mediated downregulation of *EBAG9* was further validated using TCR-engineered CD8+ T cells. Therefore, the BCMA CAR of the miR-H18 vector was exchanged against a clinically relevant TCR that recognizes the tumor-associated antigen NY-ESO-1^{aa}157-165 in the context of HLA-A*0201.[29] Transduction of CD8+ T cells resulted in lower NY-ESO-1 TCR surface expression if the miRNA was co-expressed from the vector (Figures S7A and S7B). Downregulation of EBAG9 protein by the miR-H18 in CD8+ T cells was in the range of 80% (Figure S7C). To assess T cell effector functions, we next adjusted the CD8+ TCR-transduced T cells (mean transduction rate NY-ESO-1 TCR, 40%; miR-H18 NY-ESO-1 TCR, 28%) to similar transduction rates by adding non-transduced cells and then co-cultured with either NY-ESO-1 or MAGE-A1 peptide-pulsed T2 cells. Irrespective of EBAG9 silencing, no differences in IFN-γ responses to the NY-ESO-1 peptide were observed. Of note, the negative control peptide MAGE-A1 did not give rise to any effector functions (Figure S7D).

TCR-engineered CD8+ T cells were further co-cultured with NY-ESO-1 peptide-pulsed T2 cells. At a peptide concentration of 10 nM, target cell lysis for EBAG9-silenced CTLs was significantly more efficient. Half-maximal target cell killing by effector miR-H18-NY-ESO-1 TCR CD8+ cells occurred with half of the number of cells that were required when employing control NY-ESO-1 TCR CD8+ T cells without silenced EBAG9 (Figure S7E). The improved cytolytic activity was antigen specific as even 10^{-6} M MAGE-A1 peptide-pulsed T2 cells were not killed. CTLs with silenced EBAG9 elicited a stronger *in vitro* killing over a wide range of peptide concentrations (Figure S7F). Enhanced cytolytic activity was also confirmed for the MM cell line U266, which endogenously expresses the NY-ESO-1 antigen (Figure S7G).

Notably, as for the BCMA CAR, a therapeutic effect of EBAG9 silencing could be better visualized when TCR-transduced T cells were kept low by adjusting retroviral transduction rates by addition of non-transduced T cells. Thus, killing rates in this lactate dehydrogenase (LDH) release assay (Figure S7G) were less than 40% for the miR-H18-NY-ESO-1 TCR (E:T 10:1), and only 20% for the NY-ESO-1 TCR.

CAR T cells with enhanced cytolytic activity maintain their effector functions and proliferative capacity through cycles of repeated antigen exposure

To test whether EBAG9 downregulation in the context of an extended antigen-specific CAR T cell stimulation results in overactivation and activation-induced cell death (AICD), an *in vitro* serial transfer model was employed.[30] BCMA CAR and miR-H18 BCMA CAR T cells were co-cultured with BCMA+ MM.1S cells at a 1:1 ratio. Remaining tumor cells were quantified at 72-h intervals, with CAR T cells being transferred to fresh target cells over five successive rounds (Figure 4A).

BCMA CAR and miRNA-edited BCMA CAR T cells eliminated tumor cells to subtotal levels throughout the repetitive antigen-stimulation cycles (Figures 4B and 4C). IFN-γ secretion declined slowly to one-third of the amount measured in round 1 (Figure 4D). The proliferative capacity of CAR T cells between transfer cycles #2 and #5 remained at least 2-fold higher compared with day 0 of each cycle (Figure 4E). Accordingly, more than 90% viable T cells (7-aminoactinomycin [AAD] negative) were present throughout all transfer rounds (Figures 4F and S8A). Over the course of the experiment, the CAR+ T cell population was enriched up to 90% (Figure 4G).

We observed a predominant occurrence of memory T cells (CD45+RO+; CD45+RA−) upon repetitive antigen exposure (Figures S8B and S8C). The proportion of $T_{effector\ memory}$ (TEM) (CCR7^{low}CD62L^{low}) cells increased over time at the cost of $T_{central\ memory}$ (TCM) (CCR7^{high} CD62L^{high}) (Figures S8B and S8D). Differences resulting from the knockdown of EBAG9 in CAR T cells were not evident.

The results from repetitive antigen stimulation of EBAG9-silenced CD19 CAR T cells confirmed the findings on BCMA CAR T cells. CD19 CAR and miR-H17 CD19 CAR-transduced T cells kept their effector and proliferation capacity over five rounds without any evidence of exhaustion or activation-induced cell death (AICD) (Figures S9A–S9G). Of note, while the cytolytic strength of the CAR T cells was maintained (Figure S9A), the IFN-γ response declined over five rounds of stimulation (Figure S9B), indicating that the two functions are not necessarily coupled.

Taken together, the preserved functional properties of EBAG9-silenced CAR T cells are not correlated with AICD or hallmarks of exhaustion.

Silencing of EBAG9 in CAR T cells is associated with a unique transcriptional profile

We performed transcriptome profiling to assess the possibility that transcriptional alterations induced by miR-H18-mediated *EBAG9* silencing affect CAR T cell differentiation, metabolomic state,

mean ± SEM of n = 3 experiments with n = 6 (UT, miR-H18 SP6) and n = 8 (BCMA CAR, miR-H18 BCMA CAR) independent donors per group. A multiple t test (one per row) was applied. *p < 0.05. (C) Quantification bar plot of the time to reach 50% cytotoxicity of targeted tumor cell lines in (B). Data are shown as mean ± SEM. A Mann-Whitney U test was applied. *p < 0.05, **p < 0.01. (D) Fura-2-loaded CAR T cells were stimulated on supported lipid bilayers loaded with indicated numbers of fluorescently labeled BCMA antigen molecules. Proportion of cells fluxing calcium were plotted as a function of antigen density (molecules μm^{-2}). Data for one representative donor out of two are shown (n = 2 independent experiments). The number of cells assayed for each data point in the shown plot ranged from n = 556 to 1,434 (median n = 700). (E) Representative flow cytometry dot plot of CAR T cell degranulation. CAR T cells were co-cultured with different target cell lines (1:1 ratio) in the presence of anti-CD107a antibody for 2 h; exemplarily data shown are for JeKo-1. Frequencies of CD4+ or CD8+ CD107a+ granzyme A + CAR T cells are indicated as percentages in the gates. (F) Quantification bar plot of (E). BCMA-negative REH cells were used as a negative control. REH BCMA+ were stably transduced with BCMA. Data are shown as mean ± SEM of n = 2 experiments with n = 5 independent donors per group. A Mann-Whitney U test was applied. *p < 0.05. See also Figures S5–S7.

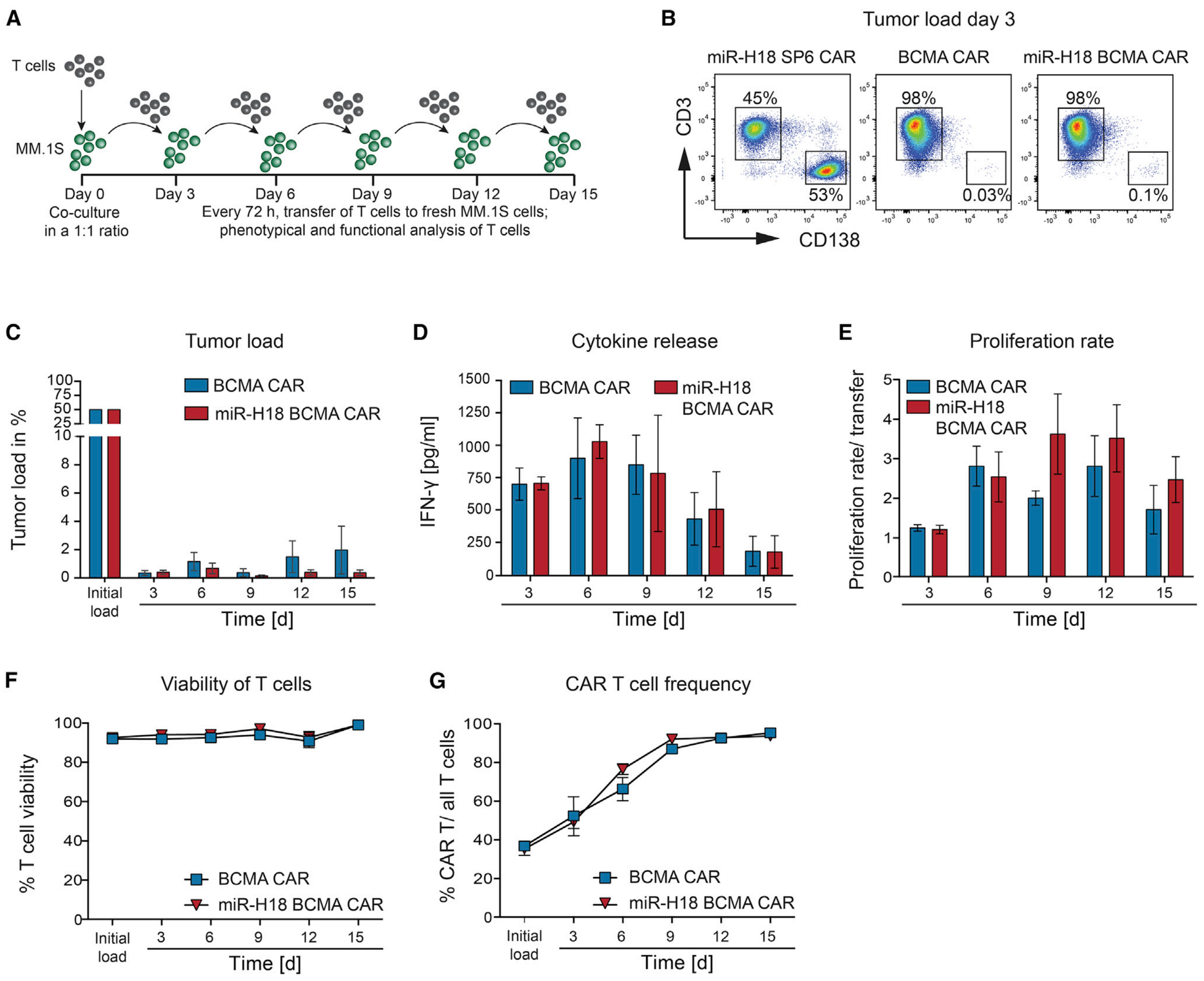

Figure 4. EBAG9 silenced CAR T cells maintain effector functions, viability, and proliferative capacity upon *in vitro* recursive antigen exposure

BCMA CAR T cells and miR-H18 BCMA CAR T cells at day 10–13 after start of cultivation were co-cultured with MM.1S (GFP+ CD138+) target cells at a 1:1 ratio. Transduction rates were adjusted to 30%–40% by addition of UT. After 72 h, CAR T cells were transferred to fresh target cells for a total of five rounds. Data are depicted as mean value ±SEM of n = 2 experiments with n = 3 independent donors per group. (A) Schematics of the repetitive antigen-stimulation assay. (B) One representative dot plot analysis for determining viable MM.1S target cells after each round by flow cytometry analysis. Frequencies of 7AAD− CD138+ CD3− MM.1S cells are indicated as percentages on the gate. (C) Quantification bar plot of (B). (D) Cell-free supernatants were collected for measuring IFN-γ secretion by ELISA. (E) The proliferation rate was assessed by manually counting of viable T cells and removing CD138+ MM.1S cells by magnetic bead sorting. The ratio of T cells present after each cycle of co-cultivation versus the number of input T cells was calculated. (F) Viability of CAR T cells (CD3+ IgG+) as determined by 7-AAD staining and flow cytometry analysis. (G) Frequencies of transduced CD3+ CAR+ T cells among total CD3+ T cells are depicted and expressed as percentages. See also Figures S8 and S9.

functional activity, or safety. To this end, we sorted human T cells that were transduced with the BCMA CAR alone, or together with miR-H18. Bulk mRNA sequencing at day 10 after activation revealed variabilities among individual donors, but principal component analysis highlighted a clustering dependent on the suppression of EBAG9 expression (Figure 5A). A total of 1,560 genes were called differentially expressed in miR-H18 CAR T cells, with 724 genes being upregulated, and 836 genes downregulated compared with the control.

To gain insight into the biological processes affected by miR-H18, we performed over-representation analysis[31] and unbiased gene set enrichment analysis (GSEA)[32] using gene lists from the Molecular Signature Database.[33]

The over-representation of upregulated genes in miR-H18 BCMA CAR T cells showed that such genes were significantly enriched in immunological gene signatures, associated with T cell activation and effector function. Other enriched gene sets included pathways

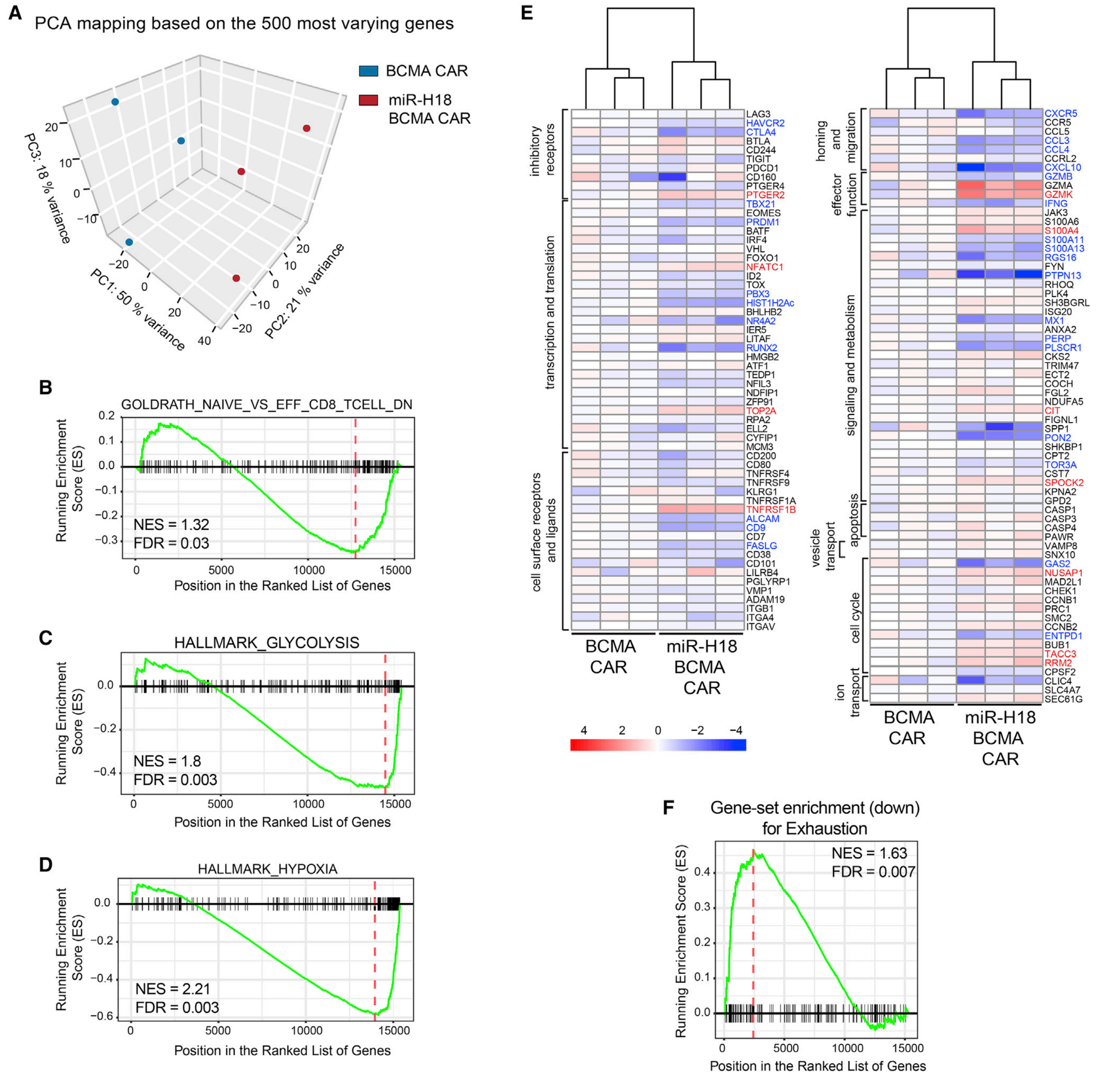

Figure 5. Transcriptome analysis of EBAG9 silenced BCMA CAR T cells by mRNA-seq reveal a unique transcriptional profile

(A) Principal component analysis (PCA) of global transcriptional profiles of BCMA and miR-H18 BCMA CAR T cells at day 10 of culture generated from three independent donors. (B–D) Representative GSEA results from functional comparison of BCMA versus miR-H18 BCMA CAR T cells using 18,668 MSigDB gene lists (version 6.5). Gene lists have been selected to show the miR-H18 effect on T cell activation, hypoxia, and glucose metabolism gene signatures. Horizonal axis shows the rank of genes by increasing log2 fold change, so genes ranked low are downregulated genes, and genes with large ranks are upregulated genes. Gene lists' ranks are shown on the x axis. In all three lists, the genes are mostly clustered at the upregulated end. The dotted red vertical line shows the leading edge position, where the enrichment score is most extreme. Normalized enrichment score (NES) and the p value adjusted for multiple testing correction (false discovery rate –[FDR]) are provided for each example. (E) Bulk RNA-seq identifies expression signatures distinguishing BCMA CAR and miR-H18 BCMA CAR-transduced CAR T cells. Heatmaps display normalized expression value residuals after subtraction of the patient effect for an exhaustion-related gene set (Table S1). Expression value residuals are presented on a log2 scale. (F) GSEA of the exhaustion signature shown in (E). The gene ordering is identical to the ranking used for (B)–(D), showing an enrichment of exhaustion signature genes among genes downregulated in miR-H18 BCMA CAR-transduced CAR T cells. See also Figure S10.

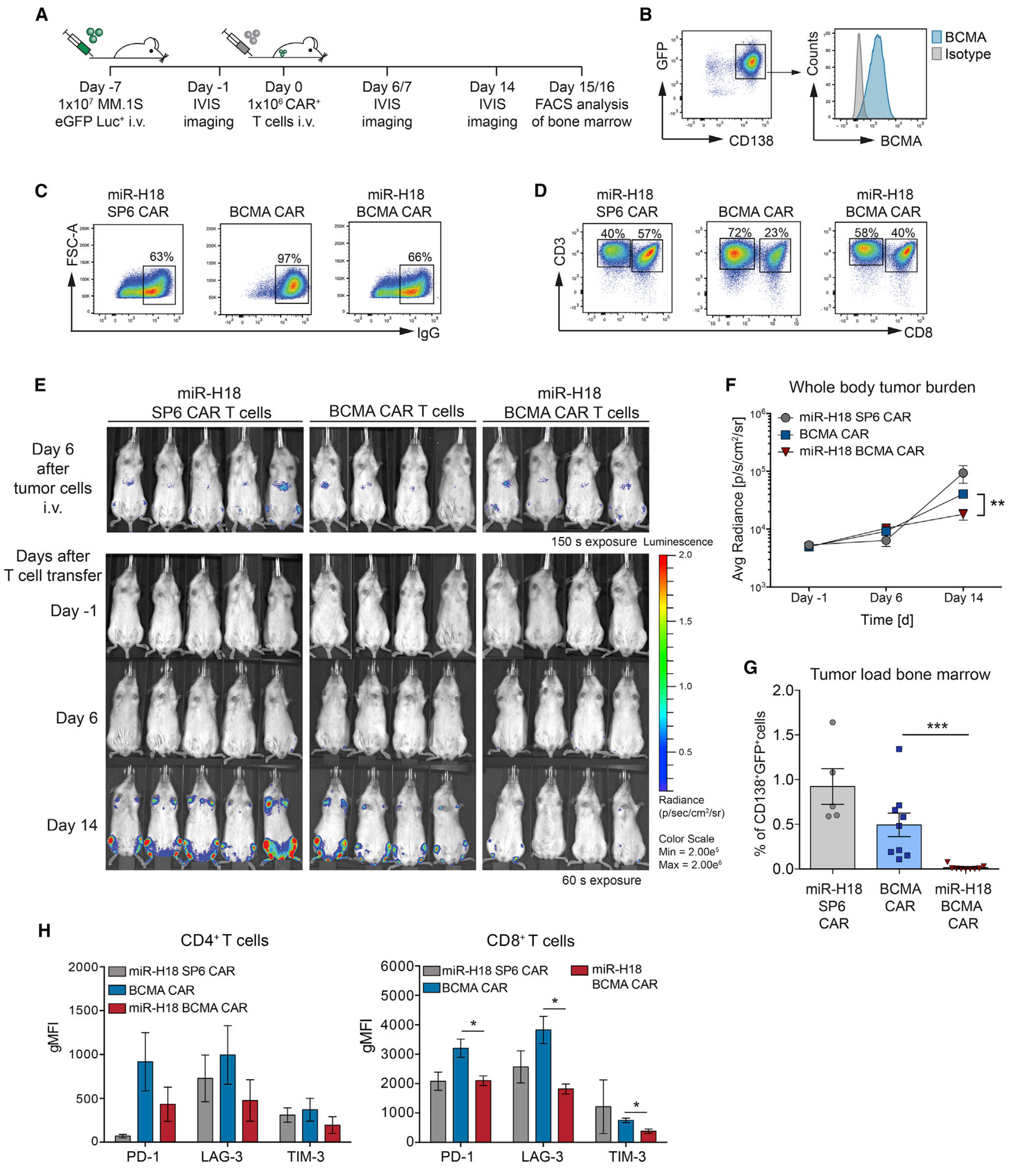

Figure 6. Engineered human BCMA CAR T cells with silenced EBAG9 eradicate MM cells *in vivo* more efficiently

(A) Schematic overview of the experimental procedure. (B) Flow cytometric analysis of MM.1S tumor cell line. (C) Prior to transfer, CAR T cells were enriched for IgG+ T cells. Transduction rates after enrichment are indicated by numbers on the gate. (D) Co-staining of CD3 and CD8 was used for analysis of CAR T cell subset composition. CD3+CD8+ double-positive cells were defined as CD8+ T cells, whereas CD3+CD8− cells were considered as CD4+ T helper cells. Percentages of T cell subsets are

(legend continued on next page)

that are associated with response to hypoxia and glucose metabolism (Figures 5B–5D and S10A–S10C). We sorted CAR+ T cells and validated key genes that contribute to glycolysis and hypoxia. In addition to the RNA-seq data (Figures 5E and S10A–S10E), miR-ctl and miR-TCRα BCMA CAR T cells were included. Augmented expression of *PPFIA4* and *ALDO* could be confirmed in miR-H18 BCMA CAR-expressing T cells (Figure S10D).

Applying GSEA, key genes contributing to exhaustion were found downregulated in miR-H18 BCMA CAR T cells (Figures 5E; Table S1). In particular, genes encoding inhibitory receptors such as *CTLA4*, *TIGIT*, and *HAVCR2* (TIM-3) were repressed, whereas *LAG3* was unaltered. Likewise, genes encoding for exhaustion-related transcription factors, such as *TBX21* (T-bet), *PRDM1* (Blimp-1), and *RUNX2* were downregulated. Interestingly, while the expression of *GZMB* was downregulated, *GZMA* and *GZMK* showed a trend toward an upregulation.

To assess the risk of unintended gene alterations upon miRNA-modification, we analyzed the expression of oncogenes and tumor suppressor genes.[34] Although fewer genes were differentially expressed than expected by chance, five oncogenes (*IDH2*, *PPP2R1A*, *MAP2K1*, *IDH1*, and *SETBP1*) and three tumor suppressor genes (*DAXX*, *FUBP1*, and *MAP3K1*) were significantly upregulated, while seven tumor suppressors (*PAX5*, *RNF43*, *ACVR1B*, *PRDM1*, *CREBBP*, *BCOR1*, and *NF1*) were significantly downregulated (Figure S10E; Table S2). Collectively, exhaustion-related genes were downregulated in EBAG9-knockdown BCMA CAR T cells, indicating that gain of cytolytic competence did not negatively affect effector T cell fitness.

EBAG9-specifc miR-H18 enhances the therapeutic potential of BCMA CAR T cells *in vivo*

We examined the extent to which EBAG9 downregulation affected the anti-tumor response of BCMA CAR T cells in a NOD.Cg-Prkdcscid Il2rg tm1 Wji/Szj (NSG) mouse xenotransplantation model (Figure 6A). Engraftment of MM.1SeGFP-luc cells (CD138+ GFP+; Figure 6B) and tumor progression were monitored by *in vivo* bioluminescence imaging (BLI). We chose to adoptively transfer CAR+ T cells at numbers that proved in our hands too low for tumor clearance when employing BCMA CAR T cells with unmodified EBAG9 expression.[24] Mice were treated with a single dose of 1×10^6 CAR+ T cells at day 7 after MM.1SeGFP-luc transplantation (Figures 6C and 6D). We observed strong myeloma progression localized to typical myeloma sites in bone marrow of miR-H18 SP6 CAR T cell-treated mice over 2 weeks, significantly less expansion in the BCMA CAR T cell cohort, and potent suppression of tumor growth in the miR-H18 BCMA CAR T cell group (Figures 6E and 6F). This bioimaging result corresponded to the tumor load (GFP+CD138+) in bone marrow at the end of the observation period (day 15–16), at which point essentially all MM.1SeGFP-luc cells had disappeared in mice treated with miR-H18 BCMA CAR T cells (Figure 6G). Human CD4+ CAR T cells isolated from the bone marrow revealed no significant differences in expression of the exhaustion markers PD-1, LAG-3, and TIM-3. Expression of exhaustion markers in CD8+ CAR T cells was lower in miR-H18-engineered BCMA CAR T cells compared with controls (Figure 6H). Our results imply that EBAG9 silencing did not promote exhaustion or AICD *in vivo*. Collectively, EBAG9 silencing endows BCMA CAR T cells with a considerably improved capacity to reduce tumor growth *in vivo*, as even low numbers of killing-enhanced CAR T cells gained control over aggressive tumor progression.

Next, we expanded the observation time window and further challenged the number of CAR+ T cells infused into tumor-challenged mice through a single application of 7×10^5 effector cells (Figure 7A). Transduction rates, CAR surface expression density, and CD4/CD8 ratios were almost identical for the BCMA CAR and the miR-H18 BCMA CAR group (Figures 7B and 7C). To avoid experimental complications arising from xenoreactive graft-versus-host disease, which may occur in the NSG model, we limited the observation time to 4.5 weeks.[35] We obtained a statistically significant delay of tumor progression in the miR-H18 BCMA CAR group compared with the BCMA CAR cohort (Figures 7D–7F). Apart from a modestly increased TIM-3 expression in CD4+ T cells, differences in the expression of exhaustion markers were not observed between the treatment cohorts (Figures 7G and 7H). Tumor outgrowth of MM was not associated with a loss or downregulation of BCMA on MM.1SeGFP-luc cells (Figure 7I). Taken together, although the infusion of a limited number of miRNA-engineered CAR T cells failed to eradicate transplanted tumors in this challenging experimental setting, progression of MM was significantly slowed down compared with that observed in mice treated with EBAG9-unsilenced T cells modified with either a BCMA or irrelevant CAR.

DISCUSSION

The findings reported herein demonstrate the considerably improved therapeutic potency and safety of EBAG9-silenced T cells and suggest their clinical application as a promising option for optimized cancer immunotherapy. In mice, deletion of the biochemically defined regulator of lytic granule release, EBAG9, accelerated the allorejection of

indicated by numbers on the gate. (E) NSG mice were challenged by i.v. transplantation of 1×10^7 MM.1S cells stably expressing a firefly luciferase. Tumor cell growth was visualized by BLI (exposure: 150 s) at day 6 after tumor inoculation. Mice received 1×10^6 CAR + T cells 1 day later, and subsequent BLI was for 60 s. Serial BLI exposures of miR-H18 SP6 CAR T cell-, BCMA CAR T cell-, and miR-H18 BCMA CAR T cell-treated animals are shown. (F) Mean values ±SEM of bioluminescence signal intensities obtained from the entire body (total flux) were plotted for each group and time point, with n = 5 (miR-H18 SP6 CAR T cells) and n = 9 (BCMA CAR T cells, miR-H18 BCMA CAR T cells) animals per group. (G) Femurs were flushed, and CD138+GFP+ tumor cells were quantified by flow cytometry at day 15 and 16 after CAR T cell transfer. Mean values ±SEM of two independent experiments are depicted. A Mann-Whitney U test was employed. ***$p < 0.001$. (H) Quantification bar graph of flow cytometry CAR T cell analysis in the bone marrow as obtained in (D). Staining for the detection of CAR T cell exhaustion markers was done. Values are expressed as gMFIs. Bars represent mean values ±SEM. A Mann-Whitney U test was applied. *$p < 0.05$.

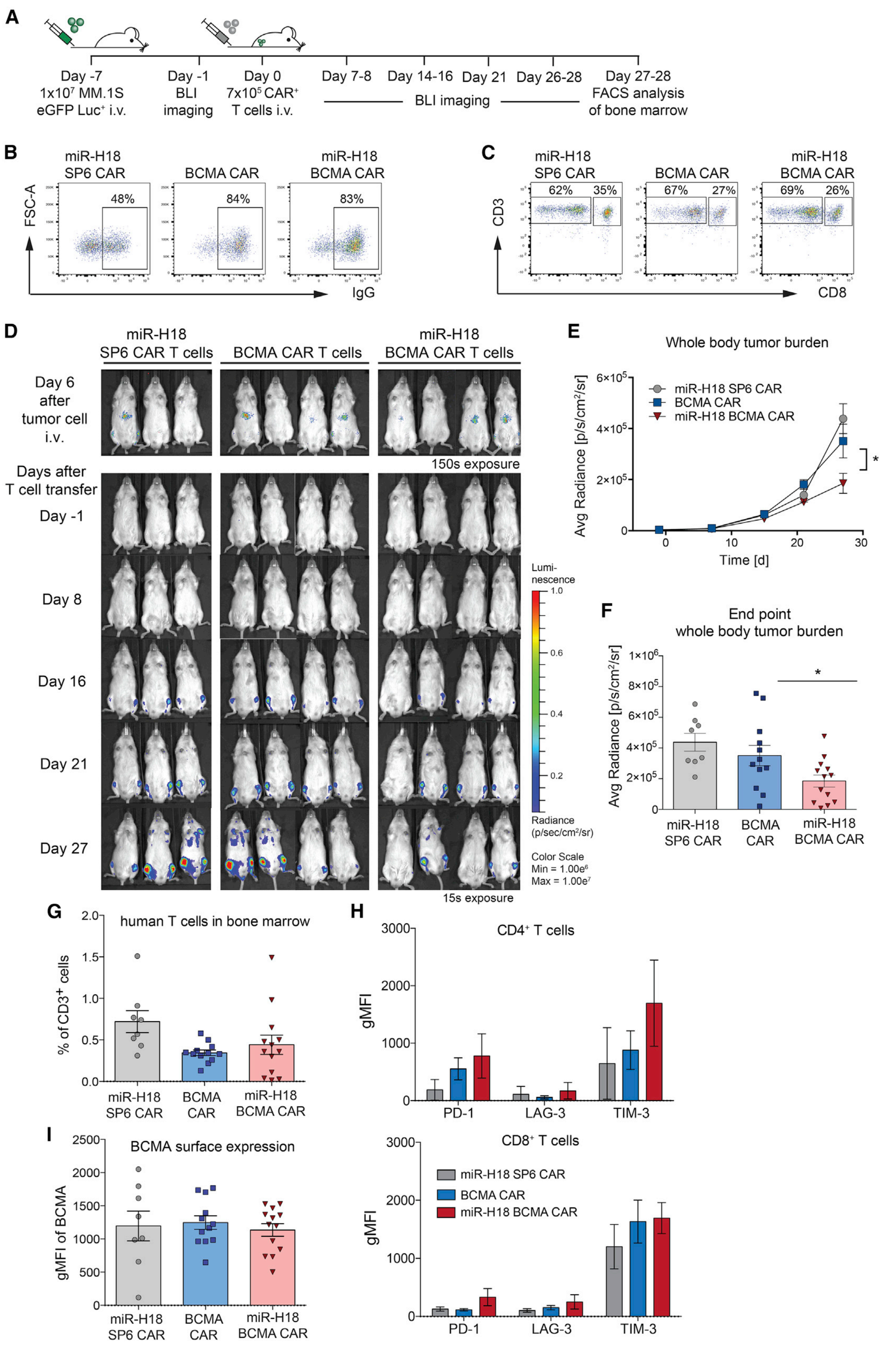

(legend on next page)

miHag-mismatched transplants substantially. This implied that control of the secretory pathway exerted an immune checkpoint function by limiting the cytolytic capacity of CD8+ T cells and hence favoring tolerance. We infer from the overall health status of our *Ebag9*-deleted mouse strain that unleashing the antigen-specific cytolytic CTL response, as mediated by granzyme and perforin, does not result in overt immunopathology.[20] Importantly, the constitutive secretory route employed by the effector cytokines IFN-γ, TNF-α, and IL-2 remained unaffected by the reduced EBAG9 function and, thus, the risk of cytokine storm induced by cytokines released from activated CAR T cells is mitigated.[36]

The immunological phenotype of genetically manipulated mice suggested that engineered regulation of EBAG9 expression in T cells is an innovative and translatable strategy for ATT in the clinics. We validated that silencing of EBAG9 is not only adequate to improve the anti-tumor response of conventional but also of CAR-modified T cells, without compromising the safety of CAR T cell therapy. Our findings regarding the gain of cytolytic capacity are in agreement with EBAG9 being a negative regulator of cytotoxic granule release. Mechanistically, increased CD107a staining in CAR T cells indicates that sorting of cytotoxic effector molecules to lytic granules and endosomal-lysosomal trafficking proceeds more efficiently when the EBAG9 brake is released. While EBAG9 does not act directly on the release process at the IS, it is likely that sorting to and refilling of secretory lysosomes is improved.[19,20] Single-molecule fluorescence microscopy of miRNA-modified BCMA CAR T cells excluded the possibility that CAR-proximal signaling is altered.

The CAR T cell response is regulated by target antigen and CAR surface density, as sub-threshold expression of either one results in low anti-tumor efficacy.[37] CAR affinity enhancement may allow stabilization of the T cell-tumor cell interaction even when antigens or CARs are displayed at low numbers. However, high-affinity CARs impose a substantial risk for several tumor-associated antigens that are expressed on benign tissues as well.[38] By facilitating exocytosis of granzyme A, EBAG9 silencing may compensate for a loss in therapeutic index, which may accompany engineering efforts aimed at mitigating on-target/off-tumor effects through reduction of CAR-antigen affinity. Taken together, our study provides compelling evidence that EBAG9 silencing increases the CAR T cell cytolytic response, which could be exploited to render CAR T cell therapy more effective and safer.

Recently, other inhibitory factors affecting T cell activation programs in solid tumors were described. Examples include the transcription factors *NR4A1–3*, the nuclear factor *Tox*, and the RNA helicase *Dhx37*.[39–41] The efficacy profiles of these factors, with regard to anti-tumor effects, have been demonstrated *in vitro* and, in some cases, in mouse solid tumor models. However, the safety profiles of genetic deletion will need to be demonstrated, with emphasis on the potential for autoimmune responses or, conversely, exhaustion. In contrast, for EBAG9 silencing, the gain in cytolytic potency depends on a selective perturbation of the regulated secretory pathway. Analysis of the immunophenotype of EBAG9-silenced CAR T cells under repetitive antigen-stimulation conditions largely excluded the potential risk of AICD or functional exhaustion.[27,42] Consistent with this, gene expression profiling confirmed that EBAG9 silencing did not create conditions that are typically associated with enhanced T cell exhaustion.

Transcriptome profiles of EBAG9-silenced CAR T cells indicated metabolic reprogramming with enhanced aerobic glycolysis. This potential metabolic alteration may boost rapid cytotoxic effector CAR T cell function.[43] However, more definite conclusions require validations of CAR T cells' metabolic state. In contrast to the metabolic differences observed between the 4-1BB and the CD28 co-stimulatory domains in CD19 CAR T cells,[44] differences in memory fate decisions (CD45+RO, CD45+RA) were not observed between EBAG9-downregulated and control CAR T cells *in vitro*. In solid tumors, cytotoxic activity of ATT often better correlates with the availability of IFN-γ, which is targeted preferentially at tumor stroma cells.[45] In contrast, hematopoietic tumors have more penetrable barriers created by the tumor microenvironment and more frequently give rise to productive IS. Thus, as CAR T cell design is guided by achieving tumor lysis via direct T cell-tumor cell interaction, we conclude that CAR T cell efficacy strongly depends on the recruitment of the perforin/granzyme cytolytic pathway. In view of the restricted cell biological effects of EBAG9, we consider it likely that EBAG9 downregulation is better suited to improve T cell targeting of hematologic malignancies. Notably, the EBAG9-defined checkpoint in effector molecule secretion is not only applicable to enhance CAR T cell function but can be demonstrably adapted to TCR-engineered CD8+ T cells as well.

Having the miRNA and the CAR expressed as a polycistronic mRNA from the same vector has important advantages. The expression of

Figure 7. BCMA CAR T cells with silenced EBAG9 delay MM relapse

(A) Schematic overview of the experimental procedure. (B) Transduction rates of CAR-transduced T cells as indicated by percentages on the gates. (C) Co-staining of CD3 and CD8 was used for analysis of CAR T cell subset composition. Percentages of T cell subsets are indicated by numbers on the gate. (D) NSG mice were challenged by i.v. transplantation of 1×10^7 luciferized and GFP+ MM.1S cells. Tumor cell growth was visualized by BLI at day 6 after tumor inoculation. Mice received 7×10^5 CAR+ T cells 1 day later, followed by BLI. Serial BLI exposures of miR-H18 SP6 CAR T cell-, BCMA CAR T cell-, and miR-H18 BCMA CAR T cell-treated animals are shown. (E) Mean values ±SEM of BLI obtained regions of interest covering the entire body were plotted for each group and time point for each group and time point with n = 8 (miR-H18 SP6 CAR T cells), n = 12 (BCMA CAR T cells), and n = 13 (miR-H18 BCMA CAR T cells) animals per group. (F) BLI intensities at the end of the observation period; combined data from animals between 4 and 4.5 weeks (d27, d28, d34). Mean values ±SEM of n = 3 independent experiments are depicted. An unpaired Student's t test was applied. *$p < 0.05$. (G) Human CD3+ T cells in bone marrow. Bars represent mean values ±SEM. Data are from n = 2 experiments. (H) Exhaustion marker expression in T cells after adoptive transfer into tumor-bearing mice. Quantification bar graph of flow cytometry CAR T cell analysis in the bone marrow. Values are expressed as gMFIs. Bars represent mean values ±SEM. (I) BCMA expression on GFP+ MM.1S tumor cells derived from flushed bone marrow. Bars represent gMFI of BCMA ±SEM of n = 8 (miR-H18 SP6 CAR T cells), n = 12 (BCMA CAR T cells), and n = 13 (miR-H18 BCMA CAR T cells) animals per group. Data in (G)–(I) were not significantly different.

both genes is tightly coupled and T cells with the desired phenotype can be generated in a single transduction step. The efficiency of this process is similar to commonly used retroviral transduction protocols used for clinical CAR T cell products. We performed a genotoxic risk assessment applying bulk RNA sequencing (RNA-seq) of paired BCMA CAR and miR-H18 BCMA CAR T cell products. Statistically, fewer differentially expressed genes than expected by chance were altered, including a limited number of moderately deregulated oncogenes and tumor suppressor genes.[34] Importantly, expression of the most frequently mutated genes in T cell lymphoma or leukemia (i.e., *DNMT3A*, *TET2*, *JAK*, *NOTCH1*, *CDKN2A*, *PTEN*, and *FBXW7*) was not altered in miR-H18-BCMA CAR T cells.[46,47] We conclude that expression of an *EBAG9*-specific miRNA has an acceptable risk profile that does not go beyond the risk associated with retroviral insertion alone.[48]

CRISPR/Cas9 gene editing holds promise for immune cell engineering and provides an alternative to retroviral vectors encoding miRNAs and an antigen receptor. However, so far, most protocols use a multistep process, including electroporation, transduction, or enrichment, which are more difficult to translate into Good Manufacturing Practice (GMP) protocols.[49]

The enhancement of cytolytic activity of transgenic T cells can solve current problems in ATT. First, for a relevant number of cancer patients who receive myelosuppressive chemotherapy, the availability of sufficient lymphocyte starting material for CAR T cell manufacturing poses a severe limitation.[10] EBAG9 engineered CD8+ T cells perform considerably better than their non-engineered counterparts at equally low transduction rates or low effector frequencies. Therefore, it might be possible to process and transplant lower numbers of the T cell product but obtain sufficient anti-tumor efficacy. Second, it is of substantial advantage to deepen the minimal residual disease (MRD) state [50] to prevent tumor relapse. Here, the amplification of cytolysis enhances the opportunity for tumor eradication. Finally, in otherwise tolerant female recipient animals, HY miHag was turned into a rejection antigen by *Ebag9* deletion. It seems possible that recognition of weakly immunogenic antigens displayed on tumor cells may already trigger effector molecule release and, ultimately, target cell death.

The simple and safe EBAG9-silencing-based technology to outfit CTLs with augmented cytotoxicity is rapidly translatable into current virus-based manufacturing processes for clinical application, with considerable implications when aiming for a broadly available and economically viable treatment for cancer patients.

MATERIALS AND METHODS

Mice

NSG mice were purchased from The Jackson Laboratories and subsequently used as breeding pairs. C57BL/6 and $Rag2^{-/-}$ mice were purchased from Charles River. $Ebag9^{-/-}$ mice were generated as described.[20] All mice were housed and maintained under standard pathogen-free conditions at the animal facility of the Max-Delbrück-Centrum for Molecular Medicine (MDC) Berlin. In all experiments, mice were matched for their age, sex, and strain background. Recipient mice for adoptive transfer were used at the age of 8–12 weeks. All animal studies were conducted in compliance with the institutional guidelines of the MDC and approved by the Berlin State review board at the Landesamt für Gesundheit und Soziales, Berlin (registered under Landesamt für Gesundheit und Soziales TVV G0331/05, G0089/10, G0091/15, G0050/16).

Cell lines

The MM cell lines NCI-H929 and OPM-2, the B-NHL cell lines DOHH-2 and JeKo-1 (MCL), and the T-acute lymphoblastic leukemia (ALL) cell line Jurkat J76 were obtained from DSMZ (Braunschweig, Germany). The B-ALL cell line REH was obtained from Dr. Stephan Mathas (MDC, Berlin, Germany), and its authenticity was confirmed by a multiline authentication test (Multiplexon, Heidelberg, Germany). The cell line HEK-293T was purchased from Quantum Biotechnologies (QC, Canada). The luciferase-containing MM.1S cell line was previously generated in house.[51] The murine SV40-Tag tumor cell line Co16.113 was a kind gift from Dr. Gerald Willimsky (Charité, Berlin, Germany). T2 cells were donated by Dr. Simone Rhein (MDC, Berlin, Germany).

All human suspension cell lines were maintained in RPMI-1640 containing 10% fetal calf serum (FCS), 1% penicillin/streptomycin (Pen/Strep), 1% L-glutamine, 1% minimum essential medium non-essential amino acids (NEAAs), and 1% sodium pyruvate (Na-Pyr). The cell lines HEK-293T, 293VecGalV and Plat-E were maintained in DMEM containing 10% FCS, 1% Pen/Strep, and 1% Na-Pyr. Plat-E cells were further kept under selection (1 μg/mL puromycin, 10 μg/mL blasticidin). Upon receipt, all cell lines were expanded and aliquots were immediately frozen in liquid nitrogen. Frequent antibiotic treatments for mycoplasm removal were performed.

Generation of stable retrovirus-producing packaging cell lines

Stable retroviral particle-producing packaging cell lines were generated by retroviral transduction of 293Vec-Galv cells with the appropriate MP71-miRNA-CAR retrovirus. Cells were sorted for high CAR expression by goat anti-human immunoglobulin (Ig) G-Phycoerythrin (PE) (Southern Biotech) antibody staining and fluorescence-activated cell sorting (FACS). Stable producer cells were expanded and stocks were frozen immediately.

Generation of γ-retroviral constructs encoding the β and α chains of an affinity-enhanced, HLA-A2-restricted NY-ESO-1 peptide-specific TCR

The codon-optimized TCR cassette (*TCRβ-P2A-TCRα*) of an A*0201/NY-$ESO_{-157-165}$-specific TCR was synthesized (GeneArt; Thermo Fisher Scientific) and subsequently subcloned into the MP71 retroviral vector with or without an intronic miRNA (miR-H18) targeting *EBAG9*. The patient-derived TCR (IMGT: *TRBV6-5/TRAV21*01*) was affinity enhanced by the introduction of two mutations (G50A, A51E) into the β chain. To avoid mispairing with endogenous TCR chains, mouse TCR C regions carrying two additional non-native cysteines were employed. The codon-optimized TCR cassette

(*TCRβ-P2A-TCRα*) was subsequently subcloned into the MP71 retroviral vector with or without an intronic miRNA (H18) targeting EBAG9 via NotI- and EcoRI-mediated restriction cloning. Viral supernatants were generated by transient transfection of the 293Vec-Galv packaging cell line.

Isolation and culture of primary mouse T cells

Spleens of 10- to 12-week-old mice were isolated and passed through a 40-μm cell strainer to generate single-cell suspensions. After erythrocyte lysis, isolated splenocytes were seeded for activation onto six-well plates coated with anti-mouse CD3 (3 μg/mL; cl. 17A2; BioLegend) and anti-mouse CD28 (2 μg/mL; cl. 37.51; BioLegend) antibody for 24 h. Splenocytes were cultured in mouse T cell media (RPMI-1640, 10% FCS, 1% Pen/Strep, 1% L-glutamine, 1% NEAA, 1% Na-Pyr, and 0,1% β-mercaptoethanol [mTCM]) supplemented with 10 IU/mL rmIL-2 (Peprotech).

Isolation and culture of primary human T cells

Human peripheral blood mononuclear cells (PBMCs) were isolated from peripheral blood of healthy voluntary donors or buffycoats from anonymous healthy blood donors by density gradient centrifugation using BioColl (Biochrom) solution, essentially as described.[25] The recruitment of voluntary blood donors was conducted according to the Declaration of Helsinki and in accordance with local ethical guidelines. Enrichment of CD3+ or CD8+ T cells from freshly prepared or frozen PBMCs was achieved by magnetic cell separation, using either the Easy Sep Human T cell Isolation Kit (STEMCELL Technologies), or the CD8+ T cell Isolation Kit, Human (Miltenyi Biotec). T cells were stimulated with plate-bound anti-human CD3 (5 μg/mL; OKT3; BioLegend) and anti-human CD28 (1 μg/mL; CD28.2; BioLegend) antibody for 48 h. Cells were cultured in T cell medium (RPMI-1640, 10% FCS, 1% Pen/Strep, 1% L-glutamine, 1% NEAA, and 1% Na-Pyr [hTCM]) supplemented with either 100 IU/mL rhIL-2 or 10 ng/mL rhIL-7 and rhIL-15 (PeproTech or Miltenyi Biotec).

Generation of retroviral vectors

RNAi-target sites were identified using the Web-based RNAi target site prediction programs (WIsiRNA, BlockIT, siDesign, OligoWalk). Redirected miRNAs were designed by exchanging the guide and passenger strand of mouse miR-155 so that the new hairpin contained a central mismatch of two base pairs, as described previously.[24] Exchanging a 21-nucleotide-containing antisense sequence in the hairpin structure of the miRNA-155 against predicted mouse or human EBAG9 target site sequences led to the generation of different EBAG9-targeting miRNAs. The following sequence was chosen as non-targeting control sequence: 5′TAGGTGCTCTTCATCTTGTTG-3′ (miR-ctl). The following antisense sequences were chosen for silencing of mouse or human *Ebag9/EBAG9*:

5′-ATAACCGAAACTGAGTGATGG-3′ (miR-M1/m141),

5′-TTAAATAACCGAAACTGAGTG-3′ (miR-M2/m142),

5′-AAGTCCACTCCTCCACATCTG-3′ (miR-M3/m143),

5′-TGCTGAGTAGCCACATTCCCA-3′ (miR-M4/m144),

5′-TTGTTCTGCTGCTCTCTTCTC-3′ (miR-M5/m145).

5′-AAATAACCGAAACTGGGTGAT-3′ (miR-H17)

5′-TTAAATAACCGAAACTGGGTG-3′ (miR-H18)

Vectors encoding antisense sequences (miR-H17, miR-H18) chosen for silencing of human *EBAG9* will be made available on request, but we may require a completed Materials Transfer Agreement. They are subject to a patent application (PCT/EP2020/058355).

miRNAs of about 150 bp were generated by overlap polymerase chain reaction (PCR) using a miR-155 template plasmid and synthesized DNA oligos encoding the antisense and sense sequences. First, the 5′ arm and the 3′ arm of the hairpin were generated using the primer pairs FWD-1 miR155/REV-1 miR-xx and the FWD-2 miR-xx/REV-2 miR155, respectively. The purified PCR products were annealed via the loop sequence and the full-length miRNAs were amplified in a second PCR using the primer pair FWD-1 miR155/REV-2 miR155. The final product was digested with BssHII and NsiI restriction endonucleases and cloned into an MP71-GFP vector with MluI and NsiI sites in the 5′ intron.

GFP was exchanged against anti-human SP6, BCMA, or CD19 CAR, but NotI- and EcoRI-mediated restriction digest. All cloning products were verified by sequencing.

Production of retroviral supernatant

Plat-E cells were used for the production of ecotropic retroviral particles containing the MP71 vector encoding for mouse *Ebag9*-targeting miRNAs and eGFP. The 293T-based retroviral packaging cell line Plat-E stably expresses the murine leukemia virus (MLV)-derived *gag*/*pol* and *env* genes and was further transiently transfected using the standard calcium phosphate precipitation method.[52]

Amphotropic retroviral supernatants were either produced by transient transfection of HEK-293T cells, or by using stable packaging cell lines based on 293Vec-Galv.[53] Transient transfection of HEK-293T cells was performed using the appropriate MP71 construct and two plasmids encoding the MLV *env* (pALF-10A1GaV) and *gag*/*pol* (pcDNA3.1-MLV gag/pol) genes in a 1:1:1 ratio. Retroviral supernatants were collected 48 h post transfection, filtered, and either used directly for transduction or stored at −80°C.

Retroviral transduction of primary mouse T cells

Primary mouse splenocytes were transduced 24 h after activation. To this end, a 24-well non-tissue culture plate was incubated overnight at 4°C with 12.5 μg/mL RetroNectin (TaKaRa). Thereafter, 500 μL of virus supernatant per well was transferred and centrifuged for 90 min at $3000 \times g$ at 4°C. The supernatants were discarded and 1×10^6 activated splenocytes, mouse TActivator CD3/28 beads (Thermo Fisher Scientific), 10 IU/mL rmIL2, and 4 μg/mL protamine sulfate (Sigma-Aldrich) were transferred per well into the plates. After adding further retroviral

supernatant (1:4 diluted in mTCM), cells were centrifuged for 20 min at 800 × *g* at 32°C and cultured overnight. One day after transduction, positively transduced cells were sorted by FACS.

Retroviral transduction of primary human T cells

Transduction of human T cells with CAR-encoding retroviruses was done essentially as described.[25] Briefly, activated human T cells were subjected to two rounds of transduction starting 48 h after T cell activation. Retroviral supernatant was transferred to a RetroNectin-coated 24-well, non-tissue-treated plate and centrifuged for 90 min at 3,000 × *g* and 4°C. Next, 1 mL of activated T cells supplemented with the respective cytokines and protamine sulfate were transferred to the virus-coated plate. After adding further virus supernatant, cells were spinoculated at 800 × *g* at 32°C for 20 min and cultured overnight. On the next day, 1 mL of medium was removed and the transduction procedure was repeated. Cell culture medium supplemented with the respective cytokines was added to the cells as required. On day 13 after T cell activation (prior to functional assays 48 h later), medium was exchanged for fresh hTCM containing 10 IU/mL rhIL-2 and 1 ng/mL rhIL15 (for *in vitro* cytotoxicity, degranulation assay, granzyme A release, and cytokine secretion assay), or rhIL-7 and rhIL15 at a concentration of 10 ng/mL each (for transcriptome analysis, single-molecule fluorescence microscopy, *in vitro* repetitive antigen stimulation, and murine xenotransplantation model).

Real-time PCR analysis

RNA from flow cytometry-sorted positively transduced T cells was extracted using the RNeasy Mini Kit including the RNase-free DNase Set (Quiagen). RNA was transcribed into cDNA employing the SuperScript III First-Strand Synthesis SuperMix for qRT-PCR (Thermo Fisher Scientific). Quantitative real-time PCR analysis was performed using a TaqMan-based expression assay (Applied Biosystems). Reactions were performed in triplicates using the Applied Biosystems StepOnePlus Real-Time PCR system. Results were analyzed using the StepOne software (v2.3). The expression of a gene of interest (GOI) was calculated relative to the expression of *Gapdh*/*GAPDH* for calculation of *Ebag9* levels. For genes involved in metabolism, *SDHA* was used as a housekeeping gene. A list of all TaqMan primers is presented in supplemental information, reagents table.

Transcriptome analysis

CD3+ T cells were isolated from peripheral blood of healthy donors. CAR T cells were cultured and transduced in the presence of rhIL-7 and rhIL-15 (10 ng/mL each). T cells at day 10 of culture were stained for viable CAR-expressing cells (7-AAD−IgG+) and 1-3 × 10^6 cells were sorted in RLT lysis buffer (Qiagen). Samples were frozen and stored at −80°C. RNA was extracted using the RNeasy Mini Kit Plus (Qiagen). To generate mRNA sequencing libraries, 500 ng total RNA were subjected to the TruSeq Stranded mRNA Library Prep Kit (Illumina). Samples were sequenced on a NovaSeq (Illumina).

RNA-seq analysis

The RNA-seq data has been mapped on the GENCODE version 31 transcriptome with decoys using salmon version 0.14.1.[54] The sequences of both MP71 vector constructs (BCMA CAR and miR-H18 BCMA CAR) have been added to the reference transcriptome. All the analysis was done using R version 3.6.1 and Bioconducter 3.9. The summary of mapping results at gene level was done using tximeta version 1.5.6.[55] In total, 25,147 genes were assayed. Normalization and differential analysis were carried out using DESeq2 version 1.24.0, after omitting 35,190 genes without counts in any sample. For differential analysis, a donor effect was added to the treatment effect in the model, and 15,625 genes out of 35,023 were omitted from the differential expression analysis after independent hypothesis filtering. From the remaining genes, 4,106 were deemed differentially expressed, at a false discovery rate (FDR) threshold of 0.05, computed after independent hypothesis weighting from the IHW package version 1.12.0.[56] Apeglm shrinkage[57] was applied to the log2-fold changes.

The GENCODE gene IDs were mapped to HUGO Gene Nomenclature Committee (HGNC) symbols using the org.Hs.eg.db Bioconductor version 3.8.2, resulting in 15,423 unique HGNC gene IDs. Differential expression was carried out using GENCODE gene IDs, but, for the figures based on HGNC symbols, the highest expression value in each sample was selected when multiple GENECODE gene IDs corresponded to the same HGNC symbol. For the over-representation and GSEA, only genes that could be mapped to HGNC symbols were used. GSEA was performed using clusterProfiler version 3.12.0.[58]

Flow cytometry

Prior to antibody staining, mouse splenocytes were blocked with anti-mouse CD16/32 antibody in FACS buffer for 20 min on ice and washed once. Fc block for human cells was performed by adding 10% human AB serum. To discriminate between living and dead cells, stained samples were incubated with 7AAD (BioLegend) 5 to 10 min before data acquisition or stained with LIVE/DEADTM Fixable Aqua Dead Cell Stain Kit (Molecular Probes) prior to antibody staining.

The following fluorochrome-conjugated antibodies (if not otherwise stated, all from BioLegend) against mouse antigens were used for analysis by flow cytometry:

CD4 (GK1.5) and CD8a (53-6-7). Analytical samples were acquired on either a FACS Canto II flow cytometer (BD Biosciences), or a MACSQuant X analyzer (Miltenyi Biotec). The data were analyzed with FlowJo v. 10.0.8 software (Tree Star). All cell-sorting steps were carried out on a FACS Aria III or FACS Aria Fusion instrument (BD Biosciences).

Antibodies

Fluorochrome-conjugated antibodies (if not otherwise stated, all from BioLegend) against the following human antigens were used for analysis by flow cytometry: CD3 (HIT3a), CD4 (OKT4 and RPA-T4), CD8a (HIT8a), CD8 (SK1), CD19 (HIB19), CD45RA (HI100), CD45RO

(UCHL1), CD62L (DREG-56), CD107a/LAMP-1 (H4A3, BD Biosciences), CD138 (MI35), CD197/CCR7 (G043H7), CD223/LAG-3 (11C3C65), CD269/BCMA (19F2), CD279/PD-1 (EH12.2H7), CD366/TIM-3 (F38-2E2), and IgG (polyclonal, Southern Biotech).

For detection of NY-ESO-1 TCR, a fluorescein isothiocyanate (FITC) anti-mouse TCRβ antibody (H57-597) was used. Intracellular granzyme A (CB9) staining was done using the FIX&PERM Cell Permeabilization Kit (Molecular Probes).

Immunoblotting

Lysates of sorted GFP+, CAR +, or TCRβ+ cells were generated in radioimmunoprecipitation assay (RIPA) buffer supplemented with phenylmethysulfonyl fluoride (PMSF, 1 mM) and aprotinin (5 μg/mL). Lysates were analyzed by denaturing SDS-PAGE and transferred onto a nitrocellulose membrane (GE Healthcare). The following primary antibodies were used: anti-EBAG9 (polyclonal rabbit, in-house)[59] and anti-calnexin (polyclonal goat, Enzo Life Sciences).

Granzyme A release assay

Human CD8+ CAR T cells and non-transduced T cells were resuspended in FCS-free medium containing 1% BSA at a density of 2×10^6 cells/mL. The granzyme A release was stimulated by transferring 2×10^5 cells per well on a 96-well flat-bottom plate coated with anti-human CD3 (5 μg/mL) and anti-human CD28 (1 μg/mL). All samples were performed in triplicates. After incubation for 4 h at 37°C, supernatants were frozen at −20°C or analyzed immediately. To determine the total enzymatic activity, cells were lysed in 1% Triton X-100 and incubated for 1 h at 4°C. To calculate granzyme A activity, the enzymatic reaction of the substrate 0.2 mM N^{α}benzyloxycarbonyl-L-lysine thiobenzyl ester (BLT, Merck Millipore) in the presence of 0.2 mM 5,5′-dithio-*bis*-(2-nitrobenzoic acid) (DNBT, Sigma-Aldrich) was analyzed. Product concentration was measured at 405 nm and correlates with enzymatic activity.

Cytokine secretion assay

Antigen-stimulated cytokine secretion by human CD8+ CAR T cells was performed exactly as described.[25] Briefly, T cells were co-cultured with tumor cell lines in a 1:1 ratio (5×10^4 cells per well each) in 96-well plates for 24 h. All samples were performed in duplicates. The concentrations of human IFN-γ, TNF-α, and IL-2 in culture supernatants were determined by enzyme-linked immunosorbent assays (ELISAs) according to the manufacturer's instructions (BD Bioscience). Antigen-independent maximal release was achieved by incubation of T cells with 1 μM ionomycin and 5 ng/mL phorbol-12-myristate-13-acetate (PMA). Minimum release represents T cells incubated without target cells.

In vitro cytotoxicity assay

Antigen-stimulated *in vitro* cytotoxicity of CD8+ CAR T cells was measured by [51Cr]-chromium release, essentially as described.[25] Target cells were labeled with 20 μCi [51Cr] sodium chromate (PerkinElmer) in hTCM (+15% FCS) for 90 min at 37°C. Thereafter, target cells were co-cultured with effector CAR T cells for 4 h at 37°C in different effector:target ratios (E:T). Assay supernatants were transferred to LUMA-scintillation plates, air dried, and counted for [51Cr]-chromium release by using a Top γ-Scintillation Count Reader (PerkinElmer). All samples were performed in duplicates. Target cell maximum release was determined by directly counting labeled cells. Spontaneous release was measured by incubating target cells alone. Calculation of specific lysis was achieved according to the formula:

$$\% \text{ lysis} = [(\text{experimental lysis} - \text{spontaneous lysis}) \times 100] \times / (\text{maximum lysis} - \text{spontaneous lysis}).$$

Real-time quantification of *in vitro* cytotoxicity

A 96-well flat-bottom plate was coated with 0.0001% poly-L-lysine in PBS for 2 h at 37°C. After washing the plate with PBS, GFP-expressing target cells (2×10^4 cells per well) and CAR T cells at an E:T ratio of 5:1 were seeded. Cells were allowed to settle for 2 h at 37°C before starting imaging in an IncuCyte system (Sartorius) every 30 min over a total time frame of 42 h "Green" object count ($1/mm^2$) was quantified and normalized to the first data point. Loss of GFP signal intensity was interpreted as killing of the GFP+ target cells. All samples were performed in triplicates.

Flow cytometry-based cytotoxicity assay

In vitro cytotoxicity of CD4+ and CD8+ CAR T cells was analyzed by flow cytometry. CD4+ and CD8+ T cells were magnetically separated after transduction at the end of cultivation and co-cultured in a 1:1 ratio with different BCMA-expressing target cell lines. After 72 h, dead cells were detected using LIVE/DEAD Fixable Aqua Dead Cell Stain Kit (Molecular Probes). Tumor cells were stained for CD19 or CD138. T cells were stained for CD3, CD4, and CD8. The BCMA-negative cell line REH was used as a negative control and the results were normalized to remaining REH cells in culture.

Degranulation assay

CAR T cells were co-cultured with target cells in a 96-well round-bottom plate in a 1:1 ratio (5×10^5 cells per well each) for 2 h at 37°C. Anti-human CD107a antibody (H4A3, BD Biosciences), as well as brefeldin A (10 μg/mL, Sigma-Aldrich) and Monensin/Golgi-Stop (0.7 μg/mL, BD Biosciences), was added directly to the co-culture. At the end of the stimulation period, cells were washed once in PBS and stained with anti-human CD4 (OKT4, BioLegend) and CD8 (SK1, BioLegend) antibodies. To distinguish the target cells, either GFP-expressing target cells or staining with an anti-human CD19 (HIB19, BioLegend) antibody was used. After surface marker staining, cells were fixed and permeabilized followed by intracellular staining with anti-human granzyme A antibody (CB9, BioLegend). Antigen-independent maximal release was achieved by incubation of T cells with ionomycin and PMA. Minimum release represents T cells incubated without target cells.

Single-molecule fluorescence microscopy

The extracellular domain of human BCMA protein (amino acids 4–54) carrying a C-terminal His_{12}-tag was recombinantly expressed

in *Escherichia coli* (Rosetta 2 T7) and purified by Ni-nitrilotriacetic acid (NTA) chromatography followed by size exclusion chromatography. Purified BCMA protein was labeled with Alexa Fluor 555 NHS Ester (succinimidyl ester) and excess dye from the labeling reaction was removed by performing a size exclusion chromatography. Supported lipid bilayers (SLBs) were prepared on microscopy chambers (#178599, PK Thermo Fischer Scientific), loaded with increasing amounts of BCMA-AlexaFluor (AF)555, 75 ng of unlabeled intercellular adhesion molecule 1 (ICAM-1), and 40 ng of unlabeled T cell co-stimulatory molecule (B7-1), both recombinantly expressed in insect cells. CAR T cells were loaded with Fura-2 dye (final concentration 5 μM) in culture medium at 37°C for 30 min. After Fura-2 loading, cells were washed once with 5 mL of imaging buffer (Hank's buffered salt solution [HBSS] supplemented with 2 mM $CaCl_2$ 2 mM $MgCl_2$, 2% FCS, and 10 mM HEPES) and stored on ice until imaging (for ≤30 min). SLBs were then washed once with imaging buffer, incubated at 37°C for 30 min, before Fura-2 loaded cells were seeded onto the SLBs and imaging was commenced at 37°C. As soon as the first cells touched the SLB, intracellular calcium levels were measured by recording Fura-2 dye's 510/80 nm emission resulting from excitation by 340- and 387-nm light every minute for 15 min, using a UV-transmissive 20× objective (HC PL FLUOTAR 20×/0.50 PH2 ∞/0.17/D, Leica). For determining the antigen densities and proportion of cells fluxing calcium, images were analyzed with custom-written software. More detailed methods regarding measuring antigen densities and intracellular calcium measurements are given in Gudipati et al.[60]

Functional analysis of TCR-engineered T cells with peptide-pulsed T2 cells

Before the functional analysis, the transduction rates of NY-ESO-1 TCR and miR-H18 NY-ESO-1 TCR-transduced CD8+ T cells were adjusted with non-transduced cells from the same donor.

For the IFN-γ secretion assay, 5×10^4 T2 cells per well were co-cultured with 5×10^4 TCRβ+ NY-ESO-1 TCR, miR-H18 NY-ESO-1 TCR, or non-transduced CD8+ T cells in 200 μL/well TCM and in the presence of the indicated concentrations of cognate NY-ESO-$1_{157\text{-}165}$ (SLLMWITQC acid) or control MAGE-A1 (KVLEYVIKV acid) peptides (Discovery Peptides, Billingham, UK) for 18 h in U-bottom 96-well plates (TPP, Trasadingen, Switzerland). All samples were performed in duplicates. IFN-γ was quantified in the supernatants by ELISA.

For the CytoTox 96 non-radioactive LDH cytotoxicity assay (Promega), 1×10^4 T2 cells per well were co-cultured with TCRβ+ NY-ESO-1 TCR, miR-H18 NY-ESO-1 TCR, or non-transduced CD8+ T cells in 100 μL/well LDH medium (RPMI-1640 phenol red [−], 5% FCS, 1% Pen/Strep, 1% NEAA, and 1% Na-Pyr) at the indicated E:T ratios and in the presence of the indicated concentrations of NY-ESO-1 or MAGE-A1 peptides in U-bottom 96-well plates. The plates were centrifuged at 250 × *g* for 4 min and incubated at 37°C for 4 h. All samples were performed in triplicates. LDH was quantified in the supernatants according to the manufacturer's instructions.

Repetitive antigen-stimulation assay

To analyze the effect of *in vitro* repetitive antigen stimulation on CAR T cells, a stress test was performed in a similar manner essentially as described.[25,30] CAR T cells were cultured and transduced in the presence of rhIL-7 and rhIL-15 (10 ng/mL each). On day 10–13 of culture, CAR T cells were co-cultivated in 24-well plates with MM.1S tumor cells at a 1:1 ratio (5×10^5 cells per well each), in the presence of 0.1 ng/mL rhIL-7 and rhIL-15. Supernatants were harvested to quantify the release of IFN-γ 72 h after co-cultivation. An aliquot of the cells was analyzed by flow cytometry using CD138 staining of MM.1S tumor and CD3 staining of T cells. CD138 MACS Micro Beads (Miltenyi Biotec) were used to deplete residual CD138+ MM.1S tumor cells. Enriched CAR T cells were then used for another round of co-culturing with MM.1S tumor cells. In total, five rounds of transfer over 15 days were performed. Total cell cultivation time of T cells amounted to 25–28 days. After each round, T cells were analyzed for cell numbers, viability, and CD4+/CD8+ subset distribution. In addition, CAR expression and T cell memory marker expression was assessed.

Heterotopic cardiac transplantation

Female C57.BL/6 (H-2b) WT and *Ebag9*$^{-/-}$ mice (at least 15 generations backcrossed) weighing 22–25 g were used as recipients, and male B6 mice were used as donors; syngeneic controls were male recipients. Recipient HY^- female mice were subjected to intra-abdominal minor histocompatibility antigen (miHAg)-mismatched cardiac transplantation using the hearts from HY+ male donors. Briefly, donor hearts were transplanted into the abdominal cavity of the recipients after a short period of cold ischemia. Donor aorta and pulmonary artery were anastomosed in an end-to-side fashion to the infrarenal aorta and vena cava as the inflow and outflow vessels, respectively. The grafts were monitored by daily palpation of heartbeats; animals were sacrificed on the day of rejection, as defined by cessation of beating.

In vivo cytotoxicity assay

Donor C57Bl/6 mice were immunized twice intraperitoneally (i.p.) with the SV40 large T antigen-expressing cell line Co16.113 prior to isolation and retroviral transduction of bulk splenocytes. Twenty-four hours after transduction, GFP-expressing transduced cells were sorted by FACS and transferred intravenously (i.v.) into *Rag2*$^{-/-}$ recipient mice. *Rag2*$^{-/-}$ mice were immunized i.p. on days 1 and 15 after T cell transfer with Co16.113 cells. *In vivo* killing assay was performed on day 19 after transfer. Splenocytes from untreated C57Bl/6 mice were isolated and resuspended in PBS at a density of 1×10^7 cells/mL. Peptide labeling was achieved by incubation of splenocytes with 4 μg/mL Tag peptide IV (VVYDFLKL, JPT Peptide Technologies) for 30 min at 37°C. Half of the cells were left without peptide. Cells were washed and labeled with different amounts of the fluorescence dye eFluor670 (eBioscience) for 10 min at 37°C. The peptide-loaded population was stained with 1 μM eFluor670, while the non-loaded population was stained with 0.1 μM eFluor670. After washing the cells, 2×10^7 stained peptide-loaded and non-loaded target cells at a ratio of 1:1 were injected i.v. into recipient *Rag2*$^{-/-}$ mice. Recipients were sacrificed 16 h after transfer. Splenocytes were isolated and analyzed by flow cytometry. As a

control, naive non-immunized mice were included in each assay (control ratio). To calculate the specific killing, the following formula was applied (low = without peptide, high = peptide-pulsed):

$$\% \text{ specific killing} = [1 - (\text{control ratio}/\text{experimental ratio})] \times 100.$$

$$\text{Ratio} = \% \text{ low eFluor670 peak}/\% \text{ high eFluor670 peak}.$$

MM xenotransplantation model

The human MM cell line MM.1S (0.8×10^7 to 1×10^7 cells) was injected i.v. into NSG mice (NOD.Cg-PrkdcscidIl2rgtm1 Wji/SzJ, Jackson ImmunoResearch Laboratories). The MM.1S cell line was transduced with a lentivirus encoding firefly luciferase in tandem with eGFP50. Therefore, tumor growth was monitored using luciferin (Biosynth) i.p. application and *in vivo* bioluminescence imaging (BLI; IVIS spectrum imaging system; Caliper Life Sciences), essentially as described.[25] On day 7 after tumor injection, CAR T cells were administered i.v., as specified in the figure legends. Tumor progression was monitored on days indicated in the figure legends and after T cell injection by measurement of bioluminescence signals. Mice were imaged for several exposure times, ranging between 1 and 150 s. Binning and exposure were adjusted to achieve maximum sensitivity without leading to image saturation. To analyze the bioluminescence signal flux for each mouse as average radiance (p/s/cm2/sr), the Living Image software version 4.5 (Caliper Life Sciences) was used. BLI showed tumor manifestations in the bone marrow and thorax, and thus signal intensity was measured in regions of interest that encompassed the entire body of each individual mouse.

Animals were sacrificed on days indicated in the figure legends. Tumor cells and remaining human CAR T cells were detected and analyzed in the bone marrow. To analyze bone marrow cells, femora were dissected and flushed with PBS. The cell suspension was applied to a 70-μm cell strainer, centrifuged (400 × *g*, 5 min, 4°C) and lysed with hypotonic Ammonium-Chloride-Potassium (ACK) erythrocyte lysis buffer. Subsequently, cells were analyzed by flow cytometry.

Statistics

All of the statistical details of experiments can be found in the figures, figure legends, results, and/or supplemental tables, including the statistical tests used, and the exact value of n (representing number of animals per sample and number of experimental replicates). Results are expressed as arithmetic means ± SEM if not otherwise stated. Values of $p < 0.05$ were considered statistically significant, as determined by the unpaired Mann-Whitney U test, the unpaired or paired Student's t test, a multiple t test (one per row), a Mantel-Cox test, or the Wilcoxon matched-pairs signed-rank test, where appropriate. For normality testing, a Shapiro-Wilk test was used. Analyses were performed using Prism (GraphPad Software, version 6.0, 9.0), R (version 3.6.1), or Bioconductor 3.9.

Study approval

All animal studies were conducted in compliance with the institutional guidelines of the MDC and approved by the Berlin State review board at the Landesamt für Gesundheit und Soziales, Berlin (registered under Landesamt für Gesundheit und Soziales TVV G0331/05, G0089/10, G0091/15, G0050/16).

The recruitment of voluntary blood donors was conducted according to the declaration of Helsinki and in accordance with local ethical guidelines. Ethics votes to obtain leukapheresis material and buffy coats were obtained from the ethics board, Charité-University Hospital Berlin (registered under EA2/216/18; EA1/003/17).

DATA AVAILABILITY

The RNA-seq data from this paper were deposited under ENA: PRJEB37843.

SUPPLEMENTAL INFORMATION

Supplemental information can be found online at https://doi.org/10.1016/j.ymthe.2022.07.009.

ACKNOWLEDGMENTS

We thank Kerstin Gerlach, Maria Zschummel, H.-P. Rahn, Kirstin Rautenberg, and Dennis Kobelt (all MDC) for expert technical assistance, and Julia Bluhm (MDC) for providing the BCMA CAR plasmid. The authors acknowledge the Genomics Platform and Sascha Sauer for RNA-seq (MDC).
This work was funded by grants from Deutsche Forschungsgemeinschaft (DFG, Germany), SFB/TR36 (project B12), and RE 1103/6-1; and from the MDC PreGoBio funding program (Germany; all to A.R. and U.E.H.).

AUTHOR CONTRIBUTIONS

A.R. and U.E.H. conceived and supervised the experiments, performed data analysis, and wrote the manuscript. A.W. performed most of the experiments, wrote the manuscript, and analyzed and discussed the data. M.B. designed miRNA-retrovirus constructs, and analyzed and discussed the data. V.G., J.J.J., and M.W.M. performed experiments and analyzed the data. E.B. performed RNA-seq bioinformatical analysis and wrote the manuscript. H.S. provided an essential cDNA construct. D.B. and J.B.H. analyzed and discussed the data and their interpretation. All authors reviewed the manuscript.

DECLARATION OF INTERESTS

A.W., M.B., U.E.H., and A.R. have filed patent applications on the BCMA CAR and on the therapeutic application of EBAG9 modulation, respectively. The authors have declared that no competing conflict of interests exist regarding data acquisition and interpretation. A.R. and U.E.H. received research funding from Fate Therapeutics (San Diego, CA) for work unrelated to the main topic in this manuscript.

REFERENCES

1. Brentjens, R.J., Davila, M.L., Riviere, I., Park, J., Wang, X., Cowell, L.G., Bartido, S., Stefanski, J., Taylor, C., Olszewska, M., et al. (2013). CD19-targeted T cells rapidly

induce molecular remissions in adults with chemotherapy-refractory acute lymphoblastic leukemia. Sci. Transl. Med. *5*, 177ra38.

2. Maude, S.L., Frey, N., Shaw, P.A., Aplenc, R., Barrett, D.M., Bunin, N.J., Chew, A., Gonzalez, V.E., Zheng, Z., Lacey, S.F., et al. (2014). Chimeric antigen receptor T cells for sustained remissions in leukemia. N. Engl. J. Med. *371*, 1507–1517.

3. Schuster, S.J., Svoboda, J., Chong, E.A., Nasta, S.D., Mato, A.R., Anak, Ö., Brogdon, J.L., Pruteanu-Malinici, I., Bhoj, V., Landsburg, D., et al. (2017). Chimeric antigen receptor T cells in refractory B-cell lymphomas. N. Engl. J. Med. *377*, 2545–2554.

4. Majzner, R.G., and Mackall, C.L. (2018). Tumor antigen escape from CAR T-cell therapy. Cancer Discov. *8*, 1219–1226.

5. Yu, H., Sotillo, E., Harrington, C., Wertheim, G., Paessler, M., Maude, S.L., Rheingold, S.R., Grupp, S.A., Thomas-Tikhonenko, A., and Pillai, V. (2017). Repeated loss of target surface antigen after immunotherapy in primary mediastinal large B cell lymphoma. Am. J. Hematol. *92*, E11–E13.

6. Brudno, J.N., Maric, I., Hartman, S.D., Rose, J.J., Wang, M., Lam, N., Stetler-Stevenson, M., Salem, D., Yuan, C., Pavletic, S., et al. (2018). T cells genetically modified to express an anti-B-cell maturation antigen chimeric antigen receptor cause remissions of poor-prognosis relapsed multiple myeloma. J. Clin. Oncol. *36*, 2267–2280.

7. Raje, N., Berdeja, J., Lin, Y., Siegel, D., Jagannath, S., Madduri, D., Liedtke, M., Rosenblatt, J., Maus, M.V., Turka, A., et al. (2019). Anti-BCMA CAR T-cell therapy bb2121 in relapsed or refractory multiple myeloma. N. Engl. J. Med. *380*, 1726–1737.

8. Cohen, A.D., Garfall, A.L., Stadtmauer, E.A., Melenhorst, J.J., Lacey, S.F., Lancaster, E., Vogl, D.T., Weiss, B.M., Dengel, K., Nelson, A., et al. (2019). B cell maturation antigen-specific CAR T cells are clinically active in multiple myeloma. J. Clin. Invest. *129*, 2210–2221.

9. Lim, W.A., and June, C.H. (2017). The principles of engineering immune cells to treat cancer. Cell *168*, 724–740.

10. Rafiq, S., Hackett, C.S., and Brentjens, R.J. (2020). Engineering strategies to overcome the current roadblocks in CAR T cell therapy. Nat. Rev. Clin. Oncol. *17*, 147–167.

11. Blankenstein, T., Coulie, P.G., Gilboa, E., and Jaffee, E.M. (2012). The determinants of tumour immunogenicity. Nat. Rev. Cancer *12*, 307–313.

12. Zhang, N., and Bevan, M.J. (2011). CD8(+) T cells: foot soldiers of the immune system. Immunity *35*, 161–168.

13. Rabinovich, G.A., Gabrilovich, D., and Sotomayor, E.M. (2007). Immunosuppressive strategies that are mediated by tumor cells. Annu. Rev. Immunol. *25*, 267–296.

14. Gajewski, T.F., Schreiber, H., and Fu, Y.X. (2013). Innate and adaptive immune cells in the tumor microenvironment. Nat. Immunol. *14*, 1014–1022.

15. Maus, M.V., and June, C.H. (2016). Making better chimeric antigen receptors for adoptive T-cell therapy. Clin. Cancer Res. *22*, 1875–1884.

16. Gattinoni, L., Klebanoff, C.A., Palmer, D.C., Wrzesinski, C., Kerstann, K., Yu, Z., Finkelstein, S.E., Theoret, M.R., Rosenberg, S.A., and Restifo, N.P. (2005). Acquisition of full effector function in vitro paradoxically impairs the in vivo antitumor efficacy of adoptively transferred CD8+ T cells. J. Clin. Invest. *115*, 1616–1626.

17. Alexander-Miller, M.A. (2005). High-avidity CD8+ T cells: optimal soldiers in the war against viruses and tumors. Immunol. Res. *31*, 13–24.

18. Jensen, M.C., and Riddell, S.R. (2014). Design and implementation of adoptive therapy with chimeric antigen receptor-modified T cells. Immunol. Rev. *257*, 127–144.

19. Ménasché, G., and de Saint Basile, G. (2009). EBAG9 tempers lymphocyte killing activity. J. Clin. Invest. *119*, 2136–2140.

20. Rüder, C., Höpken, U.E., Wolf, J., Mittrücker, H.W., Engels, B., Erdmann, B., Wollenzin, S., Uckert, W., Dörken, B., and Rehm, A. (2009). The tumor-associated antigen EBAG9 negatively regulates the cytolytic capacity of mouse CD8+ T cells. J. Clin. Invest. *119*, 2184–2203.

21. Miyazaki, T., Ikeda, K., Horie-Inoue, K., Kondo, T., Takahashi, S., and Inoue, S. (2014). EBAG9 modulates host immune defense against tumor formation and metastasis by regulating cytotoxic activity of T lymphocytes. Oncogenesis *3*, e126.

22. Rehm, A., Wirges, A., Hoser, D., Fischer, C., Herda, S., Gerlach, K., Sauer, S., Williomsky, G., and Höpken, U.E. (2022). EBAG9 controls CD8+ T cell memory formation responding to tumor challenge in mice. JCI Insight *7*, e155534.

23. Bleakley, M., and Riddell, S.R. (2011). Exploiting T cells specific for human minor histocompatibility antigens for therapy of leukemia. Immunol. Cell Biol. *89*, 396–407.

24. Bunse, M., Bendle, G.M., Linnemann, C., Bies, L., Schulz, S., Schumacher, T.N., and Uckert, W. (2014). RNAi-mediated TCR knockdown prevents autoimmunity in mice caused by mixed TCR dimers following TCR gene transfer. Mol. Ther. *22*, 1983–1991.

25. Bluhm, J., Kieback, E., Marino, S.F., Oden, F., Westermann, J., Chmielewski, M., Abken, H., Uckert, W., Höpken, U.E., and Rehm, A. (2018). CAR T cells with enhanced sensitivity to B cell maturation antigen for the targeting of B cell non-hodgkin's lymphoma and multiple myeloma. Mol. Ther. *26*, 1906–1920.

26. Brudno, J.N., and Kochenderfer, J.N. (2016). Toxicities of chimeric antigen receptor T cells: recognition and management. Blood *127*, 3321–3330.

27. Wherry, E.J., and Kurachi, M. (2015). Molecular and cellular insights into T cell exhaustion. Nat. Rev. Immunol. *15*, 486–499.

28. Guy, C., Mitrea, D.M., Chou, P.C., Temirov, J., Vignali, K.M., Liu, X., Zhang, H., Kriwacki, R., Bruchez, M.P., Watkins, S.C., et al. (2022). LAG3 associates with TCR-CD3 complexes and suppresses signaling by driving co-receptor-Lck dissociation. Nat. Immunol. *23*, 757–767.

29. Gong, W., Hoffmann, J.M., Stock, S., Wang, L., Liu, Y., Schubert, M.L., Neuber, B., Hückelhoven-Krauss, A., Gern, U., Schmitt, A., et al. (2019). Comparison of IL-2 vs IL-7/IL-15 for the generation of NY-ESO-1-specific T cells. Cancer Immunol. Immunother. *68*, 1195–1209.

30. Künkele, A., Johnson, A.J., Rolczynski, L.S., Chang, C.A., Hoglund, V., Kelly-Spratt, K.S., and Jensen, M.C. (2015). Functional tuning of CARs reveals signaling threshold above which CD8+ CTL antitumor potency is attenuated due to cell fas-FasL-dependent AICD. Cancer Immunol. Res. *3*, 368–379.

31. Boyle, E.I., Weng, S., Gollub, J., Jin, H., Botstein, D., Cherry, J.M., and Sherlock, G. (2004). GO::TermFinder–open source software for accessing Gene Ontology information and finding significantly enriched Gene Ontology terms associated with a list of genes. Bioinformatics *20*, 3710–3715.

32. Subramanian, A., Tamayo, P., Mootha, V.K., Mukherjee, S., Ebert, B.L., Gillette, M.A., Paulovich, A., Pomeroy, S.L., Golub, T.R., Lander, E.S., and Mesirov, J.P. (2005). Gene set enrichment analysis: a knowledge-based approach for interpreting genome-wide expression profiles. Proc. Natl. Acad. Sci. USA *102*, 15545–15550.

33. Liberzon, A., Birger, C., Thorvaldsdóttir, H., Ghandi, M., Mesirov, J.P., and Tamayo, P. (2015). The Molecular Signatures Database (MSigDB) hallmark gene set collection. Cell Syst. *1*, 417–425.

34. Vogelstein, B., Papadopoulos, N., Velculescu, V.E., Zhou, S., Diaz, L.A., Jr., and Kinzler, K.W. (2013). Cancer genome landscapes. Science *339*, 1546–1558.

35. Ali, N., Flutter, B., Sanchez Rodriguez, R., Sharif-Paghaleh, E., Barber, L.D., Lombardi, G., and Nestle, F.O. (2012). Xenogeneic graft-versus-host-disease in NOD-scid IL-2Rgammanull mice display a T-effector memory phenotype. PLoS One *7*, e44219.

36. Teachey, D.T., Lacey, S.F., Shaw, P.A., Melenhorst, J.J., Maude, S.L., Frey, N., Pequignot, E., Gonzalez, V.E., Chen, F., Finklestein, J., et al. (2016). Identification of predictive biomarkers for cytokine release syndrome after chimeric antigen receptor T-cell therapy for acute lymphoblastic leukemia. Cancer Discov. *6*, 664–679.

37. Walker, A.J., Majzner, R.G., Zhang, L., Wanhainen, K., Long, A.H., Nguyen, S.M., Lopomo, P., Vigny, M., Fry, T.J., Orentas, R.J., and Mackall, C.L. (2017). Tumor antigen and receptor densities regulate efficacy of a chimeric antigen receptor targeting anaplastic lymphoma kinase. Mol. Ther. *25*, 2189–2201.

38. Morgan, R.A., Yang, J.C., Kitano, M., Dudley, M.E., Laurencot, C.M., and Rosenberg, S.A. (2010). Case report of a serious adverse event following the administration of T cells transduced with a chimeric antigen receptor recognizing ERBB2. Mol. Ther. *18*, 843–851.

39. Chen, J., López-Moyado, I.F., Seo, H., Lio, C.W.J., Hempleman, L.J., Sekiya, T., Yoshimura, A., Scott-Browne, J.P., and Rao, A. (2019). NR4A transcription factors limit CAR T cell function in solid tumours. Nature *567*, 530–534.

40. Scott, A.C., Dündar, F., Zumbo, P., Chandran, S.S., Klebanoff, C.A., Shakiba, M., Trivedi, P., Menocal, L., Appleby, H., Camara, S., et al. (2019). TOX is a critical regulator of tumour-specific T cell differentiation. Nature *571*, 270–274.

41. Dong, M.B., Wang, G., Chow, R.D., Ye, L., Zhu, L., Dai, X., Park, J.J., Kim, H.R., Errami, Y., Guzman, C.D., et al. (2019). Systematic immunotherapy target

Discovery using genome-scale in vivo CRISPR screens in CD8 T cells. Cell *178*, 1189–1204.e23.

42. Nguyen, L.T., and Ohashi, P.S. (2015). Clinical blockade of PD1 and LAG3–potential mechanisms of action. Nat. Rev. Immunol. *15*, 45–56.

43. Gubser, P.M., Bantug, G.R., Razik, L., Fischer, M., Dimeloe, S., Hoenger, G., Durovic, B., Jauch, A., and Hess, C. (2013). Rapid effector function of memory CD8+ T cells requires an immediate-early glycolytic switch. Nat. Immunol. *14*, 1064–1072.

44. Kawalekar, O.U., O'Connor, R.S., Fraietta, J.A., Guo, L., McGettigan, S.E., Posey, A.D., Jr., Patel, P.R., Guedan, S., Scholler, J., Keith, B., et al. (2016). Distinct signaling of coreceptors regulates specific metabolism pathways and impacts memory development in CAR T cells. Immunity *44*, 380–390.

45. Zhang, B., Karrison, T., Rowley, D.A., and Schreiber, H. (2008). IFN-gamma- and TNF-dependent bystander eradication of antigen-loss variants in established mouse cancers. J. Clin. Invest. *118*, 1398–1404.

46. Van Arnam, J.S., Lim, M.S., and Elenitoba-Johnson, K.S.J. (2018). Novel insights into the pathogenesis of T-cell lymphomas. Blood *131*, 2320–2330.

47. Girardi, T., Vicente, C., Cools, J., and De Keersmaecker, K. (2017). The genetics and molecular biology of T-ALL. Blood *129*, 1113–1123.

48. Marcucci, K.T., Jadlowsky, J.K., Hwang, W.T., Suhoski-Davis, M., Gonzalez, V.E., Kulikovskaya, I., Gupta, M., Lacey, S.F., Plesa, G., Chew, A., et al. (2018). Retroviral and lentiviral safety analysis of gene-modified T cell products and infused HIV and oncology patients. Mol. Ther. *26*, 269–279.

49. Stadtmauer, E.A., Fraietta, J.A., Davis, M.M., Cohen, A.D., Weber, K.L., Lancaster, E., Mangan, P.A., Kulikovskaya, I., Gupta, M., Chen, F., et al. (2020). CRISPR-engineered T cells in patients with refractory cancer. Science *367*, eaba7365.

50. Yanamandra, U., and Kumar, S.K. (2018). Minimal residual disease analysis in myeloma - when, why and where. Leuk. Lymphoma *59*, 1772–1784.

51. Oden, F., Marino, S.F., Brand, J., Scheu, S., Kriegel, C., Olal, D., Takvorian, A., Westermann, J., Yilmaz, B., Hinz, M., et al. (2015). Potent anti-tumor response by targeting B cell maturation antigen (BCMA) in a mouse model of multiple myeloma. Mol. Oncol. *9*, 1348–1358.

52. Morita, S., Kojima, T., and Kitamura, T. (2000). Plat-E: an efficient and stable system for transient packaging of retroviruses. Gene Ther. *7*, 1063–1066.

53. Hennig, K., Raasch, L., Kolbe, C., Weidner, S., Leisegang, M., Uckert, W., Titeux, M., Hovnanian, A., Kuehlcke, K., and Loew, R. (2014). HEK293-based production platform for gamma-retroviral (self-inactivating) vectors: application for safe and efficient transfer of COL7A1 cDNA. Hum. Gene Ther. Clin. Dev. *25*, 218–228.

54. Patro, R., Duggal, G., Love, M.I., Irizarry, R.A., and Kingsford, C. (2017). Salmon provides fast and bias-aware quantification of transcript expression. Nat. Methods *14*, 417–419.

55. Love, M.I., Soneson, C., Hickey, P.F., Johnson, L.K., Pierce, N.T., Shepherd, L., Morgan, M., and Patro, R. (2020). Tximeta: reference sequence checksums for provenance identification in RNA-seq. PLoS Comput. Biol. *16*, e1007664.

56. Ignatiadis, N., Klaus, B., Zaugg, J.B., and Huber, W. (2016). Data-driven hypothesis weighting increases detection power in genome-scale multiple testing. Nat. Methods *13*, 577–580.

57. Zhu, A., Ibrahim, J.G., and Love, M.I. (2019). Heavy-tailed prior distributions for sequence count data: removing the noise and preserving large differences. Bioinformatics *35*, 2084–2092.

58. Yu, G., Wang, L.G., Han, Y., and He, Q.Y. (2012). clusterProfiler: an R package for comparing biological themes among gene clusters. OMICS *16*, 284–287.

59. Engelsberg, A., Hermosilla, R., Karsten, U., Schülein, R., Dörken, B., and Rehm, A. (2003). The Golgi protein RCAS1 controls cell surface expression of tumor-associated O-linked glycan antigens. J. Biol. Chem. *278*, 22998–23007.

60. Gudipati, V., Rydzek, J., Doel-Perez, I., Gonçalves, V.D.R., Scharf, L., Königsberger, S., Lobner, E., Kunert, R., Einsele, H., Stockinger, H., et al. (2020). Inefficient CAR-proximal signaling blunts antigen sensitivity. Nat. Immunol. *21*, 848–856.

YMTHE, Volume *30*

Supplemental Information

EBAG9 silencing exerts an immune checkpoint function without aggravating adverse effects

Anthea Wirges, Mario Bunse, Jara J. Joedicke, Eric Blanc, Venugopal Gudipati, Michael W. Moles, Hiroshi Shiku, Dieter Beule, Johannes B. Huppa, Uta E. Höpken, and Armin Rehm

Supplemental information

Inventory of Supplemental information:

Supplementary Figures 1-10 and Figure legends

Supplementary Table S1, S2; Legends

Supplementary Methods, Reagents Table

Supplementary Figures and Figure legends

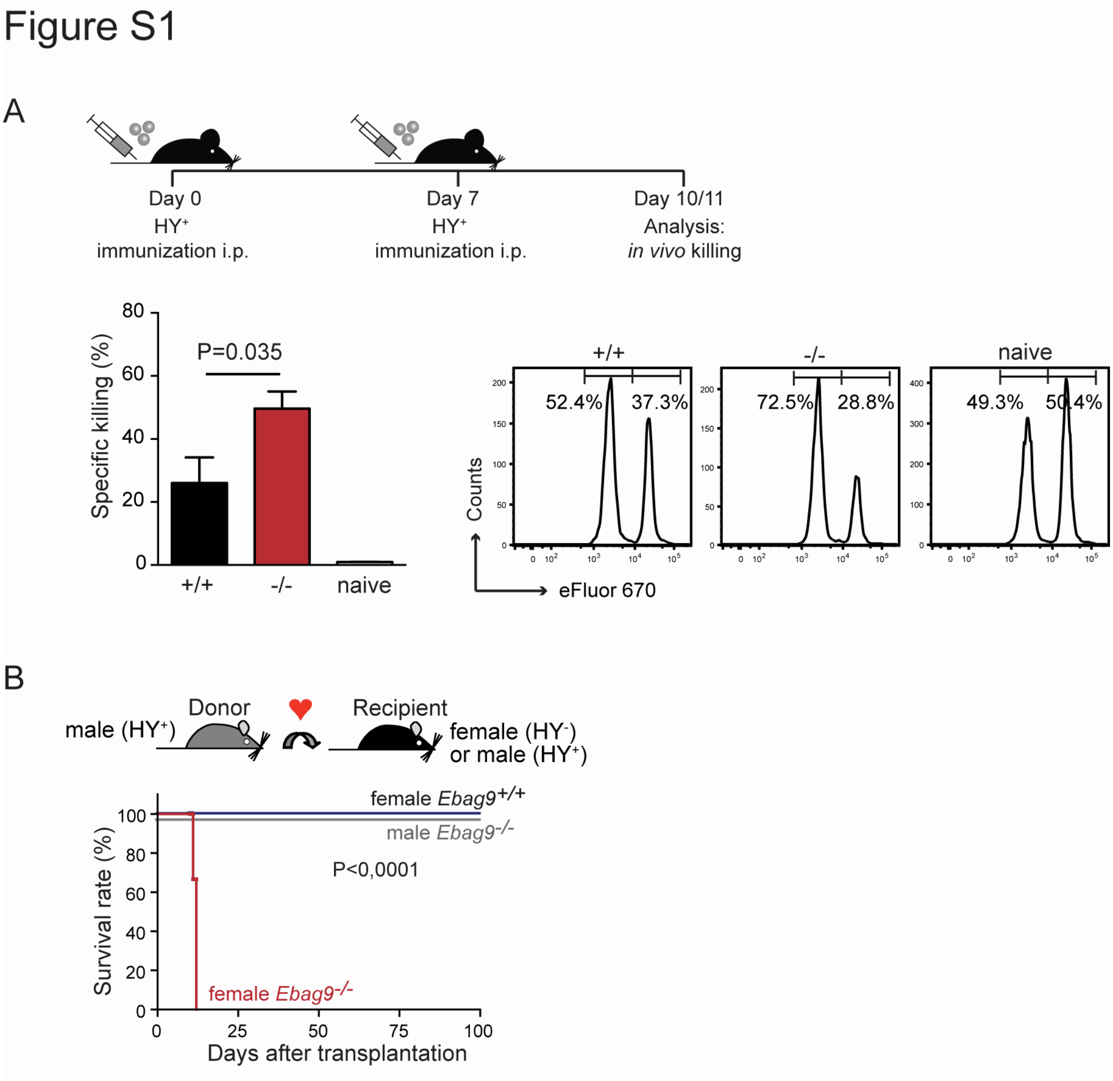

Figure S1: Enhanced anti-HY specific cytotoxic response of *Ebag9*$^{-/-}$ mice

(A) Female C57.BL/6 Wt (+/+) and *Ebag9*$^{-/-}$ (-/-) mice were immunized twice i.p. with $5x10^6$ male splenocytes (B6). At day 10, animals were challenged with equal numbers of female (HY^-) and male (HY^+) splenocytes labeled with different amounts of eFluor 670 (female low eFluor 670 and male high eFluor 670). The ratio between the two target populations was determined by flow cytometry 16 hrs later and expressed as specific killing of male splenocytes in percent. Naive, non-immunized mice were used as control (control ratio). Bars show mean values ± SEM of n=2 independent assays. One representative example is shown on the right (Wt, n=8; *Ebag9*$^{-/-}$, n=7; naive, non-immunized C57.BL/6 mice, n=3). Percentages of HY^- and HY^+ splenocytes are indicated. An unpaired Student's t-test was employed. To calculate the specific killing, the following formula was applied:

% specific killing = [1 - (control ratio / experimental ratio)] x 100

Ratio = % low eFluor 670 peak / % high eFluor 670 peak

(Low = w/o peptide, high = peptide-pulsed)

(B) Female C57.BL/6 Wt (+/+) or *Ebag9*$^{-/-}$ (-/-) mice were heterotopically transplanted with male donor hearts (C57.BL/6, Wt). Male cardiac allografts were acutely rejected in *Ebag9*$^{-/-}$ recipients at mean survival times of 12 ± 2 days (n=3), Wt mice (n=4) accepted the grafts over the observation period of 100 days. Syngeneic male heart transplants showed no rejection in male recipients. n=2 independent experiments, p values were determined with log-rank test (Mantel-Cox test).

Figure S2

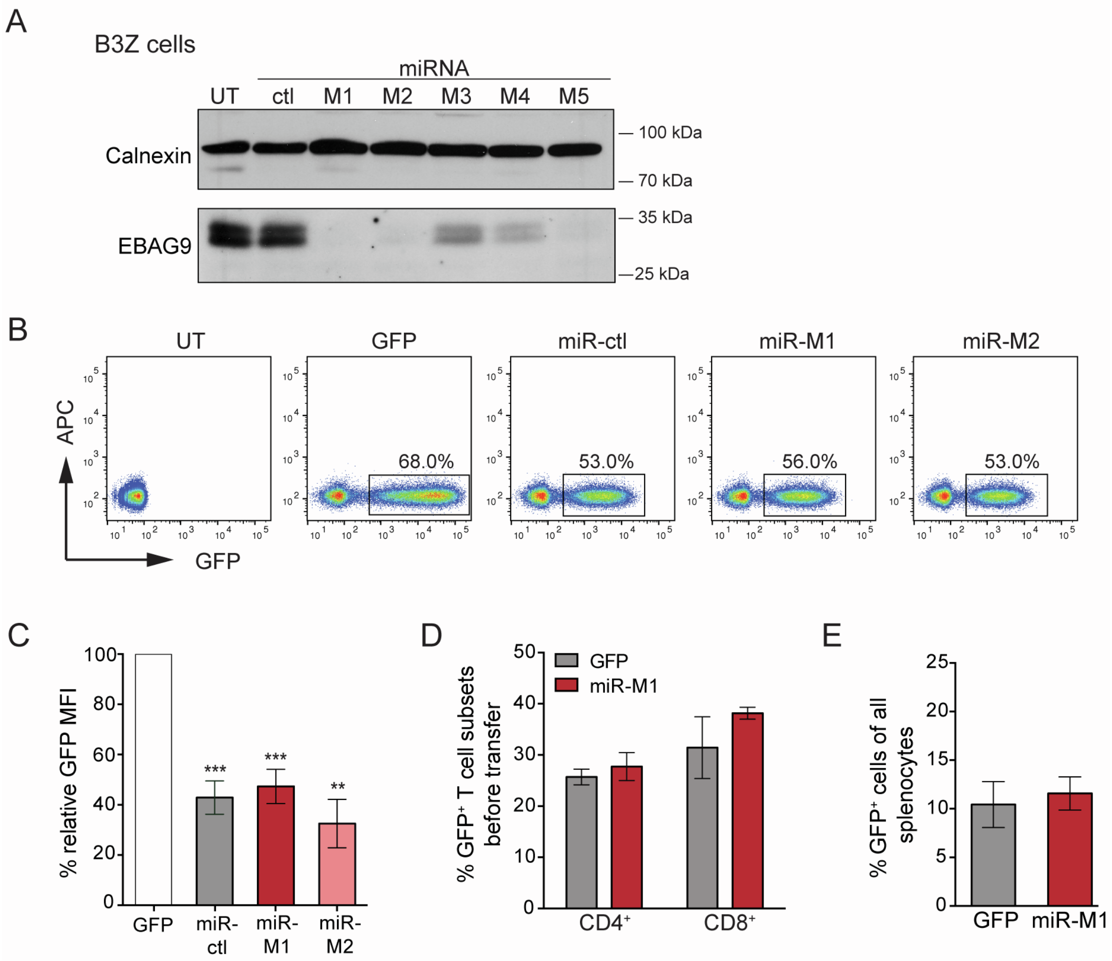

Figure S2: Efficacy of miRNA-mediated EBAG9 silencing in mouse T cells

(A) Target site validation for miRNA-mediated silencing of EBAG9. B3Z cells were retrovirally transduced with MP71 vectors encoding GFP and various miRNAs targeting different regions of the *Ebag9* transcript (miR-M1 – M5). Non-transduced (UT) cells and the miR-ctl without any endogenous target sequence were used as controls. GFP^{+} cells were sorted by FACS and analyzed by Western blot using rabbit anti-EBAG9 antibody. Calnexin served as a loading control.

(B) Efficient transduction of primary mouse T cells. Flow cytometric analysis of retrovirally transduced mouse T cells at day 6 after activation. Positively transduced cells express GFP. Transduction rates are indicated as numbers in the gate.

(C) Transduction of primary mouse T cells with vectors carrying GFP and the miRNAs led to reduced GFP expression levels. MFI is depicted as compared to GFP alone. Bars represent mean values ± SEM of n=7 experiments with n=4 (miR-M2) and n=8 (GFP, miR-ctl, -M1) samples per group. **p<0.01, *p<0.001. A one sample t-test was applied.

(D) Transduced mouse T cells (GFP^{+}) were analyzed by flow cytometry. GFP^{+} cells were co-stained for CD4^{+} and CD8^{+}. Bars represent mean values ± SEM of n=3 experiments with n=3 samples per group.

(E) Twenty days after T cell transfer into *Rag2*$^{-/-}$ mice, mice were sacrificed. T cells were analyzed by flow cytometry. Bars represent mean values ± SEM of n=5 experiments with n=10 (GFP) and n=15 (miR-M1) mice per group. In D) and E), a Mann-Whitney U test was applied. P-values were not significant.

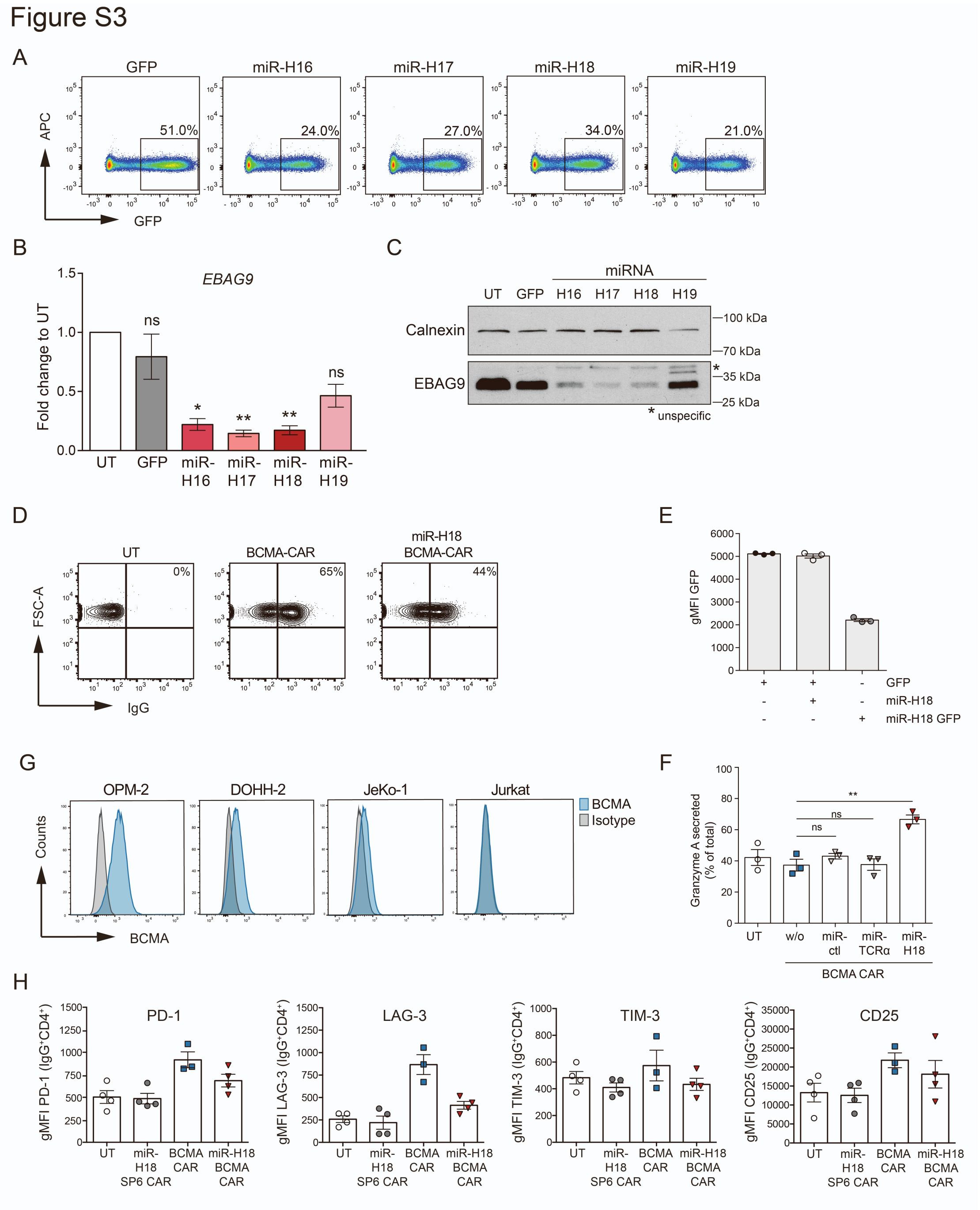

Figure S3: Efficacy of miRNA-mediated EBAG9 silencing in human T cells

(A) Retroviral transduction of human Jurkat cells with different GFP-encoding MP71 vectors expressing various miRNAs directed against human EBAG9. GFP expression was measured by flow cytometry. Transduction rates are indicated by numbers in the gates.

(B) miRNA-mediated reduction of human *EBAG9* mRNA. Jurkat T-ALL cells were retrovirally transduced with vectors encoding for GFP and various miRNAs targeting the *EBAG9* transcript (miR-H16 – H19). Transduced GFP^+ T cells were sorted by FACS and *EBAG9* mRNA expression was analyzed by RT-qPCR. Non-transduced (UT) cells were set arbitrarily at 1. Bars represent mean values ± SEM of n=2 experiments with n=3 samples per group. *p<0.05, **p<0.01; ns, not significant. A one-sample t-test was applied.

(C) miRNA-mediated reduction of EBAG9 protein level. Transduced T cells were raised as in (B). GFP^+ T cells were sorted by FACS and analyzed by Western blot using a rabbit anti-EBAG9 antibody. One representative Western blot out of three experiments is shown. Calnexin served as a loading control.

(D) Frequency and CAR surface expression levels of T cells ($CD3^+$) at day 7 after retroviral transduction. Gates were set relative to anti-IgG reactivity on untransduced (UT) T cells, gMFI values of cells in the IgG^+ gated population were determined using FloJo software. This FACS contour plot corresponds to Figure 2D).

(E) Reduced transgene expression is caused by the retroviral vector design carrying the miRNA intronically. Jurkat cells were transduced with GFP as indicated. Positively transduced cells were then sorted for GFP-expression and further transduced with a control vector or a miR-H18 containing vector. One group received miR-H18 GFP only. Bars represent mean values ± SEM of one experiment with n=3 samples per group.

(F) Control miRNAs have no impact on the release of granzyme A. Granzyme A release was induced by re-stimulation of $CD8^+$ BCMA CAR CAR T cells with anti-human CD3 and CD28 antibodies for 4 hrs. Enzymatic activities in the supernatant were measured in duplicates. Values show the release in percentages relative to the

total content. Bars represent mean ± SEM of one experiments with n=3 independent donors per group. **p<0.01; ns, not significant. A one-sample t-test was applied.

(G) Flow cytometric analysis of BCMA surface expression on MM (OPM-2) and B-NHL (DOHH-2, JeKo-1) cell lines. A representative histogram for each cell line is shown. The BCMA-negative Jurkat cell line served as a control.

(H) Quantification of flow cytometric analysis of key immune markers for T cell function. $CD4^+$ CAR T cells (IgG^+ $CD4^+$) at day 7 after activation were co-stained with anti-PD-1, anti-LAG-3, anti-TIM-3, and anti-CD25 antibodies. Bars represent mean ± SEM of one experiment with n=3 (BCMA CAR) and n=4 (UT, miR-H18 SP6 CAR, miR-H18 BCMA CAR) independent donors per group.

Figure S4

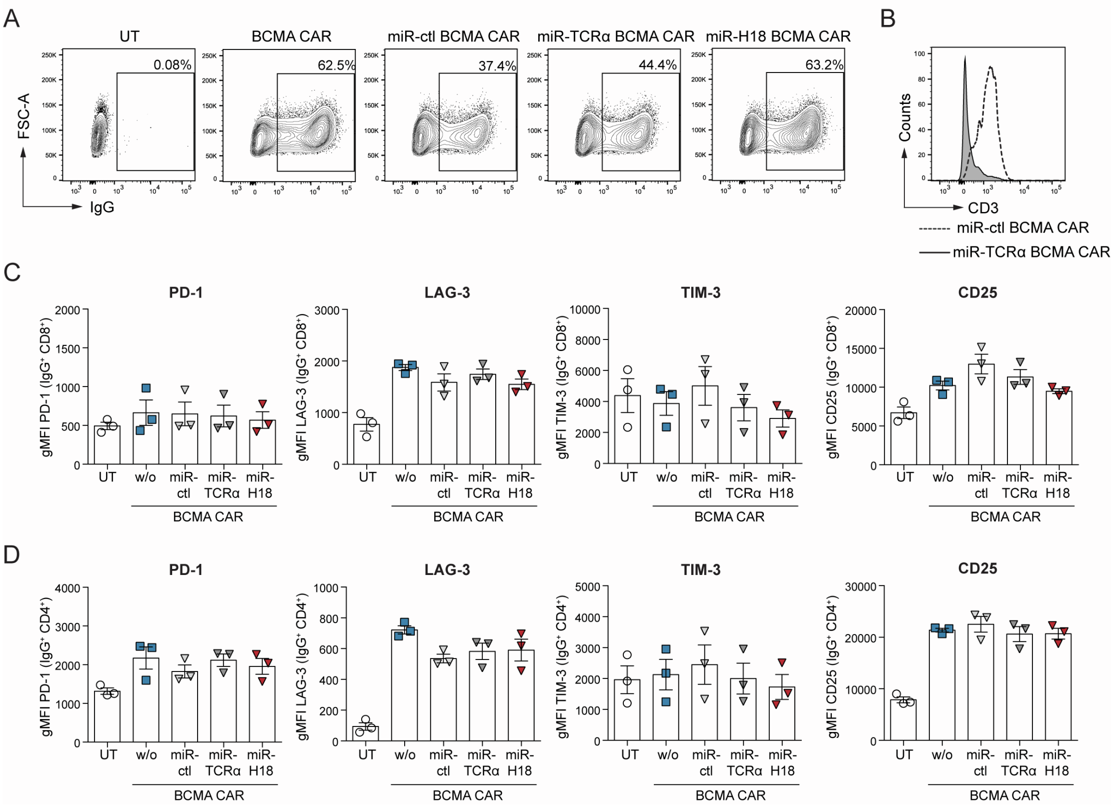

Figure S4: Phenotypic characterization of human BCMA CAR T cells expressing various miRNAs

(A) Retroviral transduction of primary human T cells with BCMA CAR-encoding MP71 vectors expressing various miRNAs directed against TCRα-chain (miR-TCRα), EBAG9 (miR-H18), or without any endogenous target (miR-ctl). Transduction rates are indicated by numbers in the gates.

(B) Efficient miR-TCRα-mediated downregulation of CD3. A representative histogram of flow cytometric analysis of CD3 surface expression on transduced T cells is shown. The miR-ctl served as a control.

(C) Quantification of flow cytometric analysis of key immune markers for T cell function. $CD8^+$ CAR T cells (IgG^+ $CD8^+$) at day 7 after activation were co-stained with anti-PD-1, anti-LAG-3, anti-TIM-3, and anti-CD25 antibodies.

(D) Quantification of flow cytometric analysis of key immune markers for $CD4^+$ CAR T cells at day 7 after activation. C) and D), bars represent mean ± SEM of one experiment with n=3 independent donors per group.

Figure S5

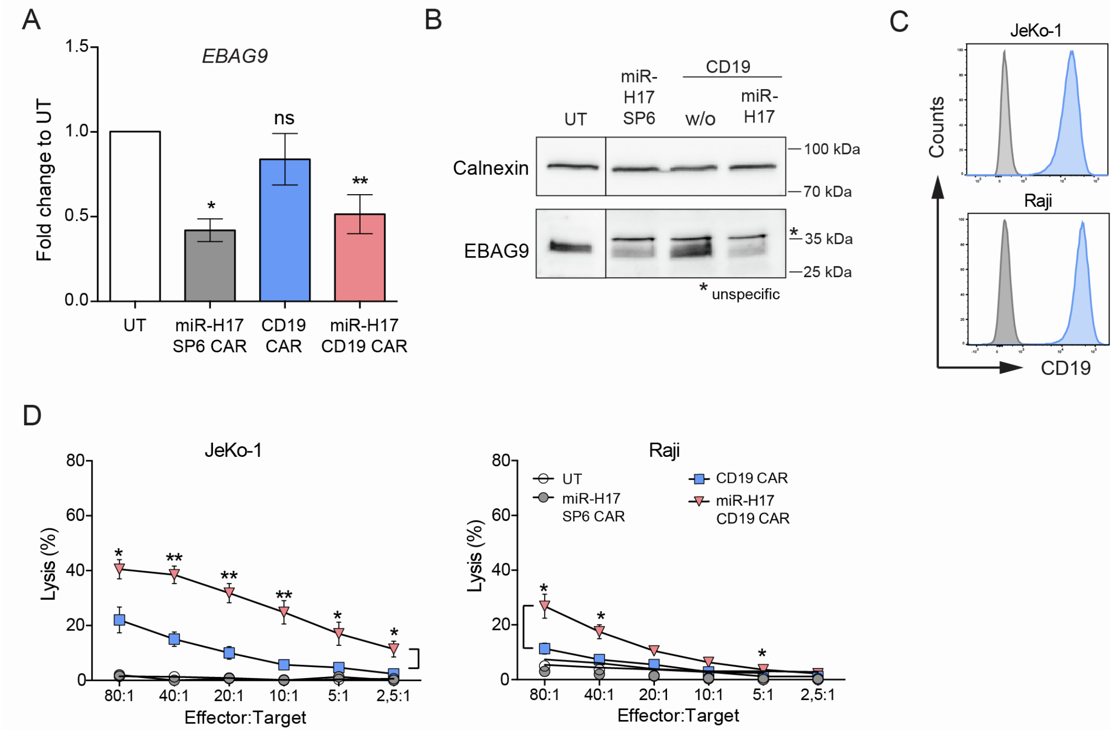

Figure S5: Increasing the cytolytic activity of CAR T cells by silencing EBAG9 is a universally applicable cell biological mechanism

(A) miRNA-mediated reduction of *EBAG9* mRNA-level in human $CD8^+$ T cells by the use of miR-H17. CAR-expressing $CD8^+$ IgG^+ cells were sorted by FACS. Gene expression was determined by qRT-PCR. UT cells were set arbitrarily at 1. Bars represent mean values ± SEM of n=2-4 experiments with n=3 (miR-H17 SP6), n=9 (UT, CD19, miR-H17 CD19) independent donors per group. *p<0.05, **p<0.01; ns, not significant. A one-sample t-test was performed.

(B) Analysis of EBAG9 protein expression in miR-H17 and CAR transduced T cells. CAR-expressing $CD8^+$ IgG^+ T cells were sorted by FACS and analyzed by Western blot. One representative Western blot out of two experiments is shown. Calnexin served as a loading control; * represents an unspecific protein band.

(C) Flow cytometric analysis of CD19 surface expression on the B-NHL cell line JeKo-1 and the B-ALL cell line Raji. A representative histogram for each cell line is shown.

(D) *In vitro* cytotoxicity assays were performed by co-culturing $CD8^+$ CAR T cells for 4 hrs with [^{51}Cr]-labeled CD19-expressing B-NHL cell lines (Jeko-1, Raji) ($5x10^3$ cells each) at the indicated effector to target ratios. Transduction rates of CAR T cells were adjusted to 14% by addition of non-transduced T cells (UT). Data represent mean ± SEM error bars n=3 experiments performed in duplicates with n=3 (miR-H17 SP6) and n=6 (UT, CD19, miR-H17 CD19) different donors per group. *p<0,05, **p<0,01; ns, not significant. A Mann-Whitney U test was employed.

Figure S6

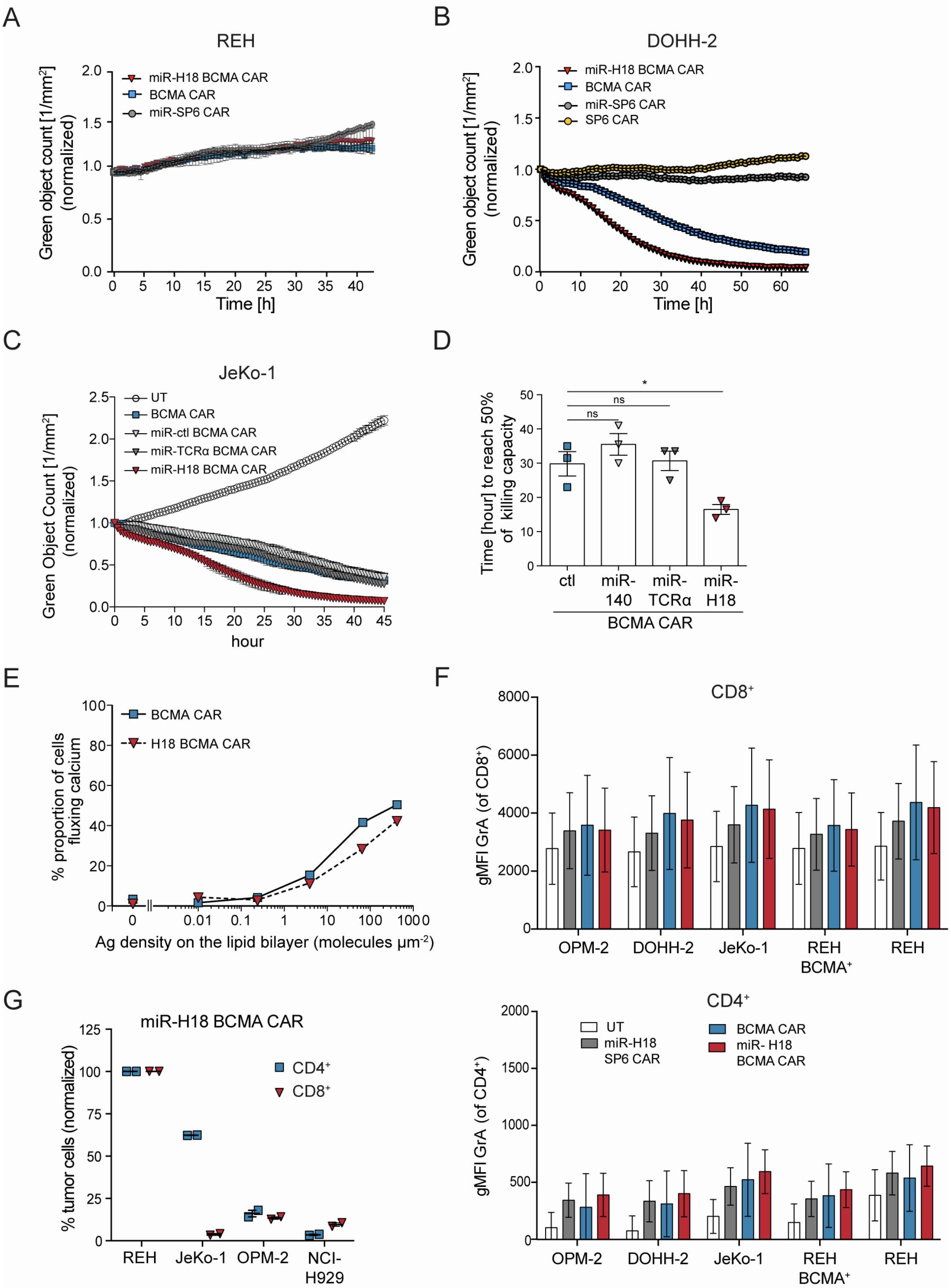

Figure S6: RNAi-mediated increase of CAR T cell cytotoxicity maintains antigen specificity and is selective for EBAG9

(A) The kinetics of CAR T cell cytotoxicity was assessed by analyzing co-cultures of CAR T cells and target cells in an IncuCyte system. As a control, CAR T cells were

co-cultured with BCMA-negative REH cells stably expressing GFP at an effector to target ratio of 5:1 for 42 hrs. GFP fluorescence intensity was normalized for time point 0. Data were measured in triplicates and represent mean ± SEM of n=3 experiments with n=6 (UT, miR-H18 SP6) and n=8 (BCMA CAR, miR-H18 BCMA CAR) independent donors per group.

(B) CAR T cell cytotoxicity was determined as in (A) by analyzing co-cultures of CAR T cells and DOHH-2 target cells. Enriched $CD8^+$ CAR T cells were co-cultured with DOHH-2 cells stably expressing GFP at an effector to target ratio of 1:1 for 66.5 h. Target cell GFP fluorescence intensity was measured every 30 min and normalized to the first time point. The fold change in tumor fluorescence intensity from t = 0 to t = 66.5 h is shown and was measured in triplicates. One representative example of 3 independent donors tested is shown. No differences between CAR T cells transduced with either an SP6 CAR or the miR-H18-modulated SP6 CAR could be observed.

(C) $CD8^+$ CAR T cell cytotoxicity was determined as in (B) by analyzing co-cultures of CAR T cells and JeKo-1 target cells. Data were measured in duplicates and represent mean ± SEM of one experiments with n=3 independent donors per group.

(D) Quantification bar plot of the time to reach 50% cytotoxicity of targeted tumor cell lines in (C). Data are shown as mean ± SEM. A unpaired t-test was applied. *$p<0.05$, ns, not significant.

(E) Fura-2-loaded BCMA and miR-H18 BCMA CAR T cells were stimulated on supported lipid bilayers (SLBs) loaded with indicated numbers of fluorescently labeled BCMA antigen molecules. Proportion of cells fluxing calcium were plotted as a function of antigen density (molecules μm^{-2}). Data for one representative donor out of two is shown (n=2 independent experiments). The number of cells assayed for each data point in the shown plot ranged from n=556 to 1434 (median n=700).

(F) Intracellular granzyme A FACS staining of BCMA CAR T cells. $CD8^+$ and $CD4^+$ CAR T cells were co-cultured with different target cell lines at a 1:1 ratio for 2 hrs. Data are shown as mean ± SEM of n=2 experiments with n=5 independent donors per group.

(G) *In vitro* cytotoxicity of $CD4^+$ and $CD8^+$ miR-H18 BCMA CAR T cells against target cells with variable BCMA surface density. T cells were magnetically separated from total miR-H18 BCMA CAR T cells and co-cultured in a 1:1 ratio with $BCMA^{high}$- (OPM-2, NCI-H929) or $BCMA^{low}$ -expressing (JeKo-1) target cells for 72 hrs. The BCMA-negative cell line REH was used as a control and the results were normalized to remaining REH cells in culture. Remaining tumor cells were quantitated by FACS, and data are represented as dot plots with mean ± SEM of n=2 independent donors.

Figure S7

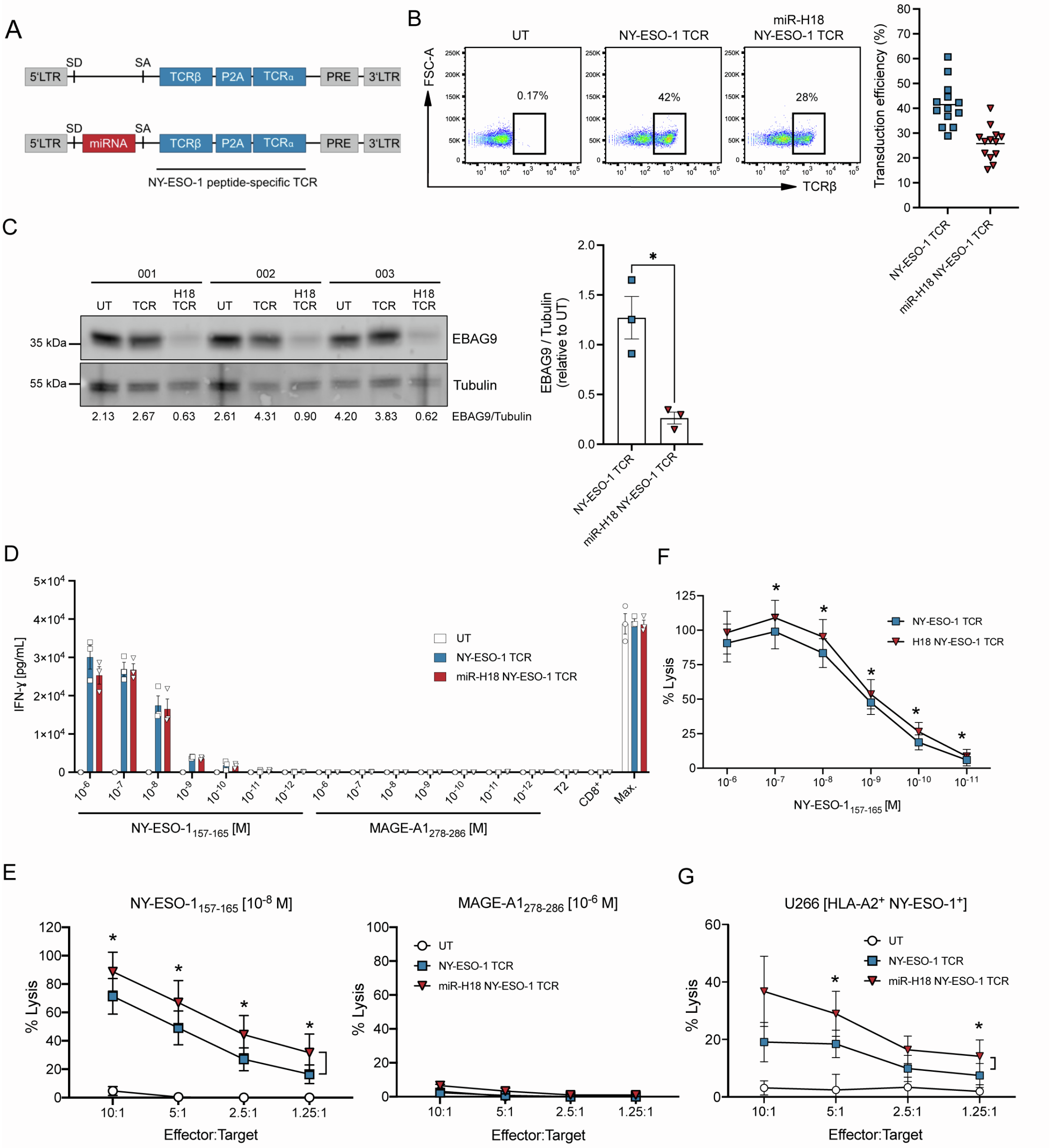

Figure S7: EBAG9 depletion arms CD8^{+} T cells transduced with an NY-ESO-1 TCR with enhanced antigen-specific cytolytic effector functions

A) Schematic depicting ɣ-retroviral constructs (MP71 backbone) encoding an HLA-A*0201-restricted NY-ESO-1 peptide-specific TCR with (miR-H18 NY-ESO-1 TCR, bottom) or without (NY-ESO-1 TCR, top) an intronic miRNA (H18) targeting EBAG9.

B) Representative flow cytometry dot plots (left) and quantitation (right) demonstrating transduction efficiency (%) of enriched $CD8^+$ T cells (>85 % $CD3^+$ $CD8^+$). NY-ESO-1 peptide-specific TCR was detected using an anti-mouse TCRβ antibody targeting the murinized constant region of the TCRβ chain. Depicted populations are $CD3^+CD8^+$ $TCR\beta^+$. Frequencies are indicated as percentages on the gate. UT = untransduced $CD8^+$ T cells. Data represents 13 donors (n=13) assessed in 8 independent experiments.

C) Western blot (left) and densitometry (right) assessing EBAG9 expression of FACS sorted (anti-mouse $TCR\beta^+$) NY-ESO-1 TCR or miR-H18 NY-ESO-1 TCR $CD8^+$ T cells. Densitometry is calculated by dividing signal intensity of EBAG9 expression by the corresponding loading control (Tubulin). Normalized EBAG9 expression in transduced $CD8^+$ T cells from each donor was divided by normalized EBAG9 expression in the corresponding UT control. Data represents 3 donors (n=3). Significance calculated using a paired t-test.

D) Quantitation of IFN-ɣ concentration in cell culture supernatants from 18 h co-cultures of TCR-transduced $CD8^+$ T cells with T2 cells. Target cells were peptide-pulsed with decreasing concentrations of cognate NY-ESO-$1_{157\text{-}165}$, or control MAGE-$A1_{278\text{-}286}$ peptides at the indicated concentrations; effector:target ratio 1:1. Equal proportions of transduced $CD8^+$ T cells from each donor and construct were used. Differences in transduction rates were adjusted using UT $CD8^+$ T cells. All samples were performed in triplicates. Bars represent mean ± SEM of n=3 independent donors per group. Max, stimulation with PMA and ionomycin.

E) Cytolytic capacity of NY-ESO-1 TCR, miR-H18 NY-ESO-1 TCR or untransduced (UT) $CD8^+$ T cells (transduction rate: average n=7, NY-ESO-1 TCR 38%; miR-H18 NY-ESO-1 TCR 26%). Cytotoxicity of NY-ESO-1 TCR-equipped $CD8^+$ T cells was

assessed in co-cultures with T2 cells peptide-pulsed with cognate NY-ESO-$1_{157-165}$ peptide [10^{-8} M] (left panel), control MAGE-$A1_{278-286}$ peptide [10^{-6} M] (right panel) at the indicated E:T ratio for 4 hr. Target cell lysis (%) was quantified by enzymatically detection of LDH released. Equal proportions of transduced $CD8^+$ T cells from each donor and construct were used. Differences in transduction rates were adjusted using UT $CD8^+$ T cells from the same donor. All samples were performed in triplicates. Data represents n=7 donors assessed in 4 independent experiments. Statistics calculated using the Wilcoxon matched-pairs signed rank test.

F) Comparison of cytolytic activity of $CD8^+$ T cells transduced with NY-ESO-1 TCR or miR-H18 NY-ESO-1 TCR constructs. Transduced $CD8^+$ T cells (transduction rate: average n=7 donors; NY-ESO-1 TCR 41.2 %; miR-H18 NY-ESO-1 TCR 26.2 %) were co-cultured with T2 cells peptide-pulsed with decreasing concentrations of cognate NY-ESO-$1_{157-165}$ peptide, E:T of 10:1. Target cell lysis was quantified as in (D). All samples were performed in triplicates. Data represents 7 donors (n=7) assessed in 3 independent experiments. Statistics calculated using the Wilcoxon matched-pairs signed rank test.

G) In vitro cytotoxicity assay of TCR-transduced $CD8^+$ T cells (transduction rate: average n=6 NY-ESO-1 TCR 39.2 %; miR-H18 NY-ESO-1 TCR 23.7 %) at the indicated E:T ratios with the MM cell line U266 (HLA-A*0201^+ and NY-ESO-1^+). Target cell lysis was quantified as in (D). All samples were performed in triplicates. Data represents 6 donors (n=6) assessed in 4 independent experiments. Statistics calculated using the Wilcoxon matched-pairs signed rank test; bars represent mean ± SEM *$p<0.05$.

Figure S8

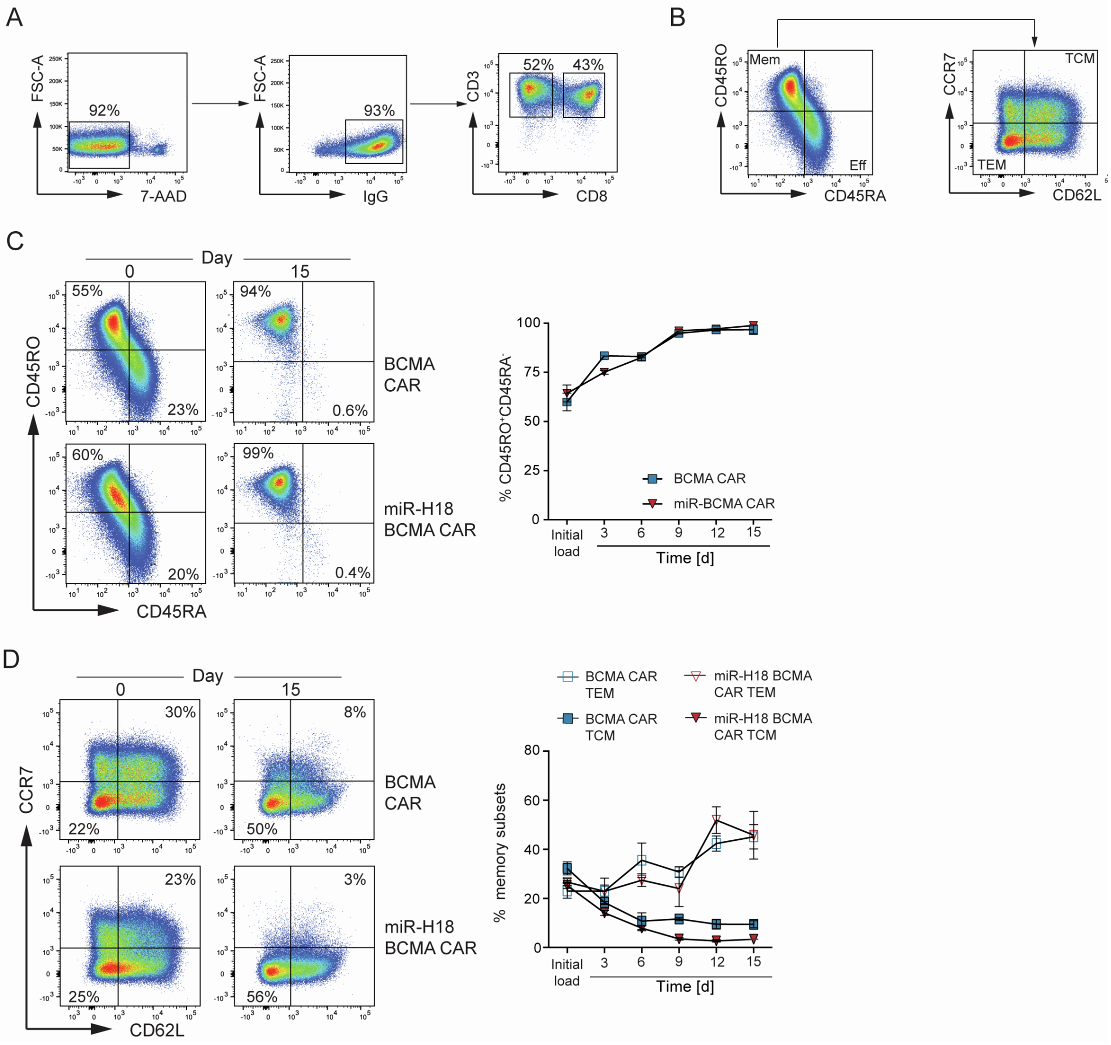

Figure S8: Differentiation of transduced human CAR T cells towards effector memory T cells is not affected by the loss of EBAG9

(A) Gating strategy for flow cytometric analysis of CAR T (IgG^{+}) cells after each round of co-culture with MM.1S target cells. Frequencies are indicated as numbers on the gates.

(B) In every round of co-cultivation, memory T cell subset composition was analyzed by flow cytometry. Gating strategy for the definition of memory subtypes is shown (TEM: CD45RO^{+} CD45RA^{-}, CD62L^{-} CCR7^{-}; TCM: CD45RO^{+} CD45RA^{-}, CD62L^{+} CCR7^{+}). Mem, memory T cells; Eff, T effector cells.

(C) Percentages of memory T cells were determined by analysis of $CD45RO^+$ $CD45RA^-$ cells in one experiment with n=2 independent donors per CAR specificity. Mean values ± SEM are plotted for each CAR. Representative dot plots are shown on the left. Frequencies are indicated as percentages on the gate.

(D) Co-staining of $CD45RO^+$ $CD45RA^-$ cells with CD62L and CCR7 was used to distinguish between central and effector memory T cells in one experiment with n=2 independent donors per CAR specificity. Mean values ± SEM are plotted. Representative dot plots are shown on the left. Frequencies are indicated as percentages on the gate.

Figure S9

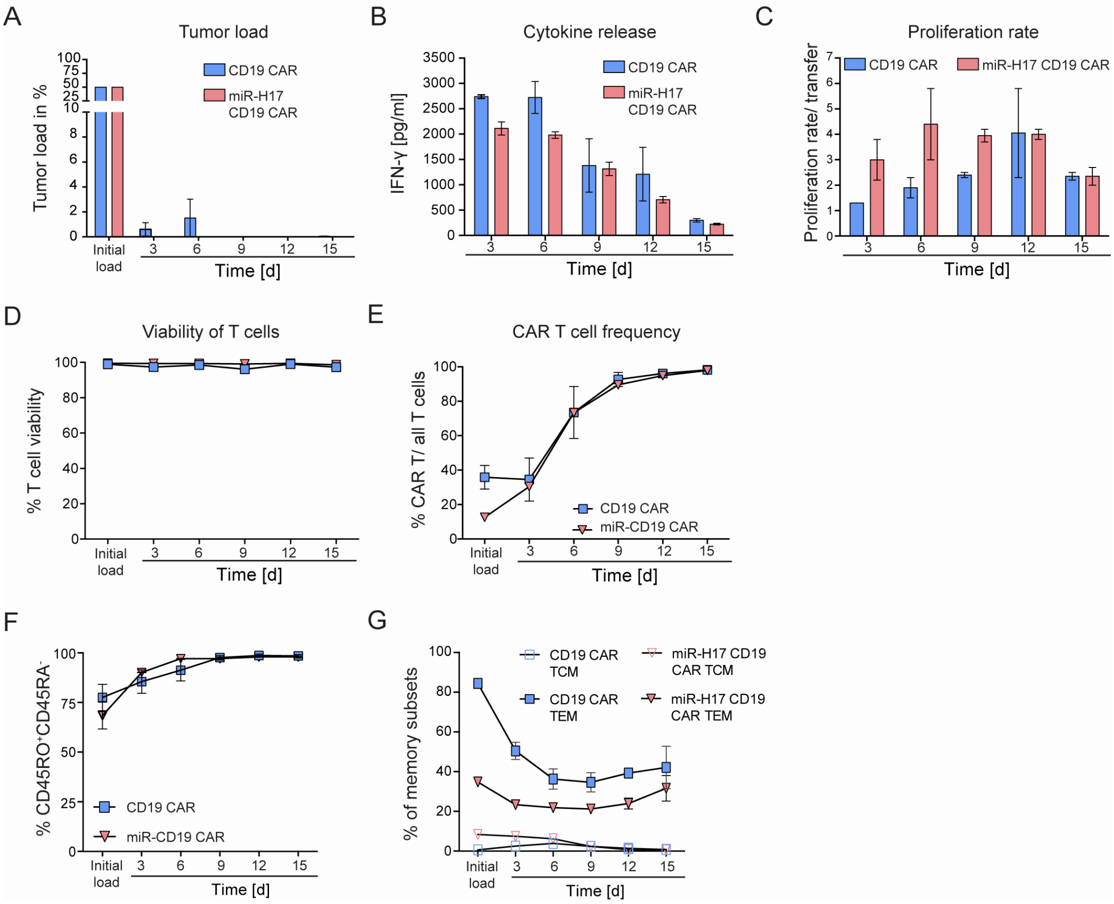

Figure S9: CD19 CAR T cells with silenced EBAG9 maintain their effector functions and proliferative capacity through cycles of repeated antigen exposure

(A)-(G) CD19 CAR T cells and miR-H17 CD19 CAR T cells were co-cultured with JeKo-1 target cells at a 1:1 ratio over a time period of 15 days and five transfer rounds. Every 72 hrs, CAR T cells were transferred to fresh target cells and tumor load (A), IFN-γ secretion (B), proliferation rate (C), CAR T cell viability (D), CAR T cell frequency (E) and memory composition (F, G) were assessed. Data are depicted as mean value ± SEM of n=1 experiment with n=2 independent donors per group.

Figure S10

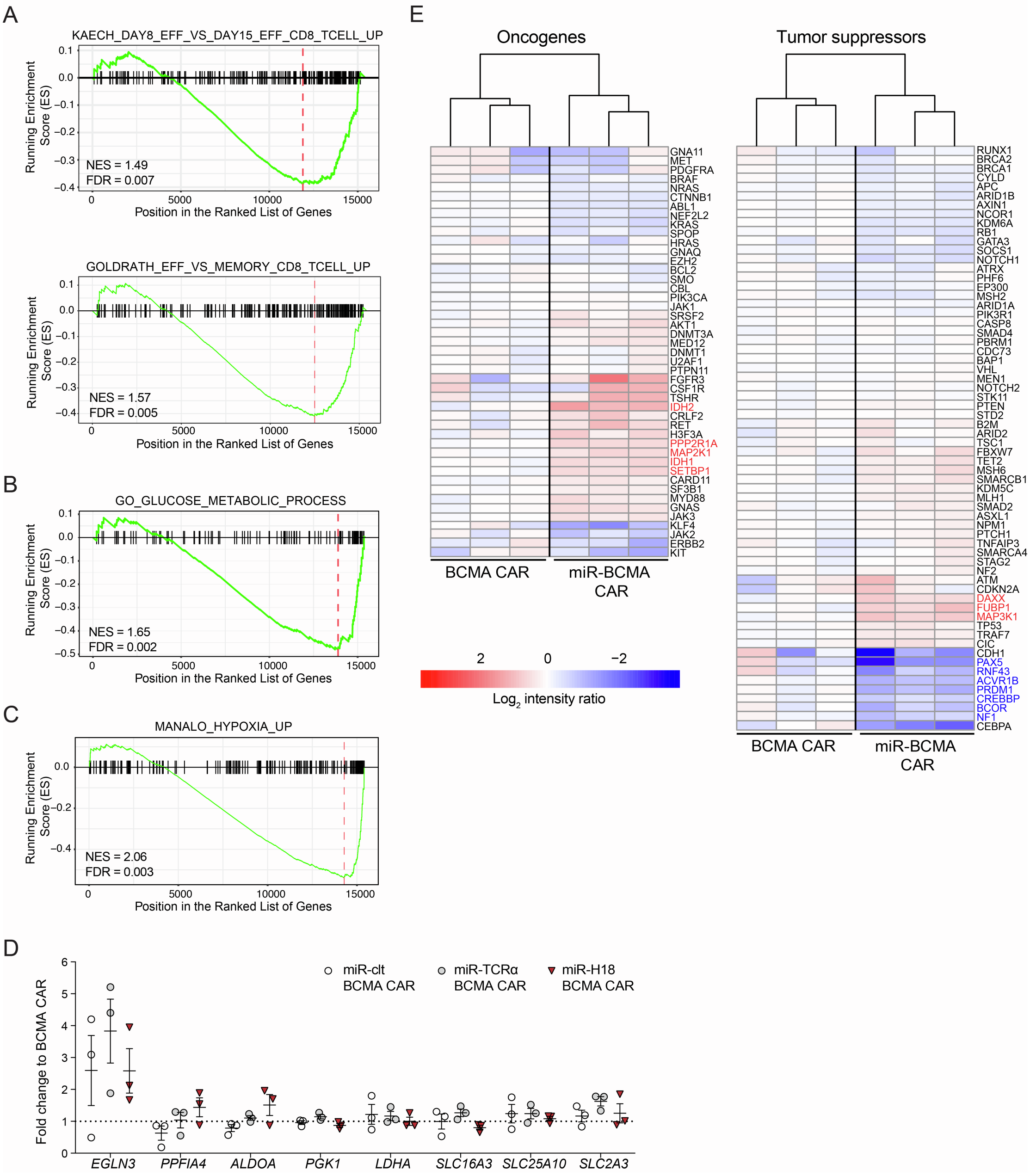

Figure S10: Expression of oncogenes and tumor suppressor genes in BCMA and miR-H18 BCMA CAR T cells

(A) – (C) Representative GSEA results from unbiased functional comparison of BCMA CAR and miR-H18 BCMA CAR transduced T cells using the complete MSigDB (version 6.5). The cutoff of the adjusted *P*-value (for gene lists enrichment significance) was set to 0.2 and 18668 gene lists were tested.

(D) Gene expression analysis of sorted CAR-expressing BCMA-, miR-ctl BCMA-, miR-TCRα BCMA-, and miR-H18 BCMA-CAR T cells at day 10 of culture with supplementation of IL-7 and IL-15. A qRT-PCR assay was applied. BCMA CAR T cells were set arbitrarily at 1, indicated by the dashed horizontal line. Data represent one experiment with n=3 independent donors per group.

(E) Heat maps of oncogenes and tumor suppressor genes that are frequently mutated in various cancer types. Oncogenes or tumor suppressor genes as defined by[1] differentially expressed ($p<0.05$) in miR-H18 BCMA CAR T cells compared to BCMA CAR T cells are marked in red (upregulated) or blue (downregulated). Expression changes are depicted according to the color scale.

Table S1: Exhaustion gene set

Listed are the exhaustion-correlated genes chosen for the transcriptome profiling of the isolated BCMA and miR-H18 BCMA CAR T cells. The genes are grouped and ordered regarding their biological function and differential expression in the miR-H18 BCMA CAR T cell group. The baseMean column shows the average normalized expression (linear scale), lfcShrunk the log2-fold change after shrinkage, wpadj the P value after multiple testing correction, DE the differential expression call (- for downregulated and + for upregulated in the miR-H18 BCMA CAR T cell group, 0 for not altered) and the rank up column the gene's rank when ordered from the signed highest to lowest log2-fold change.

Table S2: Oncogene and tumor suppressor gene set

Listed are oncogenes (top) and tumor suppressor genes (bottom) that are often mutated in various cancer types. The genes were analyzed regarding their differential expression in miRNA-modified BCMA CAR T cells compared to unmodified BCMA CAR T cells. The baseMean column shows the average normalized expression (linear scale), lfcShrunk the log2-fold change after shrinkage, wpadj the P value after multiple testing correction, DE the differential expression call (- for downregulated and + for upregulated in the miR-H18 BCMA CAR T cell group, 0 for not altered) and the rank up column the gene's rank when ordered from the signed highest to lowest log2-fold change.

Supplemental Methods
Reagents Table

REAGENT or RESOURCE	SOURCE	IDENTIFIER
**Antibodies**		
Ultra-LEAF Purified anti-mouse CD3 (clone: OKT3)	BioLegend	Cat # 317326; RRID:AB_11150592
Ultra-LEAF Purified anti-mouse CD28 (clone: CD28.2)	BioLegend	Cat # 102116; RRID:AB_11147170
Ultra-LEAF Purified anti-mouse CD16/32 (clone: 93)	BioLegend	Cat# 101330, RRID:AB_2561482
PE anti-mouse CD4 (clone: GK1.5)	BioLegend	Cat# 100408, RRID:AB_312693
BV421 anti-mouse CD8a (clone: 53-6-7)	BioLegend	Cat# 100738, RRID:AB_11204079
PB anti-mouse CD8a (clone: 53-6-7)	BioLegend	Cat# 100725, RRID:AB_493425
Ultra-LEAF Purified anti-human CD3 (clone: OKT3)	BioLegend	Cat# 300332, RRID:AB_11150396
Ultra-LEAF Purified anti-human CD28 (clone: CD28.2)	BioLegend	Cat# 302934, RRID:AB_11148949
PB anti-human CD3 (clone: HIT3a)	BioLegend	Cat# 300330, RRID:AB_10551436
BV421 anti-human CD4 (clone: OKT4)	BioLegend	Cat# 317434, RRID:AB_2562134
BV421 anti-human CD4 (clone: RPA-T4)	BioLegend	Cat# 300532, RRID:AB_10965645
APC anti-human CD8a (clone: HIT8a)	BioLegend	Cat# 300912, RRID:AB_314116
PE/Cy7 anti-human CD8 (clone: SK1)	BioLegend	Cat# 344712, RRID:AB_2044008
APC anti-human CD19 (clone: HIB19)	BioLegend	02212, RRID:AB_314242
PB anti-human CD45RA (clone: HI100)	BioLegend	Cat# 304117, RRID:AB_493656
PerCP/Cy5.5 anti-human CD45 RO (clone: UCHL1)	BioLegend	Cat# 304221, RRID:AB_1575041
FITC anti-human CD62L (DREG-56)	BioLegend	Cat# 304804, RRID:AB_314464
PE/Cy7 anti-human CD107a/LAMP1 (clone: H4A3)	BD Biosciences	Cat# 561348, RRID:AB_10644018

BV421 anti-human CD138 (clone: MI35)	BioLegend	Cat# 356516, RRID:AB_2562660
PE anti-human CD197/CCR7 (G043H7)	BioLegend	Cat# 353204, RRID:AB_10913813
AF647 anti-human CD223/LAG-3 (clone: 11C3C65)	BioLegend	Cat# 369304, RRID:AB_2566480
APC anti-human CD269/BCMA (clone: 19F2)	BioLegend	Cat# 357506, RRID:AB_2562889
PE anti-human CD279/PD-1 (clone: EH12.2H7)	BioLegend	Cat# 329906, RRID:AB_940483
BV421 anti-human CD366/TIM-3 (clone: F38-2E2)	BioLegend	Cat# 345008, RRID:AB_11218598
PE IgG (polyclonal)	Southern Biotech	Cat # 2040-09
PE anti-human granzyme A (clone: CB9)	BioLegend	Cat# 507206, RRID:AB_315472
FITC anti-mouse TCR β chain (clone: H57-597)	BioLegend	Cat#109205
Calnexin polyclonal antibody	Enzo Life Science	Cat# ADI-SPA-860-D, RRID:AB_2038898
hFAB Rhodamine Anti-Tubulin antibody	BIO-RAD	12004166
StarBright Blue 700 Goat Anti-Rabbit IgG	BIO-RAD	12004162
EBAG9 polyclonal antibody	in-house	2
**Bacterial and Virus Strains**		
E. coli XL1 Blue	Internal stock	N/A
**Biological Samples**		
Human PBMCs, Buffy Coats, Leukapheresis Material	Voluntary healthy donors	N/A
**Chemicals, Peptides, and Recombinant Proteins**		
RPMI-1640 Medium	GIBCO	Cat #11530586
DMEM Medium	GIBCO	Cat #11995073
Fetal Bovine Serum (South Africa)	PAN-Biotech	Cat #P30-1502
Fetal Bovine Serum	GIBCO	Cat #26140079
Penicillin-Streptomycin (100x)	GIBCO	Cat #11548876
Sodium pyruvate (100x)	GIBCO	Cat #12539059
L-glutamine (100x)	GIBCO	Cat #13462629
Minimum essential medium non-essential amino acids (100x)	GIBCO	Cat #11350912

2-Mercaptoethanol	Thermo Fisher Scientific	Cat #11528926
Recombinant Mouse IL-2	PeproTech	Cat #212-12
Recombinant Human IL-2	PeproTech	Cat #200-02
Recombinant Human IL-7	Miltenyi Biotec	Cat #130-095-363
Recombinant Human IL-15	Miltenyi Biotec	Cat #130-095-765
Dynabeads Mouse T-Activator CD3/CD28	Thermo Fisher Scientific	Cat #114.52D
Protamine sulfate	Sigma Aldrich	Cat #1101230005
RetroNectin	TaKaRa	Cat #T202
BioColl	Biochrom	Cat #BS.L 6115
Monensin/Golgi-Stop	BD Bioscience	Cat #554724
Brefeldin A	Sigma Aldrich	Cat #B7651
Ionomycin	Calbiochem	Cat #407952
Phorbol-12-myristate-13-acetate (PMA)	Promega	Cat #V1171
eFluor670	eBioscience	Cat #65-0840-85
Luciferin	Biosynth	Cat #FL08607
[^{51}Cr] sodium chromate	Perkin Elmer	Cat #NEZ030002MC
N$^{\alpha}$Benzyloxycarbonyl-L-lysine Thiobenzyl Ester	Merck Millipore	Cat #200274
5,5`-Dithio-*bis*-(2-nitrobenzoic acid)	Sigma Aldrich	Cat #D8130
Pierce ECL Western Blotting Substrate	Thermo Fisher Scientific	Cat #32106
Bovine Serum Albumin	Sigma Aldrich	Cat #05470
FastDigest ECoRI	Thermo Fisher Scientific	Cat #FD0274
FastDigest NotI	Thermo Fisher Scientific	Cat #FD0594
Thermosensitive alkaline phosphatase (Fast-AP)	Thermo Fisher Scientific	Cat #EF0651
T4 DNA Ligase	Thermo Fisher Scientific	Cat #15224041
SuperScript III First-Strand Synthesis SuperMix for qRT-PCR	Thermo Fisher Scientific	Cat #11752050
TaqMan Fast Advanced Master Mix	Thermo Fisher Scientific	Cat #4444557
D$^{\mathbf{b}}$-restricted peptide HY antigen: WMHHNMDLI	Biosyntan, Berlin	N/A

Immunodominant peptide IV, SV40 large T antigen VVYDFLKL	JPT Peptide Technologies GmbH, Berlin	N/A
HLA-A2*0201 restricted peptide NY-ESO-1 (157-165): SLLMWITQC	Discovery Peptides, Billingham, UK	N/A
HLA-A2*0201 restricted peptide MAGE-A1 (278-286): KVLEYVIKV	Discovery Peptides, Billingham, UK	N/A
**Critical Commercial Assays**		
TruSeq Stranded mRNA Library Prep Kit	Illumina	Cat #20020594
Easy Sep Human T Cell Isolation Kit	STEMCELL	Cat #17951
CD8 T cell Isolation Kit, human	Miltenyi Biotech	Cat #130-096-495
CD138 MACS Micro Beads	Miltenyi Biotec	Cat #130-051-301
LS columns	Miltenyi Biotec	Cat #130-042-401
Easy Sep PE Positive Selection Kit II	STEMCELL	Cat #17684
FIX&PERM Cell Permeabilization Kit	Molecular Probes	Cat #GAS003
QIAquick Gel Extraction Kit	QIAGEN	Cat #28706
RNeasy Mini Kit	QIAGEN	Cat #74104
CytoTox 96 Non-Radioactive Cytotoxicity Assay	Promega	Cat# G1780
**Deposited Data**		
RNAseq data are deposited at Genbank/ENA; Primary Accession code: PRJEB37843; Secondary Accession code: ERP121180		
**TaqMan Primer**		
Ebag9	Thermo Fisher Scientific	Mm01167189_mH
EBAG9	Thermo Fisher Scientific	Hs00188444_m1
Gapdh	Thermo Fisher Scientific	Mm99999915_g1
GAPDH	Thermo Fisher Scientific	Hs02786624_g1
EGLN3	Thermo Fisher Scientific	Hs00222966_m1
PPFIA4	Thermo Fisher Scientific	Hs00949811_m1
ALDOA	Thermo Fisher Scientific	Hs00605108_g1
PGK1	Thermo Fisher Scientific	Hs00943178_g1
LDHA	Thermo Fisher	Hs01378790_g1

	Scientific	
SLC16A3	Thermo Fisher Scientific	Hs00358829_m1
SLC25A10	Thermo Fisher Scientific	Hs00201730_m1
SLC2A3	Thermo Fisher Scientific	Hs00359840_m1
SDHA	Thermo Fisher Scientific	Hs07291714_mH
**Experimental Models: Cell Lines**		
NCI-H929	DSMZ, Braunschweig	Cat# ACC-163, RRID:CVCL_1600
OPM-2	DSMZ, Braunschweig	Cat# ACC-50, RRID:CVCL_1625
DOHH-2	DSMZ, Braunschweig	Cat# ACC-47, RRID:CVCL_1179
JeKo-1	DSMZ, Braunschweig	Cat# ACC-553, RRID:CVCL_1865
Jurkat J76	DSMZ, Braunschweig	Cat# ACC-282, RRID:CVCL_0065
REH	Dr. Stephan Mathas, MDC, Berlin	N/A
MM.1S luc eGFP	(Oden et al., 2015) [3]	N/A
HEK-293T	Quantum Biotechnologies	N/A
293VecGalV	BioVec Pharma	[4]
PlatE	Dr. Wolfgang Uckert, MDC	[5]
Co16.113	Dr. Gerald Willimsky, Charité, Berlin	N/A
B3Z	Dr. Wolfgang Uckert, MDC	[6]
Raji	(Oden et al., 2015) [3]	ATCC #CCL-86
U-266	DSMZ, Braunschweig	Cat# ACC-9
T2	Dr. Simone Rhein, MDC, Berlin	Cat# ACC-598
**Experimental Models: Organisms/Strains**		
NOD.Cg-Prkdcscid Il2rg tm1 Wji/Szj (NSG) mice	The Jackson Laboratories	Cat# JAX:005557, RRID:IMSR_JAX:005557
C57BL/6N mice	Charles River	N/A

Rag2$^{-/-}$ mice	Charles River	N/A
Ebag9 -/-	[7]	N/A
**Recombinant DNA**		
pALF-10A1GaV	Internal stock	N/A
pcDNA3.1-MLV gag/pol	Dr. Wolfgang Uckert, MDC	N/A
MP71_GFP	Dr. Wolfgang Uckert, MDC	[8]
MP71_miR-ctl_GFP	This manuscript	N/A
MP71_miR-M1-M5_GFP	This manuscript	N/A
MP71_miR-H16-H19_GFP	This manuscript	N/A
MP71_BCMA CAR	[9]	N/A
MP71_miR-ctl_BCMA CAR	This manuscript	N/A
MP71_miR-TCRα_BCMA CAR	This manuscript	N/A
MP71_miR-H18_BCMA CAR	This manuscript	N/A
MP71_CD19 CAR	[10]	N/A
MP71_miR-H17_CD19 CAR	This manuscript	N/A
MP71_SP6 CAR	[9]	N/A
MP71_miR-H17/18_SP6 CAR	This manuscript	N/A
MP71_NY-ESO-1TCR	This manuscript	N/A
MP71_miR-H18 NY-ESO-1 TCR	This manuscript	N/A
**Software and Algorithms**		
FlowJo v. 10.0.8 software	Tree Star	https://www.flowjo.com
Prism version 6.0, 9.0	Graphpad Software Inc.	https://www.graphpad.com
R version 3.6.1	R Core Team	https://www.R-project.org/.
Bioconductor 3.9	Bioconductor	https:/bioconductor.org
Salmon version 0.14.1	[11]	https:// github.com/COMBINE-lab/salmon
Tximeta version 1.5.6	[12]	https:/bioconductor.org
DESeq version 1.24.0	[13]	https:/bioconductor.org

clusterProfiler version 3.12.0	14	
Living Image software version 4.5	Caliper Life Sciences	https:/www.perkinelmer.com

Supplemental references

1. Vogelstein, B, Papadopoulos, N, Velculescu, VE, Zhou, S, Diaz, LA, Jr., and Kinzler, KW (2013). Cancer genome landscapes. *Science* **339**: 1546-1558.
2. Engelsberg, A, Hermosilla, R, Karsten, U, Schulein, R, Dorken, B, and Rehm, A (2003). The Golgi protein RCAS1 controls cell surface expression of tumor-associated O-linked glycan antigens. *J Biol Chem* **278**: 22998-23007.
3. Oden, F, Marino, SF, Brand, J, Scheu, S, Kriegel, C, Olal, D*, et al.* (2015). Potent anti-tumor response by targeting B cell maturation antigen (BCMA) in a mouse model of multiple myeloma. *Mol Oncol* **9**: 1348-1358.
4. Ghani, K, Wang, X, de Campos-Lima, PO, Olszewska, M, Kamen, A, Riviere, I*, et al.* (2009). Efficient human hematopoietic cell transduction using RD114- and GALV-pseudotyped retroviral vectors produced in suspension and serum-free media. *Hum Gene Ther* **20**: 966-974.
5. Morita, S, Kojima, T, and Kitamura, T (2000). Plat-E: an efficient and stable system for transient packaging of retroviruses. *Gene Ther* **7**: 1063-1066.
6. Karttunen, J, Sanderson, S, and Shastri, N (1992). Detection of rare antigen-presenting cells by the lacZ T-cell activation assay suggests an expression cloning strategy for T-cell antigens. *Proc Natl Acad Sci U S A* **89**: 6020-6024.
7. Ruder, C, Hopken, UE, Wolf, J, Mittrucker, HW, Engels, B, Erdmann, B*, et al.* (2009). The tumor-associated antigen EBAG9 negatively regulates the cytolytic capacity of mouse CD8+ T cells. *J Clin Invest* **119**: 2184-2203.
8. Engels, B, Cam, H, Schuler, T, Indraccolo, S, Gladow, M, Baum, C*, et al.* (2003). Retroviral vectors for high-level transgene expression in T lymphocytes. *Hum Gene Ther* **14**: 1155-1168.
9. Bluhm, J, Kieback, E, Marino, SF, Oden, F, Westermann, J, Chmielewski, M*, et al.* (2018). CAR T Cells with Enhanced Sensitivity to B Cell Maturation Antigen for the Targeting of B Cell Non-Hodgkin's Lymphoma and Multiple Myeloma. *Mol Ther* **26**: 1906-1920.
10. Lamers, CH, Sleijfer, S, Vulto, AG, Kruit, WH, Kliffen, M, Debets, R*, et al.* (2006). Treatment of metastatic renal cell carcinoma with autologous T-lymphocytes genetically retargeted against carbonic anhydrase IX: first clinical experience. *J Clin Oncol* **24**: e20-22.
11. Patro, R, Duggal, G, Love, MI, Irizarry, RA, and Kingsford, C (2017). Salmon provides fast and bias-aware quantification of transcript expression. *Nat Methods* **14**: 417-419.
12. Love, MI, Soneson, C, Hickey, PF, Johnson, LK, Pierce, NT, Shepherd, L*, et al.* (2020). Tximeta: Reference sequence checksums for provenance identification in RNA-seq. *PLoS Comput Biol* **16**: e1007664.
13. Ignatiadis, N, Klaus, B, Zaugg, JB, and Huber, W (2016). Data-driven hypothesis weighting increases detection power in genome-scale multiple testing. *Nat Methods* **13**: 577-580.

14. Yu, G, Wang, LG, Han, Y, and He, QY (2012). clusterProfiler: an R package for comparing biological themes among gene clusters. *OMICS* **16**: 284-287.
